# Champacyclin, a New Cyclic Octapeptide from *Streptomyces* Strain C42 Isolated from the Baltic Sea

**DOI:** 10.3390/md11124834

**Published:** 2013-12-02

**Authors:** Alexander Pesic, Heike I. Baumann, Katrin Kleinschmidt, Paul Ensle, Jutta Wiese, Roderich D. Süssmuth, Johannes F. Imhoff

**Affiliations:** 1 Technical University of Berlin, Institute for Chemistry, Straße des 17. Juni 124, Berlin 10623, Germany; E-Mails: pesic@chem.tu-berlin.de (A.P.); ensle@chem.tu-berlin.de (P.E.); 2 GEOMAR Helmholtz Center for Ocean Research Kiel, Marine Microbiology, Düsternbrooker Weg 20, Kiel 24105, Germany; E-Mails: hbaumann@geomar.de (H.I.B.); kkleinschmidt@geomar.de (K.K.); jwiese@geomar.de (J.W.)

**Keywords:** *Streptomyces champavatii*, antimicrobial activity, fire blight, cyclic peptides, sequence ladder analysis, chiral GC-PCI/EI-MS, ESI-Ion-Trap-MS*^n^*, solid phase peptide synthesis (SPPS), on-resin cyclization, 2D NMR

## Abstract

New isolates of *Streptomyces champavatii* were isolated from marine sediments of the Gotland Deep (Baltic Sea), from the Urania Basin (Eastern Mediterranean), and from the Kiel Bight (Baltic Sea). The isolates produced several oligopeptidic secondary metabolites, including the new octapeptide champacyclin (**1a**) present in all three strains. Herein, we report on the isolation, structure elucidation and determination of the absolute stereochemistry of this isoleucine/leucine (Ile/Leu = Xle) rich cyclic octapeptide champacyclin (**1a**). As 2D nuclear magnetic resonance (NMR) spectroscopy could not fully resolve the structure of (**1a**), additional information on sequence and configuration of stereocenters were obtained by a combination of multi stage mass spectrometry (MS*^n^*) studies, amino acid analysis, partial hydrolysis and subsequent enantiomer analytics with gas chromatography positive chmical ionization/electron impact mass spectrometry (GC-PCI/EI-MS) supported by comparison to reference dipeptides. Proof of the head-to-tail cyclization of (**1a**) was accomplished by solid phase peptide synthesis (SPPS) compared to an alternatively side chain cyclized derivative (**2**). Champacyclin (**1a**) is likely synthesized by a non-ribosomal peptide synthetase (NRPS), because of its high content of (d)-amino acids. The compound (**1a**) showed antimicrobial activity against the phytopathogen *Erwinia amylovora* causing the fire blight disease of certain plants.

## 1. Introduction

The actinobacterial genus *Streptomyces* is widely distributed in both terrestrial and aquatic ecosystems. These bacteria exhibit diverse physiological and metabolic properties, such as the production of extracellular enzymes and the synthesis of a large variety of secondary metabolites, a number of which are exploited by the pharmaceutical industries. Many of these antimicrobials are of low molecular weights (≤2000 Da) and belong to macrolides, polyenes, tetracyclines, amino glycosides, or other compound classes with heterogeneous chemical structures. *Streptomycetes* also produce a large variety of oligo- and polypeptides including signaling pheromones and a range of antimicrobial peptides with potential lead structures used for chemical synthesis of derivatives. Biosynthesis of peptides occurs either ribosomally or non-ribosomally contributing to the chemical diversity of secondary metabolites synthesised by these microorganisms. Ribosomally synthesized and posttranslationally modified peptides (RiPPs) have an increased variation of chemical structures due to post-translational modification. Structures of non-ribosomally synthesized peptides (NRPs) are different from the ribosomal peptides by the frequent occurrence of (d)-amino acids and otherwise modified non-proteinogenic amino acids. Furthermore, the structural complexity can be dramatically increased if fatty acids and/or carbohydrate moieties are present in these molecules [1]. One characteristic property of peptides is their possibility to form cyclic structures. Notable important cyclic (depsi)-peptides are cyclosporine (immunosuppressant), polymyxin B (antibiotic), didemnin B (antitumor), gramicidin S (antibiotic), and valinomycin (ionophore), as reviewed by Bockus *et al.* (2013) [2].

Due to their potential for use as drugs, there is a high demand not only to screen for new potent cyclic peptides but also for fast and reliable methods to characterize them. In particular alternative methods are needed, if analytical methods such as NMR are not applicable, due to low quantities of the substances available. For peptides and proteins, *de novo* sequencing by tandem mass spectrometric techniques is still an indispensable tool. These fragmentation techniques include collision induced fragmentation (CID) and electron capture dissociation (ECD). Routinely b- and y-ions are annotated in linear peptides, while for cyclic peptides additional fragmentation pathways in tandem mass spectrometry (MS^2^) experiments are possible, yielding also non-direct sequence (NDS) ions through a scrambling of the amino acid sequence, which can be difficult to interpret [3].

Kjeldsen *et al.* (2003) [4] used a variant of electron-capture dissociation (ECD), named “Hot ECD” (HECD), which induces *w*-type fragment ions through a high collision energy secondary fragmentation process to distinguish between Leu and Ile. However, HECD is not applicable to most mass spectrometers and requires state-of-the-art mass spectrometers. To overcome this problem Armirotti *et al.* (2007) [5] introduced a low-energy ESI-Ion-TRAP-MS*^n^* method to distinguish between Leu and Ile. It was demonstrated that in a series of fragmentation steps, Ile, unlike Leu, generates a 69 Da ion by fragmentation of its 86 Da immonium ion. However, with regard to sensitivity, every MS*^n^* step dramatically decreases the ion abundance.

An alternative to sequencing of peptides by tandem mass spectrometry constitutes partial hydrolysis or partial enzymatic digestion to yield overlapping amino acid sequences (*sequence ladder*) of smaller peptides. This method proved an excellent tool, when coupled to a mass spectrometer equipped with an electron impact (EI) ionization source [6]. Mass spectrometric sequence analyses of oligopeptide fragments (up to nine amino acids) were performed after derivatization yielding the corresponding *N*-trifluoroacetyl peptide alkyl esters and direct injection into the EI ion-source [6]. In some cases, in addition to the sequence information, the distinction between Xle residues was possible due to different ion fragments formed in the EI-ion source (Supplementary Figure S47).

As mentioned above, cyclic peptides may also contain (d)-amino acids making enantiomer analytics indispensable. Data about the content of (d)- and (l)-amino acids in a peptide can be routinely generated by total hydrolysis of the protein (6 M HCl, 110 °C, 24 h, vacuum) and subsequent chiral gas-chromatography mass spectrometry analysis (GC-MS). To assign the configuration at ambiguous positions, again partial hydrolysis, preferably yielding dipeptides, combined with chiral GC-MS analysis has to be carried out. Accordingly, Pätzold *et al.* (2006) [7] have extensively studied gas chromatographic separation of stereoisomers of dipeptides. It should, however, be pointed out, that it is impossible to separate all possible 39^2^ dipeptides (1520 stereoisomers plus the achiral Gly-Gly) on a chiral column.

**Figure 1:**
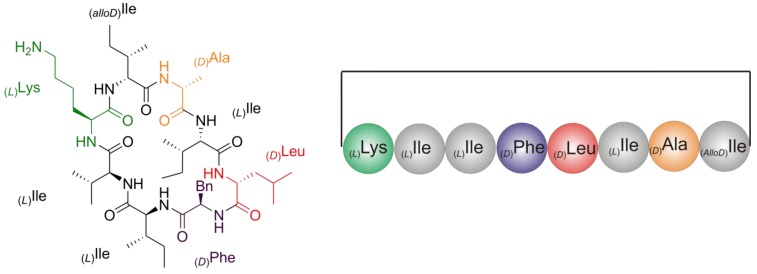
Structure of head-to-tail-cyclized octapeptide champacyclin (**1a**) isolated from *Streptomyces* spp. C42.

In the present work, a cyclic antimicrobial octapeptide was isolated from new isolates of *Streptomyces* closely related to *Streptomyces champavatii*. *Streptomyces champavatii* was first isolated from Champavathi river mud (Andhra Pradesh) and Bangalore virgin soil (Mysore State) in India (Narasimha Rao and Uma, 1958) [8]. Three isolates of this bacterium were identified by paper chromatography, where two compounds are antifungal heptaene antibiotics, which were termed afterwards champamycin A and champamycin B with heptane group characteristic absorption curves (λ_max_ = 360, 380, 405 nm), and a third antifungal compound named champavatin with no selective absorption in the region of 250–400 nm [8,9], of which chemical structure was not, thus far, determined. Here we report on the structure and biological activities of a cyclic octapeptide named champacyclin (**1a**) (Figure 1), which is considered to correspond to the third previously unknown component reported by Rao and Narasimha Rao (1967) [9]. Recent database searches (Dictionary of Natural Products, DNP) for compound (**1a**) revealed no structural identity to known compounds. Therefore, a full structure determination of (**1a**) was performed as described in the sections below. The assignment of the isoleucine/leucine (Ile/Leu = Xle) residues as well as the absolute stereochemistry to distinct positions in the cyclic peptides was a challenging task, which was solved by a combination of mass spectrometry, 2D NMR spectroscopy and synthetic approaches. Champacyclin (**1a**) shows activity against the fire blight plant pathogen *Erwinia amylovora* in the µM-range.

## 2. Results and Discussion

### 2.1. Isolation and Identification of *Streptomyces* Strains

Spore forming actinobacteria were isolated from sediments of the Gotland Deep (Baltic Sea), from the eastern Mediterranean (Urania Basin) and from the Kiel Bight, respectively. Three strains were identified as members of the genus *Streptomyces* and were further characterized. Strain C42 was isolated from a sediment core of the Gotland Deep, strain i6a from the Urania Basin, and strain XX19 from the Kiel Bight.

**Figure 2:**
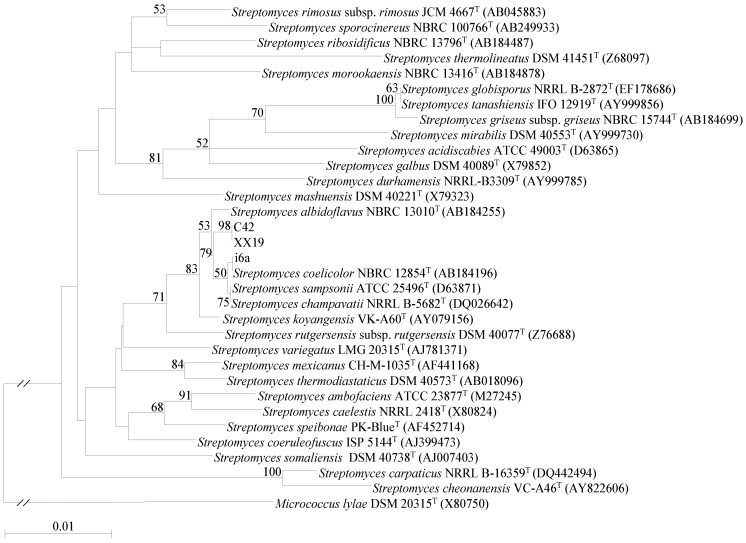
Phylogenetic tree of *Streptomyces* sp. strains C42, XX19, and i6a including type strains of closest relatives, further members of the genus *Streptomyces* and *Micrococcus lylae* as outgroup. The calculation was performed using the maximum-likelihood estimation (MLE). Bootstrap values are given in percent (only numbers above 50% are shown).

The 16S rRNA gene sequences of the three strains displayed highest similarity to the closely related type strains of *Streptomyces coelicolor* NBRC 12854^T^ (99.7%/100%), *Streptomyces albidoflavus* NBRC 13010^T^ (99.7%/100%), *Streptomyces champavatii* NRRL B-5682^T^ (99.6%/99.9%), *Streptomyces sampsonii* ATCC 25496^T^ (99.7%/99.9%), and *Streptomyces daghestanicus* NRRL B-5418^T^ (99.4%/99.8%). Strains C42 and XX19 had identical 16S rDNA sequences. All three strains were found in a clade together with *S. coelicolor*, *S. champavatii*, *S. sampsonii* and *S. albidoflavus* (Figure 2). These species have highly similar 16S rRNA gene sequences of >99.9% similarity, indicating a difference of less than one out of 100 nucleotides. Based on the genotypic data of multilocus sequence analysis (MLSA) and DNA-DNA hybridization (DDH), combined with key phenotypic properties, it was proposed that these *Streptomyces* species of the *S. albidoflavus* clade, including *S. champavatii*, should be merged into a single genomic species, for which the name *S. albidoflavus* was proposed [10].

Despite the phylogenetic considerations, according to their metabolic profiles, our isolates clearly grouped with *S. champavatii*. Three main compounds were produced by these strains, which were previously mentioned as metabolites of *S. champavatii* [9,10]: the champamycin A and champamycin B with absorption maxima (λ_max_ = 362, 384 and 407 nm) characteristic for the heptaene group and a third compound, which we named champacyclin (**1a**).

The strains C42 and XX19 showed a positive PCR amplification product of the adenylation domain (A) from a NRPS complex, which is an indicator of non-ribosomal peptide synthesis. Every NRPS module contains an adenylation domain (A), which activates the corresponding amino acids, and results after severe reactions in amino acid assembly. Therefore, the correlation between the positive PCR product of the A domain and the isolated compound (**1a**) from the fermented culture broth is not possible without extensive investigations by sequencing of the biosynthesis gene cluster.

Champacyclin (**1a**) was found in extracts of both, culture supernatant (using ethyl acetate) and biomass (using methanol). Higher amounts were present in the cells compared to the culture medium. The methanolic extracts of *S. champavatii* strain C42 were separated by reversed-phase HPLC on a C18 column, and the elution profiles were studied with HPLC-DAD-ESI-(+)-MS. On the basis of similar absorption properties, we assume that (**1a**) corresponds to the third component found but not characterized by Narasimha Rao and Uma (1958) [9].

**Figure 3:**
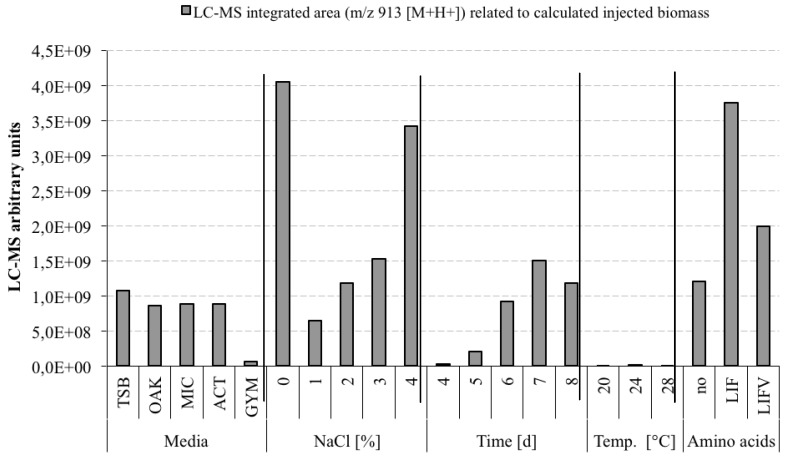
Optimization of growth conditions for champacyclin (**1a**) biosynthesis of strain C42. In each experiment base parameters were maintained except for one parameter, which was variable in the experiment: 100 mL culture volume, actinomycetes medium except in the salt experiments GYM medium was used, 1.5% salt (NaCl), six days incubation time, 28 °C incubation temperature, 125 rpm shaking, and methanolic extracts. The results are comparable within each experiment series. Amino acids (1 mg/mL supplement): no = no supplementation, LIFV = _(*L*)_leucine (L), _(*L*)_isoleucine (I), _(*L*)_phenylalanine (F) and _(*L*)_valine (V). Temp. = Temperature. Media abbreviations see text.

Strain C42 was selected for growth experiments in order to increase the production of (**1a**) and to decrease that of co-produced metabolites (Figure 3). Growth parameters varied in these experiments were the growth medium (tryptic soy broth (TSB), oak flakes medium (OAK), *Micromonospora* medium (MIC), actinomycetes medium (ACT), and GYM medium (GYM)), the salt concentration (0%–4% NaCl in 1% intervals), the incubation temperature (20 °C, 24 °C, and 28 °C), the incubation time (one day–eight days in one-day intervals) and supplementation with a mixture of amino acids (_(*L*)_leucine (L), _(*L*)_isoleucine (I), _(*L*)_phenylalanine (F), and _(*L*)_valine (V)). Champacyclin (**1a**) was produced in all five growth media, though the metabolite profiles varied with the medium. The best production was found in the actinomycetes medium (5.6 g wet weight biomass of 100 mL culture), where at the same time the production of the heptaenes was lowest. The highest yield of (**1a**) was obtained in media without NaCl and after incubation for seven days at 24 °C. A further increase in the production of compound (**1a**) was obtained by addition of a mixture of the amino acids leucine, isoleucine, and phenylalanine, in concentrations of 1 mg/mL each to the medium (Figure 3).

The *Streptomyces* isolates showed antimicrobial activities, which resemble those reported for the Indian strains of *S. champavatii*. Antimicrobial activity was found with crude extracts and purified substance. The crude extracts of all isolated strains (C42, XX19, and i6a) inhibited the yeast *Candida glabrata*. The purified substance (**1a**) showed a 40% inhibition of the fire blight pathogen *Erwinia amylovora* at a concentration of 25 µM. There was no activity observed against the test strains *Bacillus subtilis*, *Escherichia coli* K-12, *Staphylococcus lentus*, *Pseudomonas syringae* pv*. aptata*, *Pseudomonas fluorescens*, *Xanthomonas campestris*, and *Ralstonia solanacearum* (DSM 9544).

### 2.2. Structure Elucidation of Champacyclin (**1a**)

The octapeptide champacyclin (**1a**) was isolated in amounts of 2.2 mg/L. UV-DAD-data were in support of the presence of a peptide with an absorption at λ_max_ = 258 nm (ε 1523 in MeOH) and the occurrence of aromatic amino acids within the structure. Subsequent High-Resolution Electrospray Orbitrap Mass Spectrometry (HR-ESI-(+)-Orbitrap-MS) showed a molecular mass at *m*/*z* 912.62574 [M + H]^+^ (relative mass error Δ_m_ = −2.572 ppm, *calcd*. 912.62809 [M + H]^+^), which corresponds to the calculated tentative molecular formula of [C_48_H_82_N_9_O_8_] [M + H]^+^ with 13 DBE for the uncharged molecule.

#### 2.2.1. Quantitative Chiral Amino Acid Analysis

**Figure 4:**
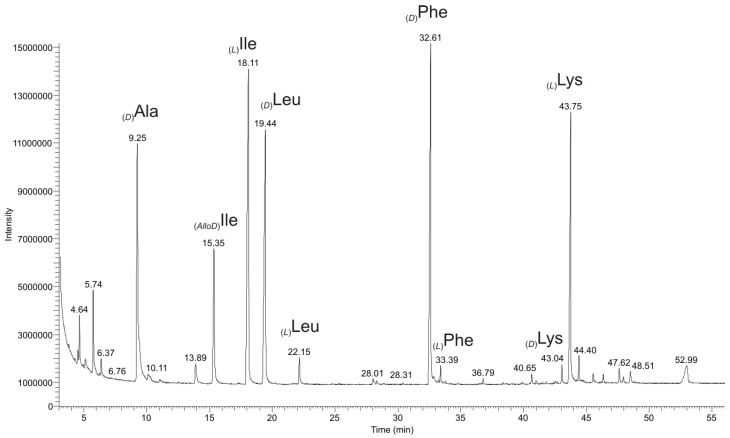
Chiral GC-PCI-MS analysis of the amino acid hydrolyzate of champacyclin (**1a**) on a Chirasil^®^-(l)-Val column. Amino acids were analyzed as their *N*-pentafluoropropionic acid 2-propyl esters and identified by their [M + H]^+^-ions.

Chiral Gas Chromatography Positive Chemical Ionization Mass Spectrometry (GC/PCI-MS) of the total hydrolyzate of (**1a**) on a Chirasil^®^-(l)-Val column was performed to quantify the amino acids and to evaluate the presence of (d)-amino acids. Derivatization of the amino acids to the *N*-pentafluoropropionic acid 2-propyl esters, yielded the following analytical results with GC-MS (Figure 4): _(*D*)_Ala, _(*AlloD*)_Ile, _(*L*)_Ile, _(*D*)_Leu, _(*D*)_Phe, and _(*L*)_Lys. The ratio of the peak areas of _(*AlloD*)_Ile and _(*L*)_Ile is 1:2.8, corresponding to a ratio of 1:3 (Supplementary Table S6). Additionally, small amounts of racemic _(*L*)_Leu (6% relative to _(*D*)_Leu), _(*D*)_Lys (5% relative to _(*L*)_Lys), and _(*L*)_Phe (3.2% relative to _(*D*)_Phe) were detected (Figure 4 and Supplementary Table S6). By comparison of the peak areas (Supplementary Figure S13 and Table S6) of GC-MS chromatograms we deduced the amino acid content: _(*D*)_Ala, _(*AlloD*)_Ile, _(*L*)_Ile, _(*D*)_Leu, _(*D*)_Phe, and _(*L*)_Lys as 1:1:3:1:1:1. Summing up all molecular masses of these amino acids combined to the restriction to only 13 DBE (as derived from the HR-MS data), the molecular mass of the peptide (**1a**) is only compatible with a cyclic peptide structure (Figure 1). Such a cyclic peptide still has various possibilities for the amino acid sequence as well as for cyclization. For the latter there exist two possibilities, *i.e.*, through a head-to-tail ring formation between N^α^_(*L*)_Lys^1^-CO-_(*AlloD*)_Ile^8^ as in (**1a**) (Figure 1) or a head-to-side-chain ring formation as represented in (**2**) (synthetic derivative, Supplementary Figure S3) between N^ζ^-_(*L*)_Lys-CO-_(*AlloD*)_Ile^8^ (Supplementary Figure S3).

#### 2.2.2. Mass Spectrometry

**Figure 5:**
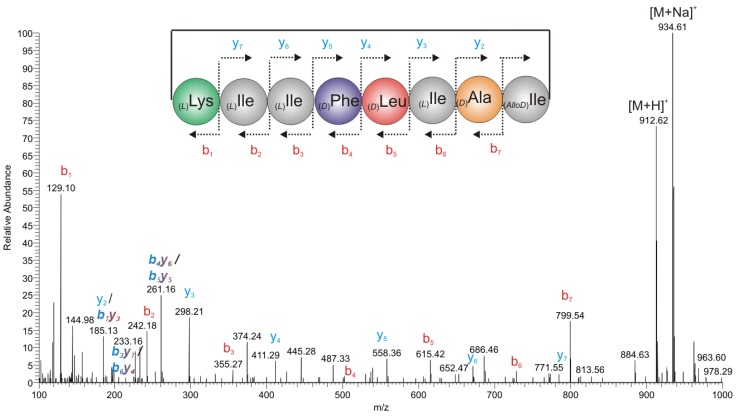
HR-ESI-(+)-SID-Orbitrap-MS of champacyclin (**1a**) with assignment of characteristic fragments according to the Roepstorff nomenclature [11].

In order to obtain the primary sequence of the peptide, HR-ESI-(+)-Orbitrap-In-Source-fragmentation (SID) spectra (Figure 5) were measured, which yielded preferably the y- and b-ion fragments according to the Roepstorff nomenclature for peptide fragmentation [11]. In combination with the results from the amino acid analysis (Figure 4) the primary sequence of the peptide could be established as: [_(*L*)_Lys^1^-_(*x*)_Xle^2^-_(*x*)_Xle^3^-_(*D*)_Phe^4^-_(*x*)_Xle^5^-_(*x*)_Xle^6^-_(*D*)_Ala^7^-_(*x*)_Xle^8^] with *x* = *d*, *l*, or *Allod* leaving the identity and the stereochemistry of five amino acids of the Leu/Ile-(Xle)-type undetermined.

In order to discriminate between Ile/Leu (Xle) in the y_2_/*b_7_y_3_* 185 Da ion (Figure 5) we performed SID-MS*^n^*-experiments (Supplementary Figure S2) on the Orbitrap ESI-(+)-mass spectrometer using an ion-trap analyzer. The subsequent MS*^n^*-studies (transition 185 Da → 86 Da → 69 Da) showed the 69 Da ion characteristic of Ile, as reported by Armirotti *et al.* (2006) [5]. For both y_2_/*b_7_y_3_* ions, fragmentation mechanisms have been formulated (Supplementary Figure S2C) indicating the presence of an _(*x*)_Ile^6,8^ at position 6 and/or 8.

#### 2.2.3. Chiral GC-PCI/EI-MS Analysis of the Partial Hydrolyzate of (**1a**)

Due to the high abundance of the isobaric amino acids _(*D*)_Leu (1x), _(*L*)_Ile (3x), and _(*AlloD*)_Ile (1x) in (**1a**), the assignment of the configuration of amino acids in distinct positions of the peptide was a challenging task. The problem was solved by performing partial hydrolysis of (**1a**) to generate an array of overlapping sequences based on dipeptides. These dipeptides were analyzed by means of chiral GC-PCI/EI-MS and identified by their [M + H]^+^ and [M + C_2_H_5_]^+^ molecular ions, respectively. According to Pätzold *et al.* (2006) [7], the best separations for dipeptides were obtained for *N*-trifluoroacetyl methyl esters on a Chirasil^®^-(l)-Val column (max. operating temperature 195 °C). From various hydrolysis times and reaction temperatures we established 6 M HCl (200 μL) for 7 h at 110 °C to yield satisfying amounts of the desired dipeptides of (**1a**). In order to avoid diketopiperazine formation mild derivatization conditions were used as previously reported by Pätzold *et al.* (2006) [7]. Due to the high-energy fragmentation in the PCI- and the EI-mode, we observed complex but characteristic fingerprint fragmentation patterns. Additionally, the ion intensities in the milder PCI-mode allowed distinguishing between isobaric dipeptides (Supplementary Information).

At this point of the structure elucidation a series of dipeptides as reference compounds was synthesized to give proof of all possible retention times and fragmentation patterns of the individual dipeptides. The sequences of the synthetic dipeptides are as follows: Xle-Ala/Ala-Xle (Supplementary Figures S31–S38), Xle-Phe/Phe-Xle (Supplementary Figures S39–S46), and Xle-Xle (Supplementary Figures S23–S30). With the careful analysis of the chromatograms and mass spectra in analogy to Weygand *et al.* (1965) [6], we could distinguish between all isobaric dipeptides and even predict whether Xle within the dipeptide is located at the *N*-terminal or *C*-terminal position. These interpretations are based on the fact that only dipeptides with Xle at the *C*-terminus show a characteristic fragment peak at *m/z* 86 in EI mode. For Xle at the *N*-terminal position of the dipeptides the fragment ion *m/z* 183 is indicative and subsequent fragmentation of this ion allows the differentiation between Ile (183 Da ion → 154 Da ion) and Leu (183 Da ion → 140 Da ion).

**Figure 6:**
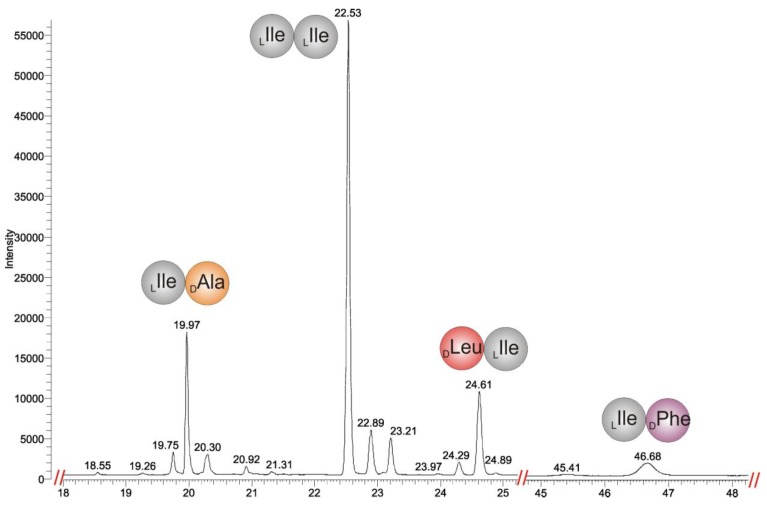
GC-SIM-Chromatogram on a Chirasil^®^-(l)-Val column of champacyclin (**1a**) after *N*-trifluoroacetyl methyl ester derivatization. *R_t_* (min): 19.97 _(*L*)_Ile-_(*D*)_Ala, 22.53 _(*L*)_Ile-_(*L*)_Ile, 24.61 _(*D*)_Leu-_(*L*)_Ile, 46.68 _(*L*)_Ile-_(*D*)_Phe.Assignment of stereochemistry with reference dipeptides summarized in Table 1, based on the most abundant peaks in partial hydrolyzate of (**1a**). Additional peaks correspond to side products from racemization of the dipeptides during partial hydrolysis and derivatization, respectively.

Figure 6 shows the single ion monitoring (SIM) chromatogram for the chromatographic separation of dipeptide derivatives from in the partial hydrolyzate of (**1a**) on a Chirasil^®^-(l)-Val column. The analysis of the fragmentation patterns proved the presence of the following dipeptides in the partial hydrolyzate of the natural product (**1a**): _(x)_Ile-_(*D*)_Phe, _(x)_Ile-_(*D*)_Ala _(*x*)_Ile^x^-_(*x*)_Ile^x^, and _(*D*)_Leu^x^-_(*x*)_Ile^x^ (Supplementary Figures S15–S22). The derivatized dipeptides _(*L*)_Lys^1^-_(*x*)_Xle^2^/_(*x*)_Xle^8^-_(*L*)_Lys^1^ were not detected from the partial hydrolyzate (**1a**), perhaps they are not eluting from the column at the operating temperature (195 °C) of the GC column or derivatization of lysine side chain amino group N^ζ^H_2_ remained incomplete under the conditions applied. Combining the analysis from chiral GC/PCI-EI-MS of the partial hydrolyzate of (**1a**) with the synthetic reference dipeptides, the partial sequence _(*L*)_Lys^1^-_(*x*)_Xle^2^-_(*x*)_Ile^3^-_(*D*)_Phe^4^-_(*x*)_Xle^5^-_(*x*)_Ile^6^-_(*D*)_Ala^7^-_(*x*)_Xle^8^ could be derived leaving the assignment of the dipeptides _(*x*)_Ile^x^-_(*x*)_Ile^x^ and _(*D*)_Leu^x^-_(*x*)_Ile^x^ within the sequence unclear.

Subsequently we focused on the elucidation of the remaining absolute stereochemistry of the amino acids in dipeptides of the partial hydrolyzate of (**1a**) (Figure 6). To minimize the amount of dipeptides necessary for stereochemical elucidation and to reduce difficulties in chiral separation on the Chirasil^®^-(l)-Val column, we focused on the observed configurations of the amino acids according to the previous chiral amino acid analysis after hydrolysis and *N*-pentafluoropropionic acid 2-propylester derivatization (Figure 4) and considered the sequence, estimated by MS fragmentation pattern analysis (Figure 5) and partial hydrolysis approach. Table 1 shows the synthetic and the observed *N*-trifluoroacetyl dipeptide methyl ester in partial hydrolyzate of (**1a**) after GC-MS derivatization. Based on Table 1, the assignment of the correct stereochemistry in Figure 6 was established only for the most abundant peaks in partial hydrolyzate of (**1a**).

Dipeptides with identical retention times are shown in bold in Table 1. Unequivocal assignment was achieved for the dipeptide derivatives [*R_t_* (min), synthetic reference/partial hydrolysis of (**1a**)]: _(*L*)_Ile-_(*D*)_Phe (46.75/46.68) as well as _(*L*)_Ile-_(*D*)_Ala (20.00/19.97). Consequently one additional building block is _(*D*)_Leu-_(*L*)_Ile (24.75/24.61) and the last building block is _(*L*)_Ile-_(*L*)_Ile (22.55/22.53). As a consequence we postulated _(*AlloD*)_Ile^8^ at position 8.

**Table 1:** Gas-chromatographic enantioseparation of synthetic peptides and of peptides from the partial hydrolyzate of champacyclin (**1a**) as *N*-trifluoroacyl dipeptide methyl esters on a Chirasil^®^-(l)-Val column.

Synthetic Dipeptides	Retention Times *R_t_* (min) of Synthetic Dipeptides	Dipeptides from the Partial Hydrolyzate (1a)	Retention Times *R_t_* (min) of Dipeptides from the Partial Hydrolyzate (1a)
_(*L*)_**Ile**-_(*D*)_**Ala**	**20.00**	_(*L*)_**Ile**-_(*D*)_**Ala**	**19.97**
_(*AlloD*)_Ile-_(*D*)_Ala	20.35		
_(*L*)_Ile-_(*L*)_Ala	20.40		
_(*AlloD*)_Ile-_(*L*)_Ala	20.45		
_(*L*)_**Ile**-_(*L*)_**Ile**	**22.55**	_(*L*)_**Ile**-_(*L*)_**Ile**	**22.53**
_(*AlloD*)_Ile-_(*L*)_Ile	23.40		
_(*L*)_Ile-_(*AlloD*)_Ile	22.65		
_(*AlloD*)_Ile-_(*AlloD*)_Ile	22.90		
_(*L*)_Ile-_(*D*)_Leu	24.30		
_(*L*)_Ile-_(*L*)_Leu	23.75		
_(*AlloD*)_Ile-_(*D*)_Leu	24.90		
_(*AlloD*)_Ile-_(*L*)_Leu	25.05		
_(*D*)_**Leu**-_(*L*)_**Ile**	**24.75**	_(*D*)_**Leu**-_(*L*)_**Ile**	**24.61**
_(*D*)_Leu-_(*AlloD*)_Ile	24.00		
_(*L*)_Leu-_(*AlloD*)_Ile	23.30		
_(*L*)_Leu-_(*L*)_Ile	22.05		
_(*D*)_Leu-_(*D*)_Leu	25.90		
_(*D*)_Leu-_(*L*)_Leu	26.20		
_(*L*)_Leu-_(*L*)_Leu	24.25		
_(*L*)_Leu-_(*D*)_Leu	24.75		
_(*L*)_**Ile**-_(*D*)_**Phe**	**46.75**	_(*L*)_**Ile**-_(*D*)_**Phe**	**46.68**
_(*AlloD*)_Ile-_(*D*)_Phe	44.80		
_(*L*)_Ile-_(*L*)_Phe	40.90		
(AlloD)Ile-_(*L*)_Phe	49.30		

#### 2.2.4. Nuclear Magnetic Resonance (NMR) Spectroscopy

**Figure 7:**
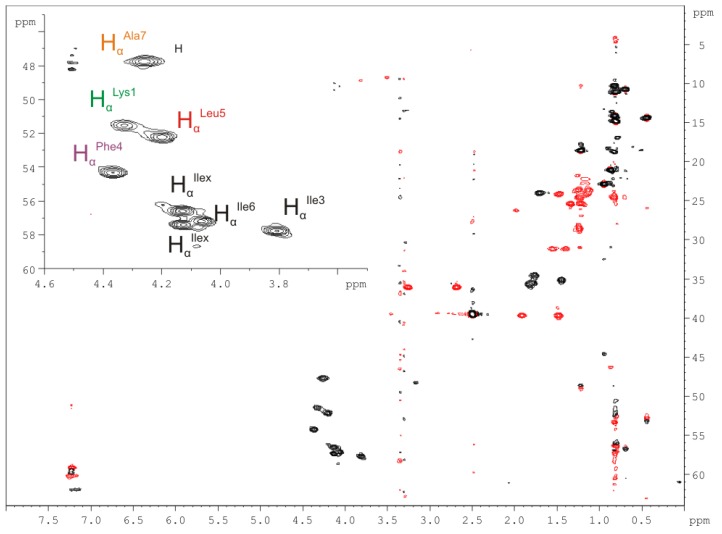
Phase-sensitive DEPT-180-^1^H-^13^C-HMQC NMR data for champacyclin (**1a**) measured in *d*_6_-DMSO on a 600 MHz spectrometer. Correlations from protons with ^1^*J* coupling are visible. Black: CH and CH_3_-groups show positive signals, Red: CH_2_-groups show negative signals.

The partial hydrolysis approach could not unambiguously resolve the positions of _(*L*)_Ile^x^-_(*L*)_Ile^x^ and _(*D*)_Leu^x^-_(*L)*_Ile^x^ in (**1a**). To overcome the uncertainty in the assignment of dedicated amino acids of the _(*x*)_Xle-type to the amino acid sequence we performed 1D and 2D-NMR experiments in *d_6_*-DMSO on a 600 MHz NMR spectrometer for (**1a**). A full spectral data set was acquired comprising ^1^H-NMR, ^1^H-^1^H-COSY, ^1^H-^1^H-NOESY (mixing time 100 ms), ^1^H-^1^H-TOCSY (spin lock 60 ms), ^1^H-^13^C-HMBC, ^1^H-^13^C-HMQC, HMQC-TOCSY (spin lock 80 ms), and DEPT-180-^1^H-^13^C-HMQC. Interpretation of data begun with assignment of the individual amino acids from the eight signals attributed to the eight H_α_ protons in the DEPT-180-^1^H-^13^C-HMQC (Figure 7) and assignment of their correlations in the ^1^H-^1^H-COSY (Supplementary Figure S9) and ^1^H-^1^H-TOCSY spectra (Supplementary Figure S11). The presence of eight H_α_ protons in the amino acid building blocks, indicates the absence of symmetry and according to the dispersion of the chemical shift of their signals (not random coil chemical shifts), (**1a**) is an unsymmetrical cyclopeptide, as already discussed previously.

The phase-sensitive DEPT-180-^1^H-^13^C-HMQC data provided additional information on the CH_3_-, CH_2_- and CH-groups (Figure 7). To avoid overlap of signals, as five out of eight amino acids are _(*x*)_Xle-type amino acids, HMQC-TOCSY data were used for individual assignment (Supplementary Figure S7). Only one single Leu-residue (_(*D*)_Leu^5^) at position 5 could be assigned, meaning that the remaining four _(*x*)_Xle amino acids are _(*x*)_Ile residues, which is also in accordance with the quantitative GC-MS results (Figure 4, Supplementary Table S6).

In the ^1^H-^1^H-COSY spectrum (Supplementary Figure S9) the _(*D*)_Ala^7^ H_α_ proton (4.28 ppm) shows a correlation to the H_β/β′/β″_ protons of the CH_3_ group (1.25 ppm), while three H_α_ protons, _(*L*)_Lys^1^ (4.34 ppm), _(*D*)_Phe^4^ (4.33 ppm), _(*D*)_Leu^5^ (4.22 ppm), show correlations to the H_β/β′_ protons of their corresponding CH_2_-groups (H_β/β′__(*L*)_Lys^1^ (1.55/1.40 ppm), H_β/β′__(*D*)_Phe^4^ (3.29/2.70 ppm), H_β/β′__(*D*)_Leu^5^ (1.93/1.49 ppm)). The remaining four H_α_ protons, _(*x*)_Ile^x^ (4.15 ppm), _(*x*)_Ile^3^ (3.78 ppm), _(*x*)_Ile^6^ (4.05 ppm), and _(x)_Ile^x^ (4.14 ppm) show correlations to the H_β_ protons of their corresponding CH-groups (H_β__(*x*)_Ile^x^ (1.85 ppm), H_β__(*x*)_Ile^3^ (1.47 ppm), H_β__(*x*)_Ile^6^ (1.82 ppm), H_β__(*x*)_Ile^x^ (1.85 ppm)).

**Figure 8:**
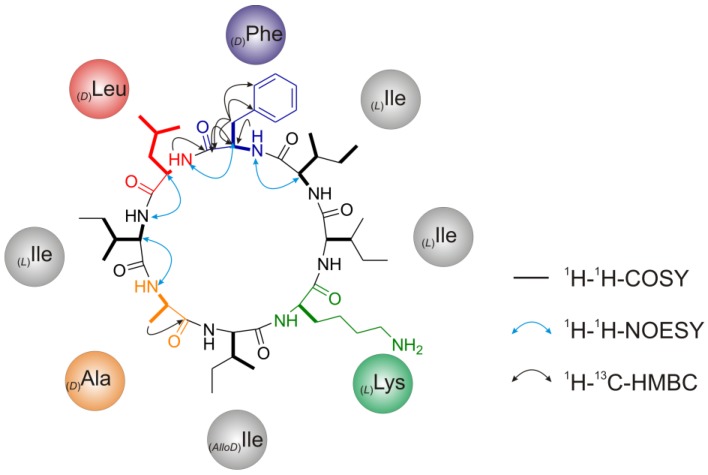
NMR-spectroscopic structure assignment of champacyclin (**1a**) based on correlations between nuclei. Bold lines represent ^1^H-^1^H-COSY-, black arrows ^1^H-^13^C-HMBC- and blue arrows ^1^H-^1^H-NOESY-contacts. Proposed structure of (**1a**) is also based on data from chiral GC/PCI-EI-MS and HR-ESI-(+)-MS*^n^* analysis.

Due to the low amount of (**1a**), ^1^H-^13^C-HMBC data could not be used for sequence-specific assignment of residues but helped in the unambiguous assignment of _(*D*)_Leu to position 5 (Figure 8). Further, sequential data were assigned using the *sequential walk* along the peptide backbone with intra-residual and inter-residual H_N_(i) ↔ H_α_(i) and H_α_(i) ↔ H*_N_*(I + 1) couplings derived from ^1^H-^1^H-COSY and ^1^H-^1^H-NOESY spectra, respectively (Supplementary Figures S9 and S10). Hence, only a stretch of amino acid sequence on the basis of chiral analysis and the *sequential walk* could be established: [_(*L*)_Ile^3^-_(*D*)_Phe^4^-_(*D*)_Leu^5^-_(*L*)_Ile^6^-_(*D*)_Ala^7^], leaving the complete assignment of _(*x*)_Xxx^1,2,8^ (Xxx = Xle, _(*L*)_Lys) by NMR within the sequence undetermined.

The results of NMR interpretation are summarized in Table 2. The NMR results are not complete but helped in the assignment of _(*D*)_Leu^5^ at position 5. Therefore, the necessity of a _(*x)*_Ile at position 8 of the peptide sequence is a consequence of quantitative GC-MS results and the 2D NMR data. Through exclusion, the stereochemistry of the remaining _(*x)*_Ile^8^ was deduced to be _(*AlloD*)_Ile^8^, as mentioned before. Taking all results so far the final cyclic sequence and stereochemistry of champacyclin (**1a**) is deduced as: [_(*L*)_Lys^1^-_(*L*)_Ile^2^-_(*L*)_Ile^3^-_(*D*)_Phe^4^-_(*D*)_Leu^5^-_(*L*)_Ile^6^-_(*D*)_Ala^7^-*_(AlloD)_*Ile^8^].

**Table 2:** ^1^H- and ^13^C-NMR data for champacyclin (**1a**) in *d_6_*-DMSO on a 600 MHz NMR spectrometer at 30 °C.

Amino Acid/Position	^1^H NMR δ(^1^H) in ppm	^13^C NMR δ(^13^C) in ppm (mult.) *	Amino Acid/Position	^1^H NMR δ(^1^H) in ppm	^13^C NMR δ(^13^C) in ppm (mult.) *
*Lys^(1)^*			*Leu^(5)^*		
C=O	−	*n.d.*	C=O	−	*n.d.*
NH	7.40	−	NH	7.77	−
H^α^	4.34	51.5, CH	H^α^	4.22	52.7, CH
H^β/β′^	1.55/1.40	31.6, CH_2_	H^β/β′^	1.93/1.49	39.7, CH_2_
H^γ/γ′^	*n.d.*	*n.d.*	H^γ^	1.72	24.1, CH
H^δ/δ′^	*n.d.*	*n.d.*	H^δ/δ′^	0.96/0.89	22.9/21.4, CH_2_
H^ε/ε′^	*n.d.*	*n.d.*			
NH_2_	*n.d.*	*n.d.*			
*Ile^(x)^*			*Ile^(6)^*		
C=O	−	*n.d.*	C=O	−	*n.d.*
NH	*n.d.*	−	NH	6.91	−
H^α^	4.15	57.1, CH	H^α^	4.05	57.7, CH
H^β^	1.85	35.6, CH	H^β^	1.82	35.5, CH
H^γ^	0.84	14.7, CH_3_	H^γ^	0.80	*n.d.*
H^γ′^	*n.d.*	*n.d.*	H^γ′^	1.23/1.13	23.5, CH_2_
H^δ^	*n.d.*	*n.d.*	H^δ^	*n.d.*	*n.d.*
*Ile^(3)^*			*Ala^(7)^*		
C=O	−	171.4	C=O	−	172.4
NH	7.93	−	NH	7.78	−
H^α^	3.78	57.7, CH	H^α^	4.28	47.7, CH
H^β^	1.47	35.5, CH	H^β/β′/β′′^	1.25	18.4, CH_3_
H^γ^	0.45	14.9, CH			
H^γ′^	1.22/0.83	24.1, CH_2_			
H^δ^	0.72	10.7, CH_3_			
Phe^(4)^			*Ile^(x)^*		
C=O	−	171.0	C=O	−	*n.d.*
NH	8.40	−	NH	*n.d.*	−
H^α^	4.33	54.8, CH	H^α^	4.14	57.9, CH
H^β/β′^	3.29/2.70	36.6, CH_2_	H^β^	1.85	35.6, CH
H^γ^	−	138.3, CH	H^γ^	*n.d.*	*n.d.*
H^δ/δ′^	7.20	129.4, CH	H^γ′^	1.34/1.22	25.3, CH_2_
H^ε/ε′^	7.26	128.1, CH	H^δ^	*n.d.*	*n.d.*
H^ζ^	7.36	126.3, CH			

n.d. = not determined; * = multiplicities were deduced from DEPT-HMQC.

#### 2.2.5. Solid Phase Peptide Synthesis as a Proof of Head-to-Tail Cyclization of (**1a**)

Finally we performed synthesis of head-to-tail-cyclized (**1a**) and head-to-side-chain-cyclized (**2**) (Supplementary Figure S3) by solid phase peptide synthesis with a hydrazide linker [12] to prove the correct cyclization topology in champacyclin (**1a**). Interestingly, the side chain derivative (**2**) shows significant lower ion abundance for the *m*/*z* 129 b_1_ ion compared to the synthetic compound (**1b**) and to the champacyclin (**1a**) (Supplementary Figure S3). This data independently supports a head-to-tail cyclization between N^α^_(*L*)_Lys^1^-CO-_(*AlloD*)_Ile^8^ in the natural product champacyclin (**1a**) rather than a head-to-side-chain between N^ζ^Lys^1^-CO-_(*AlloD*)_Ile^8^ (synthetic compound (**2**)). Very recently, Takada *et al.* (2013) [13] reported the isolation and structure elucidation of a series of cyclic octapeptides, termed surugamides A-E, from marine a *Streptomyces* sp. JAMM992 with similarities to champacyclin (**1a**) described in this article. Interestingly, surugamide A and champacyclin (**1a**) share the same amino acid residues but differ from each other in the stereochemistry of _(*D*)_Ile (surugamide A) and _(*AlloD*)_Ile in (**1a**) and the amino acid sequence.

## 3. Experimental Section

Unless otherwise noted, dry solvents were purchased from Fisher Scientific-ACROS (Schwerte, Germany). All other reagents were used as obtained (ABCR, Karlsruhe, Germany; Bachem, Bubendorf, Switzerland; Fisher Scientific, Schwerte, Germany; IRIS Biotech, Marktredwitz, Germany; Merck, Darmstadt, Germany; Sigma-Aldrich, Taufkirchen, Germany).

### 3.1. Sampling Sites

The *Streptomyces* strains were isolated from the following marine locations.

***Streptomyces*** **strain C42**. Samples of sediment cores were taken in an anoxic water layer (*ca.* 90 µmol/L H_2_S in the central Gotland Deep) at 241 m water depth at station AL 93 (coordinates: 57° 20.00′ N and 20° 03.00′ E). Samples were taken during the ALKOR cruise AL156 from 23 March to 2 April 2000, by using a Rumohr lot attached to a sediment corer. The samples were kept frozen on board at −20 °C until analysis [14].

***Streptomyces*** **strain i6a**. Sediment samples were taken in 3457 m water depth with a multiple corer at station #294 in the Urania Basin (coordinates: 35° 14.00′ N and 21° 28.60′ E) during the METEOR cruise M44/4 to the Eastern Mediterranean, in May 1999. The samples were kept frozen at −20 °C until analysis [15].

***Streptomyces*** **strain XX19**. Samples from the sediment surface in 5 m water depth were taken by scuba diving close to the shore line in Strande of Kiel Bight (Baltic Sea), in 2009 (coordinates: 54° 26.00′ N and 10° 10.00′ E). The sample was cooled in a freezer box until arriving in the lab on the same day and frozen at −20 °C until analysis.

### 3.2. Isolation Procedure

The sediment samples (1 g wet weight each) were dissolved in sterile-filtered (0.2 µm pore size) Baltic Sea water (5 mL) containing 1.5%–3% sodium chloride in dependence of sample origin (sodium chloride was additionally added to achieve 3%). The undiluted (100 µL) and the 10-fold diluted sample (100 µL) were transferred to agar plates and incubated at 28 °C for 3 month. The growth medium (GYM) contained 4 g yeast extract (BD-Bacto™, Becton Dickinson and Company, Sparks, MD, USA), 4 g d-(+)-glucose-monohydrate (VWR, Darmstadt, Germany), 4 g malt extract (BD-Bacto™) and 15 g agar (BD-Bacto™) in 1 L deionized water; after autoclaving the pH was 7.2. Single colonies were transferred to sterile agar plates and grown at 28 °C for 7–14 days. This procedure was repeated until pure cultures were obtained. Subsequently, purified colonies were phylogenetically identified by 16S rDNA gene sequencing.

### 3.3. Phylogenetic Classification

The phylogenetic classification of the strains C42, i6a, and XX19 was performed by sequencing of the 16S rRNA gene to determine the closest phylogenetic relative. For DNA analyses the DNA Kit PuReTaq™Ready-To-Go™ PCR Beads containing Taq-polymerase 0.5 U; 2.5 µL buffer and 0.5 µL dNTPs for each reaction were used. Amplification of the 16S rRNA gene (1500 bp) was performed with the primer pairs (concentration: each 10 pmol/µL): Eub27f with the sequence: 5′-ATT GGA TCC GTT TGA TCM TGG CTC AG-3′ (primer 1) (Noda *et al.*, 2006) [16]/Univ1492r with the sequence 5′-CGG TTA CCT TGT TAC GAC TT-3′ (primer 2) (Reysenbach *et al.*, 2000) [17]. The code “M” represents the bases C and A. From the total 25 µL PCR product 2.5 µL was transferred on a 1% agarose gel and separated at 200 mV for 20 min. The fragment length was about 1500 bp as determined with the DNA marker X (0.07–12.2 kbp). The sequencing procedure was performed according to Wiese *et al.* (2009) [18]. Storage of the PCR products was at −20 °C. The 16S rRNA sequences of the three isolates C42, i6a, and XX19 were edited using BioEdit (Hall, 1999) [19]. NCBI database (BLAST tool) and the Ribosomal Database Project (RDP). The Ribosomal Database Project database was used to select the type strains most closely related to our new isolates (Cole *et al.*, 2009) [20]. Sequence similarity values were determined with the “bl2seq” tool of the NCBI database (Tatsuova and Madden, 1999) [21]. The alignment of the 16S rRNA gene sequences (1375 bp) of the three isolates, of representatives of the *Streptomyces albidoflavus* group, of further type strains of the genus *Streptomyces*, and of *Micrococcus lylae* as outgroup was performed with Clustal X (Larkin *et al.*, 2007) [22] and refined manually. The phylogenetic tree was calculated with Molecular Evolutionary Genetics Analysis (MEGA5) by maximum likelihood analysis assuming the general time reversal (GTR) model and applying 500 bootstrap replicates (Tamura *et al.*, 2011) [23]. NJplot was used to display the phylogenetic tree (Perrière & Goug, 1996) [24].

### 3.4. PCR for Non-Ribosomal Peptide Synthetases (NRPS)

The amplification of the NRPS gene cluster was performed with the primers of the adenylation domain A2f (5′-AAG GCN GGC GSB GCS TAY STG CC-3′) and A3r (5′-TTG GGB IKB CCG GTS GIN CCS GAG GTG-3′) (Doekel and Marahiel, 2001) [25]. The codes “N”, “S”, “B”, and “Y” represent “unknown base”, G and C, “not A”, and pyrimidine C and T, respectively. The expected fragment length was about 300 bp of the PCR product, which was determined with the DNA Marker XIV (100 bp–1500 bp). From the total 25 µL PCR product, 5 µL was transferred on a 1.5% agarose gel and separated at 130 mV for 35 min. The permanent storage of the PCR products was −20 °C.

### 3.5. Optimization Experiments to Increase Champacyclin (**1a**) Synthesis

In order to increase the yield of champacyclin, growth conditions of strain C42 were optimized. In each experiment all basic parameters were maintained except the single variable parameter in the experiment: 100 mL culture volume, actinomycetes medium, except in the salt experiments, GYM medium was used, 1.5% salt (NaCl), 6 days incubation time, 28 °C incubation temperature, 125 rpm shaking and methanolic extraction.

Five different media were used for cultivation of the strain.

- TSB—Trypticase Soy Broth medium contained 3 g soybean casein digest broth (BD-BBL™ Trypticase™ Soy Broth, Dickinson and Company, Sparks, MD, USA), 10 g tropic marine salt in 1 L deionized water.
- OAK—Oak Flakes medium contained 20 g oak flakes and 3 mL trace element solution SL12 (3 g EDTA sodium salt, 42 mg ZnCl_2_, 50 mg MnCl_2_-tetrahydrate, 300 mg H_3_BO_3_, 190 mg CoCl_2_-hexahydrate, 1100 mg FeSO_4_ heptahydrate, 2 mg CuCl_2_-dihydrate, 24 mg NiCl_2_-hexahydrate, and 18 mg Na_2_Mo_4_-dihydrate in 1 L bidestilled water). The trace element solution was sterile filtered and added to the medium after autoclaving. The pH was 6.3 after autoclaving without adjustment.
- MIC—*Micromonospora* medium contained 10 g starch (VWR, Darmstadt, Germany), 5 g d-(+)-glucose-monohydrate (VWR), 4 g casein sodium salt from bovine milk (Sigma-Aldrich Chemie GmbH, Munich, Germany), 4 g yeast extract (BD-Bacto™, Dickinson and Company, Sparks, MD, USA), and 1 g calcium carbonate (Sigma-Aldrich Chemie GmbH) in 1 L bidestilled water. The pH was adjusted to 7.2 before autoclaving.
- ACT—Actinomycetes medium: contained 2 g casein sodium salt from bovine milk (Sigma-Aldrich Chemie GmbH, Munich, Germany), 4 g propionate acid sodium salt (Sigma-Aldrich Chemie GmbH), 0.1 g magnesium sulphate heptahydrate (VWR, Darmstadt, Germany), l-asparagine monohydrate (Roth, Karlsruhe, Germany), 0.5 g di-potassium hydrogenphosphate (VWR), 0.001 g ferric(III)-sulphate hydrate (Sigma-Aldrich Chemie GmbH), and 5 g glycerol (VWR) in 1 L sterile filtered Baltic Sea water (1.5% salt content). The pH was adjusted to 8.1 prior to autoclaving.
- GYM—GYM medium contained 4 g glucose, 4 g yeast extract, 4 g malt extract, 2 g CaCO_3_, 30 g tropic marine salt in 1 L bidestilled water. The pH of 7.2 was measured after autoclaving.

**Salt concentration**. Different salt contents in the medium were added to obtain the best growth conditions for the strain C42 to produce champacyclin. Experiments with 0%, 1%, 2%, 3%, and 4% NaCl, respectively, in the GYM medium were performed at an incubation temperature of 28 °C.

**Incubation time**. A time series was performed to determine the optimal growth time of strain C42 for champacyclin synthesis. The time series were performed for eight days with 100 mL culture aliquots taken for analysis from day four to eight as the first analysis in actinomycetes medium.

**Temperature**. The temperature optimum was determined in relation to the biomass at 20 °C, 24 °C, and 28 °C in actinomycetes medium.

**Influence of amino acids**. Addition of the amino acids of l-leucine, l-isoleucine, l-phenylalanine, and l-valine (each 1 mg/mL) in actinomycetes medium was performed to upgrade the champacyclin product.

**Cultivation, extraction, and isolation of champacyclin (1a).** The culture conditions that yielded both, the highest amounts of champacyclin and the lowest yield of heptaenes, were used for production and purification of the peptide as described as follows. Erlenmeyer flasks (2000 mL) with four baffles were filled with 1000 mL actinomycetes medium in 1 L bidestilled water. Sterile filtered (0.2 µm pore size, non-pyrogenic) l-leucine, l-isoleucine, and l-phenylalanine (1 mg/mL each) were added into the medium to increase the champacyclin production. For the extraction of champacyclin, the cells were separated from the medium by centrifugation (11,000× *g*, 20 min) and extracted with methanol. Cell lysis was performed with a cell disruption apparatus (Ultra Turrax, T25 basic, IKA^®^-Werke GmbH & Co. KG, Staufen, Germany) at 13,000 rpm for 30 s and shaken for 30 s to dissolve the oligopeptides. The methanol extract was evaporated in a rotary evaporator at 40 °C. The dry extract was dissolved in 2 mL methanol and filtered through a 0.2 µm PTFE filter and chromatographed on a reversed-phase HPLC with a C18 column (250 × 4.6 mm, 5 μm, 120 Å, ODS-AQ, YMC Europe, Dinslaken, Germany). A linear gradient of acidified (0.1% formic acid) of deionized H_2_O (A), and CH_3_CN (B) was used for separation (30% to 35% B in 10 min, 35% to 70% B in 15 min, 70% to 100% B in 2 min) at 40 °C and a flow rate of 1 mL/min. Champacyclin (**1a**) was eluted under these conditions at a retention time of 19 min on an Elite LaChrom HPLC (VWR, Hitachi, Darmstadt, Germany). The culture medium was extracted with ethyl acetate through phase separation and champacyclin (**1a**) was purified as described above.

**Physicochemical Properties of Champacyclin (1a).** UV/VIS (MeOH) λ_max_ (ε): 258 nm (1523); HR-ESI-(+)-Orbitrap-MS *m/z* 912.62574 [M + H]^+^ (relative mass error Δ_m_ = −2.572 ppm); ^1^H and ^13^C-NMR data from 2D NMR experiments in *d*_6_-DMSO, see Supplementary Table S5.

### 3.6. Determination of the Molar Absorption Coefficient

The molar absorption coefficient of champacyclin (**1a**) (50 μg in methanol) was determined by quantitative analysis of the compound constituent l-^12^C_9_^14^N-phenylalanine after acidic hydrolysis (6.0 M HCl at 110 °C for 20 h). The internal standard l-^13^C_9_^15^N-phenylalanine (1 μg/µL) (Isotec, Miamisburg, Ohio, OH, USA) was added before acidic hydrolysis. After derivatization of the dry hydrolyzate with *N*-methyl-*N*-(*tert*-butyldimethylsilyl)-trifluoroacetamide (MTBSTFA) in tetrahydrofuran (THF) and trifluoroacetic acid (TFA) (50/50/0.1; v/v/v), the solution was analyzed on a GC/EI-MS (Agilent Technology, Waldbronn, Germany). Analyses were conducted on a capillary column (30 m DB-1301, 0.32 mm i.d., 0.25 μm film thickness) under the following separation conditions: 1 min at 120 °C, 120 to 250 °C at a rate of 10 °C/min. The integrals of the fragment ions at *m/z* 234 (30%), *m/z* 308 (15%), *m/z* 336 (11%), and *m/z* 243, *m/z* 317, and *m/z* 346 of l-^12^C_9_^14^N-phenylalanine and l-^13^C_9_^15^N phenylalanine, respectively, were applied to determine the amount of non-labeled amino acids. This value was correlated to the UV absorption of champacyclin at 258 nm in methanol measured before acidic hydrolysis.

### 3.7. Antimicrobial Activity

Antimicrobial bioassays were performed against Gram-positive and Gram-negative bacteria and yeast using the following test organisms: *Bacillus subtilis* (DSM 347), *Escherichia coli* K-12 (DSM 498), *Staphylococcus lentus* (DSM 6672), *Pseudomonas syringae* pv*. aptata* (DSM 50252), *Pseudomonas fluorescens* (NCIMB 10586), *Xanthomonas campestris* (DSM 2405), *Ralstonia solanacearum* (DSM 9544), *Candida glabrata* (DSM 6425), *and Erwinia amylovora* (DSM 50901). Assay mixtures were prepared by transferring 10 µL aliquots of methanolic solutions of extracts or pure substances into three wells of a 96-well microtiter plate and evaporating the solvent in a vacuum centrifuge. Overnight cultures of the test organisms in TSB medium (BD-BBL™ Trypticase™ Soy Broth (Dickinson and Company, Sparks, MD, USA) 12 g/L; NaCl, 5 g/L) were diluted to an optical density at 600 nm (OD_600_) of 0.001, and 200 µL aliquots of the resulting suspensions were added to the wells. After incubation for 15 h at 600 rpm and 28 °C, 10 µL of a resazurin solution (0.2 mg/mL phosphate-buffered saline, pH 7.2) was added to each well and the plate was incubated at 28 °C until the color of the negative control changed from blue to pink. For evaluation of the cell viability, the transformation of resazurin to resorufin was assayed by measuring the fluorescence at 560 nm after excitation at 590 nm. The resulting values were compared to those for a positive control (100 µg/well chloramphenicol for the bacteria, 200 µg/well cycloheximide for yeast) and a negative control (no compound) on the same plate.

### 3.8. Mass Spectrometry (MS)

HPLC-HR-ESI-(+)-Orbitrap-(SID)-MS*^n^* was performed on an Orbitrap LTQ XL mass spectrometer (Thermo Fisher Scientific, Waltham, MA, USA) coupled to an Agilent 1260 HPLC-system (Santa Clara, CA, USA). In Source Fragmentation (SID) and subsequent MS*^n^*-analyses were performed using direct infusion with a concentration 0.1 mg/mL The flow was adjusted between 1 and 20 µL/min to obtain high abundance. Further parameters for the analyses are described in the supporting material.

Low-Resolution-MS data of the partial hydrolyzate (**1a**) were performed on an ESI-Triple-Quadrupol-MS 6460 (Agilent Technologies, Waldbronn, Germany) coupled to an Agilent 1290 UHPLC-system (Santa Clara, CA, USA), with the parameters described in the Supplementary Information.

### 3.9. Nuclear Magnetic Resonance (NMR) Spectroscopy

^1^H-NMR and 2D-NMR experiments data were acquired on a 600 MHz NMR spectrometer (Bruker, Karlsruhe, Germany) in *d*_6_-DMSO (500 μL), equipped with a TXI probe head with Z-Gradient. All chemical shifts were calibrated on the residual solvent peak (*d*_6_-DMSO, 2.50 ppm (^1^H) and 39.5 ppm (^13^C)). The chemical shifts, reported in delta (δ) units, in parts per million (ppm) are referenced relatively to TMS. Additional spectra and parameter are described in NMR section in the Supplementary Information.

### 3.10. Hydrolysis of (**1a**)

#### 3.10.1. Partial Hydrolysis

Partial hydrolysis of (**1a**) (0.1 mg) was performed at 110 °C in aqueous 6 M hydrochloric acid solution for amino acid analysis (200 µL) (Sigma Aldrich, Schnelldorf, Germany) for 7 h in glass-ampoules. After 7 h, the sample was freeze-dried prior derivatization for GC/MS analysis. The status of the reaction was measured by mass spectrometry.

#### 3.10.2. Total Hydrolysis

Total hydrolyses of (**1a**) (0.1 mg) were performed at 110 °C in aqueous 6 M hydrochloric acid solution (200 µL) for amino acid analysis (Sigma Aldrich, Schnelldorf, Germany) under vacuum for 24 h in glass-ampoules. After 24 h, the hydrochloric acid was removed in a gentle stream of nitrogen.

### 3.11. Chiral GC/EI-PCI-MS Analysis

GC/EI-PCI-MS analyses were performed on a GC/MS 5975C (Agilent Technologies, Waldbronn, Germany). Aliquots of derivatized amino acids/dipeptide of 0.2 to 5 µL were injected to the GC/MS 5975C (Agilent Technologies, Waldbronn, Germany), equipped with a Chirasil^®^-(l)-Val column. Parameters and conditions are described in supporting information. Further EI- and PCI-MS spectra of all dipeptides analyzed of partial hydrolyzate and synthetic dipeptides are also shown in the Supplementary Information.

#### 3.11.1. Amino Acid Analysis as *N*-Pentafluoropropionic 2-Propyl Amino Acid Derivatives

Amino acid standards (<0.1 mg/mL) and dried total hydrolyzate of (**1a**) were suspended in a total volume of 200 µL acetylchloride:2-propanol (v:v, 150:50 µL) in Reacti-Vials sealed and derivatized for 30 min at 110 °C. Excess of solvents was removed in a gentle steam of nitrogen. To the dry residues 50 µL pentafluoropropionic anhydride (PFPA) and 100 µL dichloromethane were added and derivatized for 15 min at 110 °C. Excess of reagents was evaporated in a stream of nitrogen and dissolved in 100 µL DCM.

#### 3.11.2. Dipeptide Analysis as *N*-Trifluoroacetyl Methyl Dipeptide Derivatives

Partial hydrolyzed (**1a**) and synthetic dipeptides were dried before derivatization. The protocols were mainly adapted by Pätzold *et al.* (2006) [7].

To the samples a total volume of 360 µL acetyl chloride:methanol (v:v, 60:300 µL) were added in Reacti-Vials sealed and derivatized for 3 h at room temperature (21 °C). The excess of reaction mixture was evaporated in a gentle steam of nitrogen. To the dry residues 50 µL trifluoroacetic anhydride (TFAA) and 100 µL dichloromethane (DCM) were added and derivatized for 10 min at room temperature (21 °C) and then 5 min at 110 °C. The samples were cooled down to room temperature (21 °C) and again dried in a steam of nitrogen and finally dissolved in 100 µL DCM.

### 3.12. Solid Phase Peptide Synthesis (SPPS)

Standard Fmoc-protected amino acids were purchased from Orpegen (Heidelberg, Germany). Fmoc-_(*AlloD*)_Ile-OH and Boc-_(*L*)_Lys(Fmoc)-OH were bought from Iris Biotech (Marktredwitz, Germany). The coupling reagents were obtained from Iris Biotech (Marktredwitz, Germany). The TCP resin was bought from Intavis (Reutlingen, Germany) and the 4-Fmoc-hydrazinobenzoyl AM Novagel resin for cyclizing cleavage was bought from Merck (Darmstadt, Germany). DMF (99.8%) was purchased from VWR (Darmstadt, Germany). The chemicals for peptide cleavage were obtained from Sigma-Aldrich (Schnelldorf, Germany) and ABCR (Karlsruhe, Germany). Automated solid phase Fmoc-synthesis was performed on a Prelude parallel synthesizer by Protein Technologies (Tucson, AZ, USA).

#### 3.12.1. Synthesis of Dipeptides

**Synthesis protocol for automated solid phase peptide synthesis**: Automated solid-phase peptide synthesis was performed in 50 µmol scale. Loading: To a 10 mL syringe reactor with frit and cap were added 1 g of tritylchloride (TCP) resin (1.56 mmol/g) and 7 mL dry DCM. The resin was pre-swollen for 10 min and the solvent was removed by evaporation in vacuum. A mixture of the amino acid (0.6 mmol) and 3 equivalents of DIPEA dissolved in 5 mL dry DCM was added to the resin. The syringe was agitated for 30 min at room temperature. The solution was removed and the resin was washed (2 × 5 mL DMF, 2 × 5 mL DCM). Capping of non-reacted functional groups of the resin was performed with DCM, methanol and DIPEA 80:15:5 (2 × 10 mL, 10 min). After washing (5 × 5 mL DMF), Fmoc-removal was achieved with DMF/piperidine (4:1, 5 mL, 1 × 2 min, 1 × 20 min). After final washing (2 × 5 mL DMF, 1 × 5 mL methanol, 3 × 5 mL DCM), the resin was dried *in vacuo*.

**Coupling of Fmoc/tBu-protected amino acids**: To 100 mg of the resin (~0.5 mmol/g), a 0.15 M solution of the amino acid in DMF (3 eq relative to resin loading) was added. After addition of a 0.3 M solution of DIPEA in DMF (3 eq) and a 0.15 M solution of HATU in DMF (3 eq), the reaction solution was mixed for 60 min. A second coupling was performed for 60 min. Finally, the resin was washed with DMF (6 × 2.5 mL).

**Fmoc removal**: DMF/piperidine (4:1, 2.5 mL) was added to the resin and mixed for 2.5 min. The procedure was repeated 4 times. The resin was washed with DMF (5 × 2.5 mL), then DCM (5 × 2 mL).

**Global deprotection**: The resin was transferred to a 5 mL syringe with frit and cap. After addition of the cleavage cocktail (TFA, H_2_O 90:10), the syringe was shaken for 2 h. The cleaving solution was collected and the resin was washed with MeOH (2 × 3 mL). The combined fractions were concentrated *in vacuo*.

#### 3.12.2. Synthesis of Head-to-Tail **(1b)** between N^α^_(L)_Lys^1^-CO-_(AlloD)_Ile^8^ and Head-to-Side-Chain (**2**) between N^ζ^-_(L)_Lys-CO-_(AlloD)_Ile^8^ Cyclized Peptides for Mass Spectrometry

**Synthesis protocol for automated solid-phase peptide synthesis**: Automated solid-phase peptide synthesis was performed in 64 µmol scale. Loading: 100 mg of the hydrazine resin was deprotected (DMF/piperidine 4:1, 2.5 mL, 3 × 3 min), and then washed with DMF (6 × 2 mL). The first amino acid was loaded with standard coupling procedure.

**Coupling of Fmoc/tBu-protected amino acids**: To 100 mg of the resin (0.64 mmol/g), a 0.25 M solution of the amino acid in DMF (4 eq relative to resin loading) was added. After addition of a 0.5 M solution of DIPEA in DMF (4 eq) and a 0.25 M solution of TBTU in DMF (4 eq), the reaction solution was mixed for 30 min. A second coupling was performed for 30 min. Finally the resin was washed with DMF (6 × 2.5 mL).

**Fmoc removal**: DMF/piperidine (4:1, 2.5 mL) was added to the resin and mixed for 2.5 min. The procedure was repeated 4 times. The resin was washed with DMF (6 × 2.5 mL). After the final coupling cycle, the resin was washed with DCM (5 × 2 mL).

**Cyclising cleavage**: The resin was transferred to a 5 mL syringe with frit and cap. A mixture of 2,6-*O*-dichlorobenzene and pyridine (1:1, 3 mL) was saturated with Cu(OAc)_2_ and added to the resin. The syringe was shaken for 3 days. The solution was poured into 1 M aqueous HCl (5 mL) and the resin was washed with DCM (3 × 3 mL). The layers were separated and the aqueous layer was extracted with DCM (3 × 5 mL). The combined organic layers were concentrated *in vacuo*.

## 4. Conclusions

The fermentation, isolation and structure elucidation of the marine derived cyclooctapeptide champacyclin (**1a**) was described in this contribution. According to NMR spectroscopy measurements, partial and total hydrolysis followed by derivatization and GC-MS analytics as well as ESI-IonTrap-MS*^n^* experiments, the sequence and the absolute stereochemistry of the Xle rich cyclic peptide could be elucidated as [_(*L*)_Lys^1^-_(*L*)_Ile^2^-_(*L*)_Ile^3^-_(*D*)_Phe^4^-_(*D*)_Leu^5^-_(*L*)_Ile^6^-_(*D*)_Ala^7^-*_(AlloD)_*Ile^8^] (**1a**) with a suggested head-tail cyclization (N^α^_(*L*)_Lys^1^-CO-_(*AlloD*)_Ile^8^). The use of different analytical methods including synthesis of dipeptides was necessary to unambiguously assign the sequence and configuration of the peptide (**1a**). While the linear sequence could be partially elucidated by the 2D-NMR data, the interpretation of MS*^n^*-experiments was a challenge due to the high number of Leu and Ile residues. Only the synthesis of dipeptides combined with the use of GC-PCI/EI-MS and total/partial hydrolysis studies finally validated the NMR results and further enabled the stereospecific assignment within the sequence.

## Supplementary Files

Supplementary File 1 Supplementary Information (PDF, 3103 KB)
