# Supplementary material for: Champacyclin, a New Cyclic Octapeptide from Streptomyces Strain C42 Isolated from the Baltic Sea
Source: Mar Drugs. 2013 Dec 2;11(12):4834–57. doi: 10.3390/md11124834 (PMC3877890; doi:10.3390/md11124834)

Supplementary Information

Table S1. Parameter for HPLC-MS^n analysis of champacyclin (**1a**) and synthetic reference peptides (**1b**, **2**).
Table S2. Parameter for NMR analytics of the natural product champacyclin (**1a**).
Table S3. NMR parameters for ^{1}H- and 2D-NMR experiments for (**1a**) measurements on a DRX 600 (Bruker, Karlsruhe, Germany) NMR spectrometer at the FMP Berlin.
Table S4. Parameter for HPLC-MS analysis of Partial hydrolysis of the natural product champacyclin (**1a**).
Table S5. Parameters for GC/MS analysis of amino acids and dipeptides.
Table S6. Peak areas and relative Quantification of the *N*-pentafluoropropionic 2-propyl ester derivatives of the total hydrolysate of (**1a**) as shown in Figure S13.
Figure S1. LC-MS analysis. (**A**) Total ion chromatogram (TIC). (**B**) Extracted ion chromatogram (EIC) of (**1a**) (*m/z* 912-914) (**C**) Mass spectrum for the EIC as shown in (**B**).
Figure S2. In-source-fragmentation-MS^n-experiments for (**1a**). (**A**) SID for (**1a**) with 70 eV. MS^2 for the 185 Da isobaric ion fragment y_2/b_7y_3 with the ESI-(+)-Ion-Trap (CE 15 eV). (**B**) MS^3 on 86 Da ion with ESI-(+)-Ion-Trap (CE 20 eV) for the isobaric ions y_2/b_7y_3. Formation of 69 Da Ion is indicative for an Ile-residue. (**C**) Possible mechanism for the subsequent MS^n-fragmentation series suggesting at least one Ile at position 6 and/or 8.
Figure S3. HR-ESI-(+)-SID-Orbitrap-MS of (**A**) natural product (**1a**) with SID at 65eV (**B**) Head-to-tail cyclization N^{α}-$_{(L)}$Lys^1-CO-$_{(AlloD)}$Ile^8 in (**1b**) (**C**) Head-to-side-chain cyclization N^{ζ}-$_{(L)}$Lys-CO-$_{(AlloD)}$Ile^8 in (**2**). Note: the relative abundance for the b_1 Ion *m/z* 129 $[M + H]^+$ is dramatically decreased in (**C**). Thus is indicative that the natural product in A (**1a**) is head-to-tail cyclized.
Figure S4. ^{1}H-NMR of (1a) in d_6-DMSO at 600 MHz.
Figure S5. ^{1}H-(PRESAT)-NMR of (**1a**) in d_6-DMSO at 600 MHz. PRESAT was used for water suppression.
Figure S6. ^{1}H-^{13}C-HMQC of (**1a**) in d_6-DMSO at 600 MHz.
Figure S7. HMQC-TOCSY (spin lock 80 ms) of (**1a**) in d_6-DMSO at 600 MHz.
Figure S8. ^{1}H-^{13}C-HMBC of (**1a**) in d_6-DMSO at 600 MHz.
Figure S9. ^{1}H-^{1}H-COSY of (**1a**) in d_6-DMSO at 600 MHz.
Figure S10. ^{1}H-^{1}H-NOESY (mixing time 100 ms) of (**1a**) in d_6-DMSO at 600 MHz.
Figure S11. ^{1}H-^{1}H-TOCSY (spin lock 60 ms) data of (**1a**) in d_6-DMSO at 600 MHz.
Figure S12. HPLC-ESI-(+)-MS analysis of partial hydrolysate of (**1a**) after 7 h at 110 ℃ with 6 M HCl. (**A**) EICs for dipeptides, (**B**) EICs for tripeptides (**C**) EIC for tetrapeptides. Note: Absolute stereochemistry and constitution of dipeptides was assigned by GC/MS analysis. This analysis shows the status of the hydrolysis reaction, required for subsequent derivatization and analytics. Formation of lysine-dipeptides could not be observed.
Figure S13. Chiral GC-MS analysis of total hydrolysate of (**1a**) on a Chirasil®-(L)-Val column. Amino acids were analyzed as their *N*-pentafluoropropionic 2-propyl esters and identified by their $[M + H]^+$-Ions and their fragmentation products using positive chemical ionization (PCI) with methane as reagent gas.
Figure S14. Chiral GC-PCI-MS analysis on a Chirasil®-(L)-Val column of Xle amino acid standards as *N*-pentafluoropropionic 2-propyl esters and identified by their $[M + H]^+$-Ions using positive chemical ionization (PCI) with methane as reagent gas.

Figure S15. PCI-MS spectra of *N*-trifluoroacetyl-$_{(L)}$Ile-$_{(D)}$Ala-methyl ester found in partial hydrolysate of (**1a**) at R_t = 19.97 min on a Chirasil®-(L)-Val column.
Figure S16. EI-MS spectra of *N*-trifluoroacetyl-$_{(L)}$Ile-$_{(D)}$Ala-methyl ester found in partial hydrolysate of (**1a**) at R_t = 19.97 min on a Chirasil®-(L)-Val column.
Figure S17. PCI-MS spectra of *N*-trifluoroacetyl-$_{(L)}$Ile-$_{(L)}$Ile-methyl ester found in partial hydrolysate of (**1a**) at R_t = 22.5 min on a Chirasil®-(L)-Val column.
Figure S18. EI-MS spectra of *N*-trifluoroacetyl-$_{(L)}$Ile-$_{(L)}$Ile-methyl ester found in partial hydrolysate of (**1a**) at R_t = 22.5 min on a Chirasil®-(L)-Val column.
Figure S19. PCI-MS spectra of *N*-trifluoroacetyl-$_{(D)}$Leu-$_{(L)}$Ile-methyl ester found in partial hydrolysate of (**1a**) at R_t = 24.6 min on a Chirasil®-(L)-Val column.
Figure S20. EI-MS spectra of *N*-trifluoroacetyl-$_{(D)}$Leu-$_{(L)}$Ile-methyl ester found in partial hydrolysate of (**1a**) at R_t = 24.6 min on a Chirasil®-(L)-Val column.
Figure S21. PCI-MS spectra of *N*-trifluoroacetyl-$_{(L)}$Ile-$_{(D)}$Phe-methyl ester found in partial hydrolysate of (**1a**) at R_t = 46.68 min on a Chirasil®-(L)-Val column.
Figure S22. EI-MS spectra of *N*-trifluoroacetyl-$_{(L)}$Ile-$_{(D)}$Phe-methyl ester found in partial hydrolysate of (**1a**) at R_t = 46.68 min on a Chirasil®-(L)-Val column.
Figure S23. PCI-MS spectra of synthetic *N*-trifluoroacetyl-Leu-Leu-methyl ester.
Figure S24. EI-MS spectra of synthetic *N*-trifluoroacetyl-Leu-Leu-methyl ester.
Figure S25. PCI-MS spectra of synthetic *N*-trifluoroacetyl-Ile-Ile-methyl ester.
Figure S26. EI-MS spectra of synthetic *N*-trifluoroacetyl-Ile-Ile-methyl ester.
Figure S27. PCI-MS spectra of synthetic *N*-trifluoroacetyl-Leu-Ile-methyl ester.
Figure S28. EI-MS spectra of synthetic *N*-trifluoroacetyl-Leu-Ile-methyl ester.
Figure S29. PCI-MS spectra of synthetic *N*-trifluoroacetyl-Ile-Leu-methyl ester.
Figure S30. EI-MS spectra of synthetic *N*-trifluoroacetyl-Ile-Leu-methyl ester.
Figure S31. PCI-MS spectra of synthetic *N*-trifluoroacetyl-Ala-Leu-methyl ester.
Figure S32. EI-MS spectra of synthetic *N*-trifluoroacetyl-Ala-Leu-methyl ester.
Figure S33. PCI-MS spectra of synthetic *N*-trifluoroacetyl-Leu-Ala-methyl ester.
Figure S34. EI-MS spectra of synthetic *N*-trifluoroacetyl-Leu-Ala-methyl ester.
Figure S35. PCI-MS spectra of synthetic *N*-trifluoroacetyl-Ala-Ile-methyl ester.
Figure S36. EI-MS spectra of synthetic *N*-trifluoroacetyl-Ala-Ile-methyl ester.
Figure S37. PCI-MS spectra of synthetic *N*-trifluoroacetyl-Ile-Ala-methyl ester.
Figure S38. EI-MS spectra of synthetic *N*-trifluoroacetyl-Ile-Ala-methyl ester.
Figure S39. PCI-MS spectra of synthetic *N*-trifluoroacetyl-Leu-Phe-methyl ester.
Figure S40. EI-MS spectra of synthetic *N*-trifluoroacetyl-Leu-Phe-methyl ester.
Figure S41. PCI-MS spectra of synthetic *N*-trifluoroacetyl-Phe-Leu-methyl ester.
Figure S42. EI-MS spectra of synthetic *N*-trifluoroacetyl-Phe-Leu-methyl ester.
Figure S43. PCI-MS spectra of synthetic *N*-trifluoroacetyl-Ile-Phe-methyl ester.
Figure S44. EI-MS spectra of synthetic *N*-trifluoroacetyl-Ile-Phe-methyl ester.
Figure S45. PCI-MS spectra of synthetic *N*-trifluoroacetyl-Phe-Ile-methyl ester.
Figure S46. EI-MS spectra of synthetic *N*-trifluoroacetyl-Phe-Ile-methyl ester.

Table S1. Parameter for HPLC-MS^n analysis of champacyclin (**1a**) and synthetic reference peptides (**1b**, **2**).

Parameter for HPLC-MSn Analysis		
**Mass Spectrometer**	LTQ Orbitrap XL Mass Spectrometer (Thermo Fisher Scientific, Waltham, MA, USA)	
**HPLC**	1260 HPLC-system (Agilent, Santa Clara, CA, USA)	
**Column**	Thermo, Waltham, MA, USA, Hypersil-Gold, 5 µm, 50 × 2.1 mm	
**Solvent System**	A: H_2O + 0.1% HCOOH	
	B: ACN + 0.1% HCOOH	
**Flow Rate**	0.25 mL/min	
**Gradient**	**B%**	**Time**
	5% B	0 min
	5% B	1 min
	100% B	6 min
	100% B	10 min
	5% B	10.10 min
	5% B	13 min

Table S2. Parameter for NMR analytics of the natural product champacyclin (**1a**).

Parameter for the NMR Analytics	
NMR-Spectrometer	DRX 600 MHz (^{1}H) (Bruker, Karlsruhe, Germany)
Solvent	d_6-DMSO, 500 µL
Probe Head	TXI 5 mm, with Z-Gradient

Table S3. NMR parameters for ^{1}H- and 2D-NMR experiments for (**1a**) measurements on a DRX 600 (Bruker, Karlsruhe, Germany) NMR spectrometer at the FMP Berlin.

Experiment	Pulse Program	Scans	TD (F1/F2)	Additional Information
^{1}H-NMR	zg30	64	65536	/
^{1}H-NMR-PRESAT	MF1hpresat	32	8192	/
^{1}H-^{1}H-COSY	MFdqfcosypre	8	4096/1024	/
^{1}H-^{1}H-NOESY	MFnoesypre	32	4096/1024	100 ms
^{1}H-^{1}H-TOCSY	Psdipsi2pre	8	4096/1024	60 ms
^{1}H-^{13}C-HMQC	MFbihmqceaf2	128	1024/512	/
^{1}H-^{13}C-HMBC	MFhmbceaf2	80	4096/1024	/
HSQC-TOCSY	MFbihmqctocf2	8	1024/512	80 ms

Table S4. Parameter for HPLC-MS analysis of Partial hydrolysis of the natural product champacyclin (**1a**).

**Parameter for HPLC-MS Analysis**		
**Mass Spectrometer**	QQQ-MS-6460 (Agilent Technologies, Waldbronn Germany)	
**HPLC**	Agilent 1290 UHPLC-system (Agilent Technologies, Waldbronn, Germany)	
**Column**	Agilent, Waldbronn Germany, Eclipse Plus C18 RRHD column, 1.8 µm, 2.1 × 50 mm	
**Solvent System**	A: H_2O + 0.1% HCOOH	
	B: ACN + 0.1% HCOOH	
**Flow Rate**	0.3 mL/min	
**Gradient**	**B%**	**Time**
	5% B	0 min
	5% B	1 min
	20% B	20 min
	70% B	6 min
	100% B	6.5 min
	100% B	8 min
	5% B	8.5 min
	5% B	10 min

Table S5. Parameters for GC/MS analysis of amino acids and dipeptides.

**Parameters for GC/MS Analysis**	
GC Column	Chirasil®-(L)-Val (Agilent CP7495, Waldbronn Germany), 200 °C, 25 m, 250 µm × 0.12 µm
Temperature Program *N-trifluoroacetyl methyl ester:dipeptides*	140 °C (10 min isothermal), 190 °C, 5 °C/min (33min isothermal), 195 °C, 5 °C/min (20 min isothermal)
Temperature Program *N-pentafluoropropionic 2-propyl ester:amino acids*	70 °C (5 min isothermal), 100 °C, 2 °C/min, 190 °C, 3.5 °C/min (10 min isothermal)
Gas Chromatograph Mass Spectrometer (GC-MS)	GC/MS 5975C (Agilent Technologies, Waldbronn, Germany)
Scan	Full-Scan, 50–800 *m/z*
Heater/MSD-Transfer Line	300 °C/280 °C
Flow	1.2 mL/min, 40.3 cm/s with He as carrier gas
PCI-Mode	Energy: 105.2 eV, Emission: 182 µA, MS-Source: 300 °C, MS Quad: 150 °C Collision gas: Methane with flow rate 19%
EI-Mode using CI-Source	Energy: 105.2 eV, Emission: 250 µA, MS-Source: 300 °C, MS Quad: 150 °C Collision gas: Methane with flow rate 0%
Single Ion Monitoring (SIM) for Higher Sensitivity	SIM was used with ions represented in all *N-trifluoroacetyl methyl ester dipeptide derivatives* #1: *m/z* 389 $[M + H]^+$ (Phe-Xle/Xle-Phe) #2: *m/z* 313 $[M + H]^+$ (Ala-Xle/Xle-Ala) #3: *m/z* 355 $[M + H]^+$ (Xle-Xle)

Table S6. Peak areas and relative Quantification of the *N*-pentafluoropropionic 2-propyl ester derivatives of the total hydrolysate of (**1a**) as shown in Figure S13.

Amino Acids (*N*-Pentafluoropropionic 2-Propyl Ester Derivatives)	Peak Areas (Absolute) RTE Integrator	Ratio/Racemization Factor (%)
$_{(D)}$Ala:$_{(L)}$Ala	64,586,729:*n.d.*	*n.d.*
$_{(AlloD)}$Ile:$_{(L)}$Ile	40,548,258:114,743,611	1:2.8
$_{(D)}$Leu:$_{(L)}$Leu	92,808,033:5,641,417	93.9%:6.1%
$_{(D)}$Phe:$_{(L)}$Phe	97,483,637:3,178,567	96.8%:3.2%
$_{(D)}$Lys:$_{(L)}$Lys	3,493,779:69,688,561	95%:5.0%

n.d. = not determined.

Figure S1. LC-MS analysis. (**A**) Total ion chromatogram (TIC). (**B**) Extracted ion chromatogram (EIC) of (**1a**) (*m/z* 912-914) (**C**) Mass spectrum for the EIC as shown in (**B**).

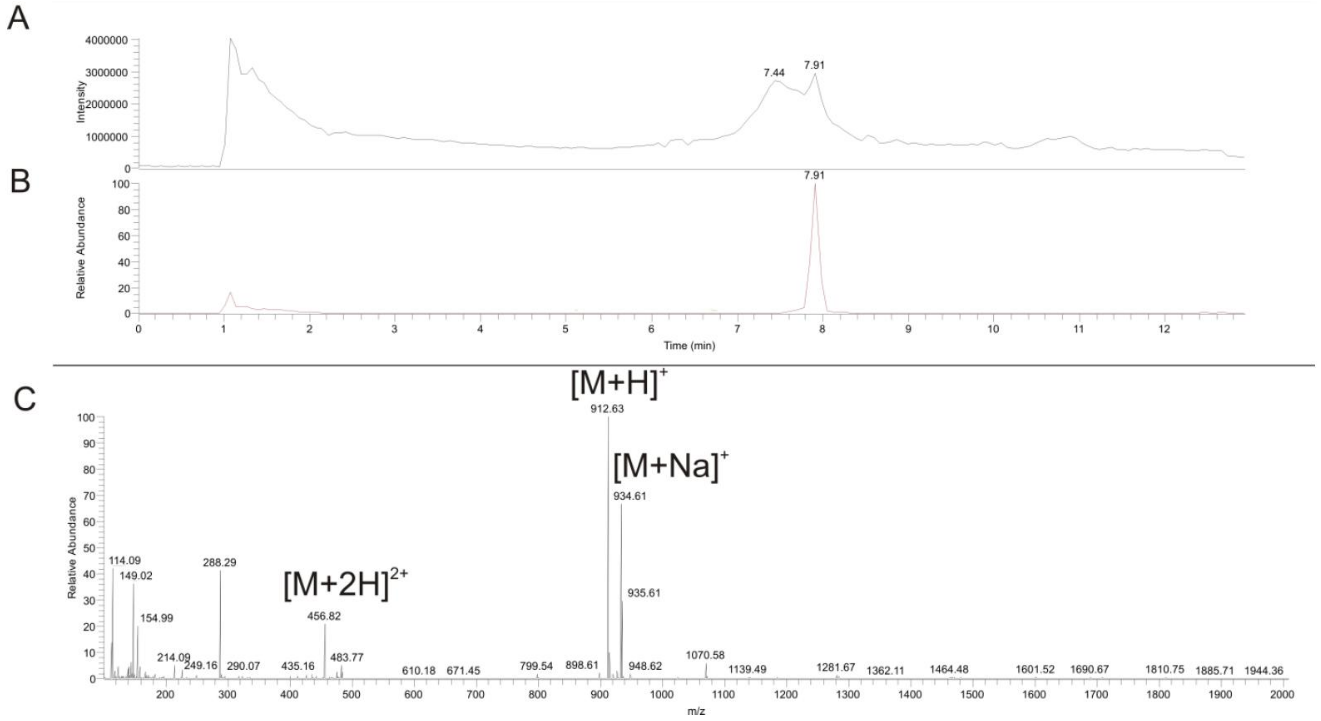

Figure S2. In-source-fragmentation-MS^n-experiments for (**1a**). (**A**) SID for (**1a**) with 70 eV. MS^2 for the 185 Da isobaric ion fragment y_2/b_7y_3 with the ESI-(+)-Ion-Trap (CE 15 eV). (**B**) MS^3 on 86 Da ion with ESI-(+)-Ion-Trap (CE 20 eV) for the isobaric ions y_2/b_7y_3. Formation of 69 Da Ion is indicative for an Ile-residue. (**C**) Possible mechanism for the subsequent MS^n-fragmentation series suggesting at least one Ile at position 6 and/or 8.

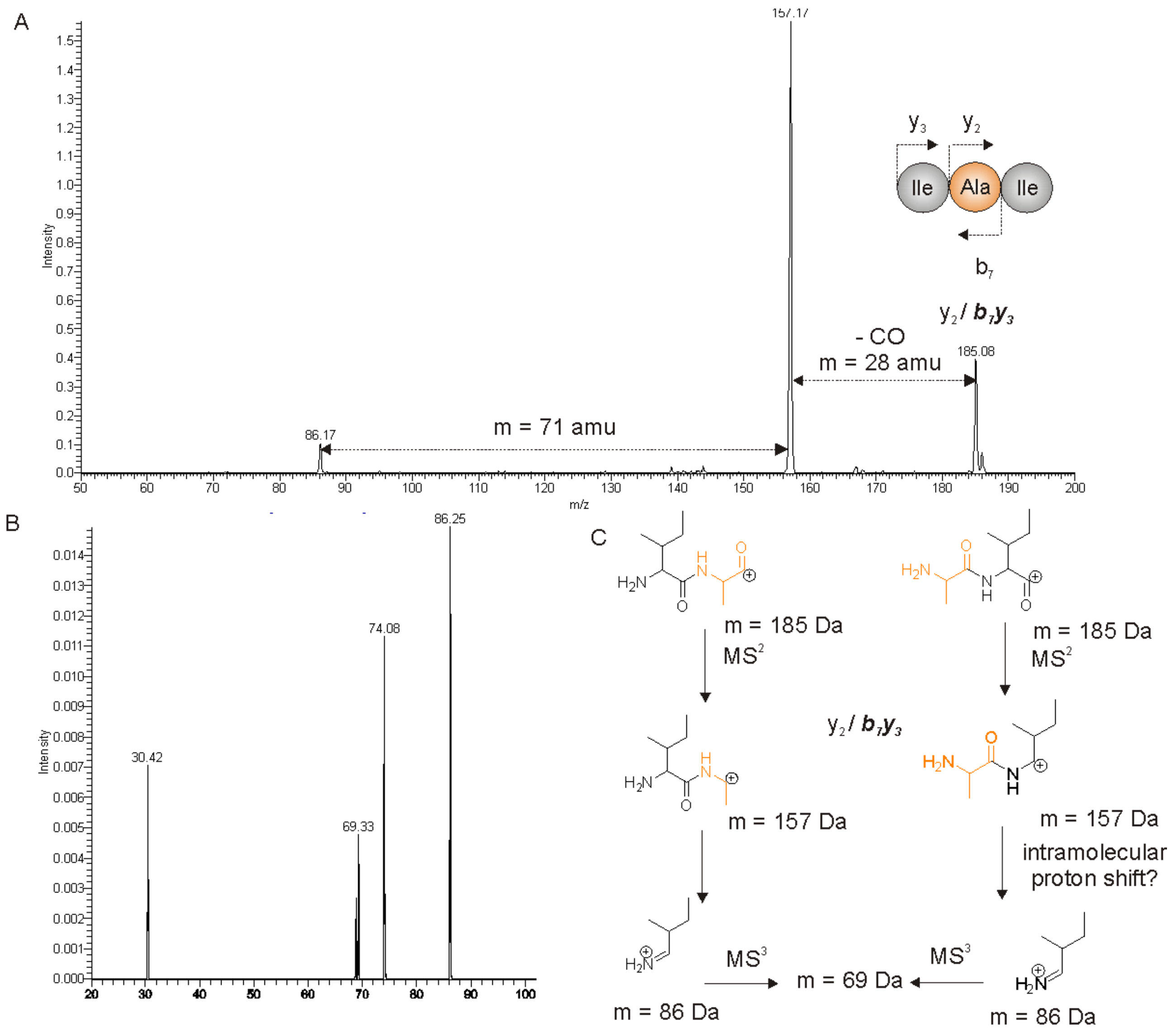

Figure S3. HR-ESI-(+)-SID-Orbitrap-MS of (**A**) natural product (**1a**) with SID at 65eV (**B**) Head-to-tail cyclization $N^{\alpha}{}_{(L)}$Lys^{1}-CO-$_{(AlloD)}$Ile^{8} in (**1b**) (**C**) Head-to-side-chain cyclization N^{ζ}-$_{(L)}$Lys-CO-$_{(AlloD)}$Ile^{8} in (**2**). Note: the relative abundance for the b_1 Ion *m*/*z* 129 $[M + H]^+$ is dramatically decreased in (**C**). Thus is indicative that the natural product in A (**1a**) is head-to-tail cyclized.

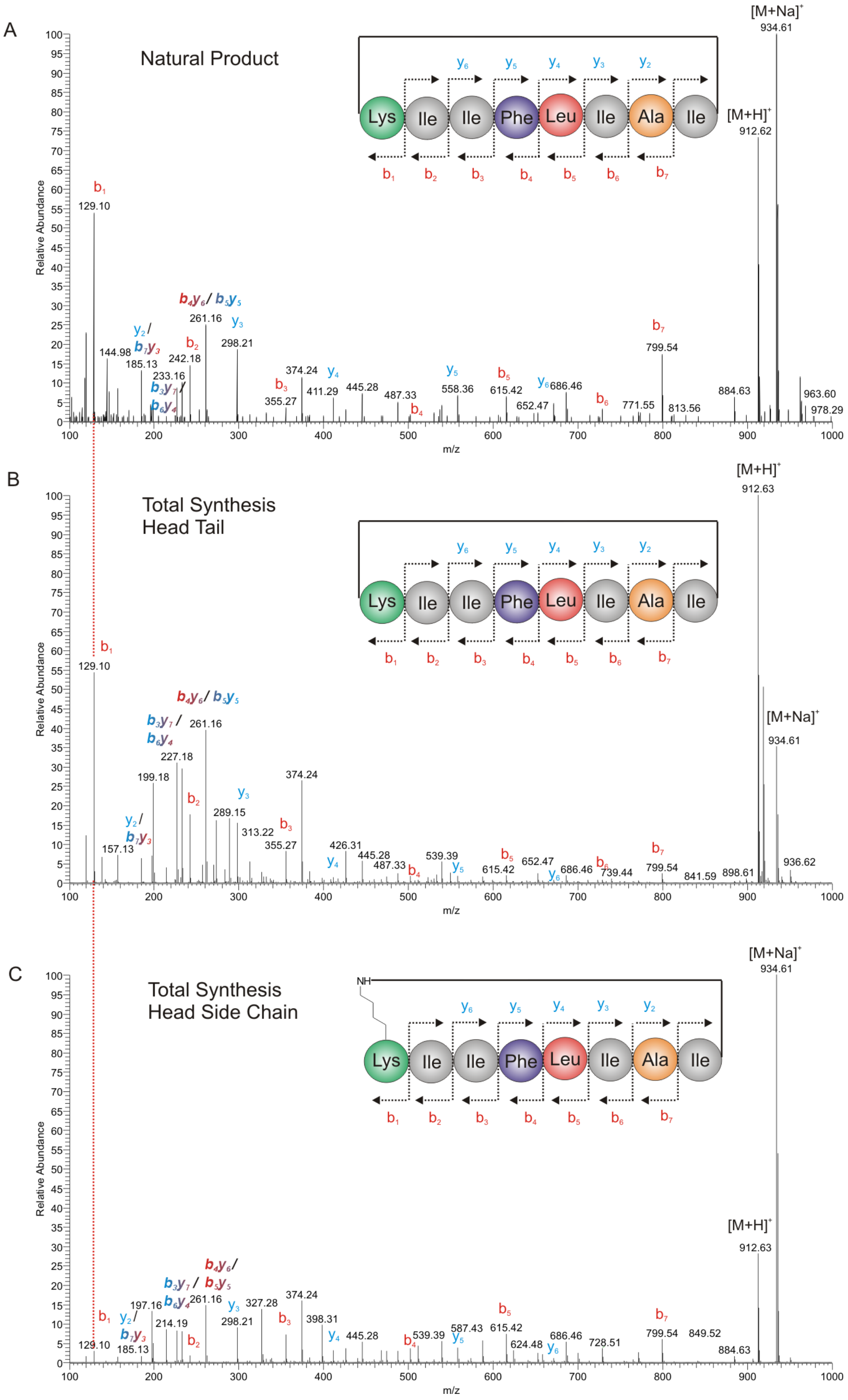

Figure S4. ^{1}H-NMR of (**1a**) in d_6-DMSO at 600 MHz.

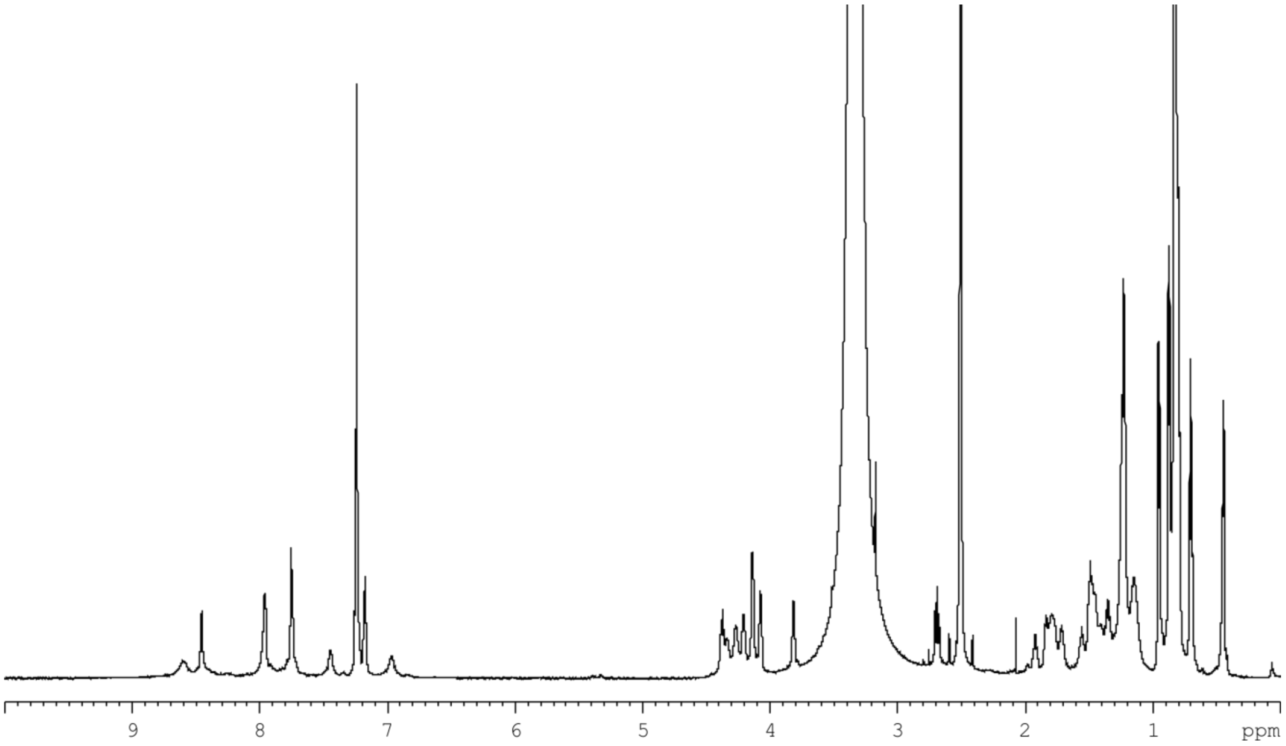

Figure S5. ^{1}H-(PRESAT)-NMR of (**1a**) in d_6-DMSO at 600 MHz. PRESAT was used for water suppression.

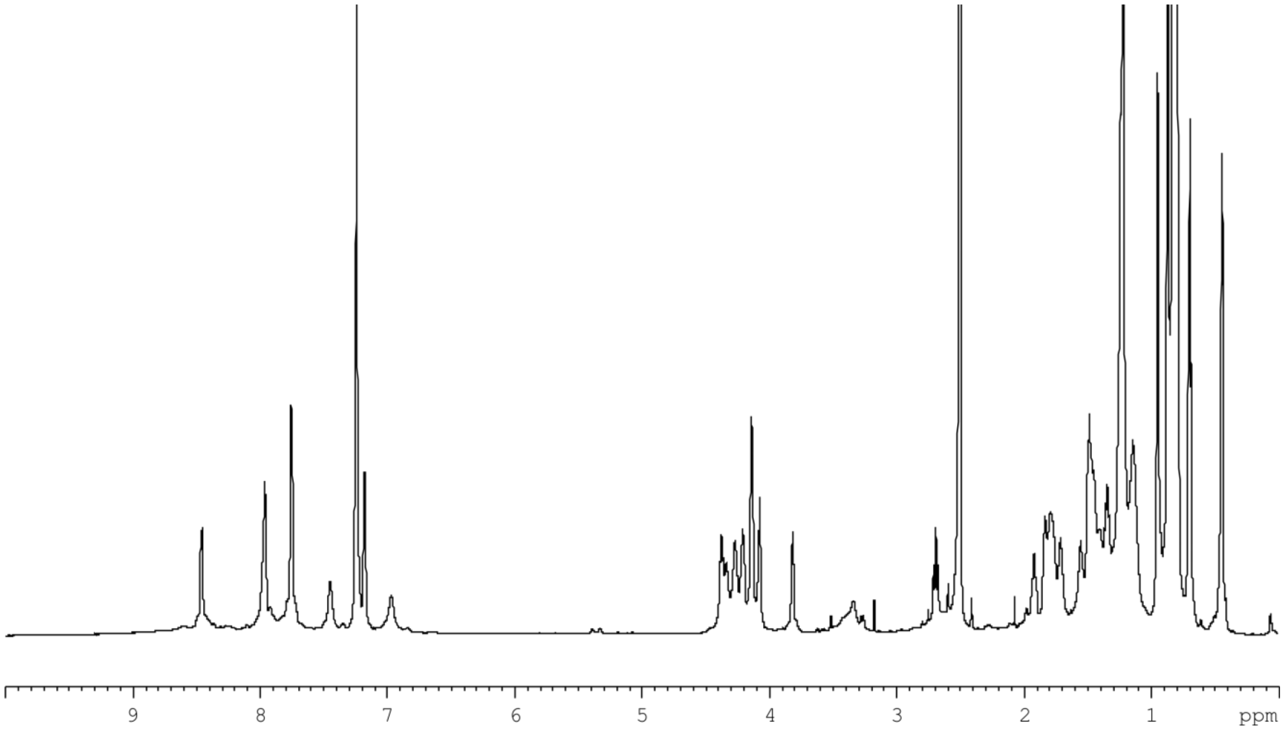

Figure S6. ^{1}H-^{13}C-HMQC of (**1a**) in d_6-DMSO at 600 MHz.

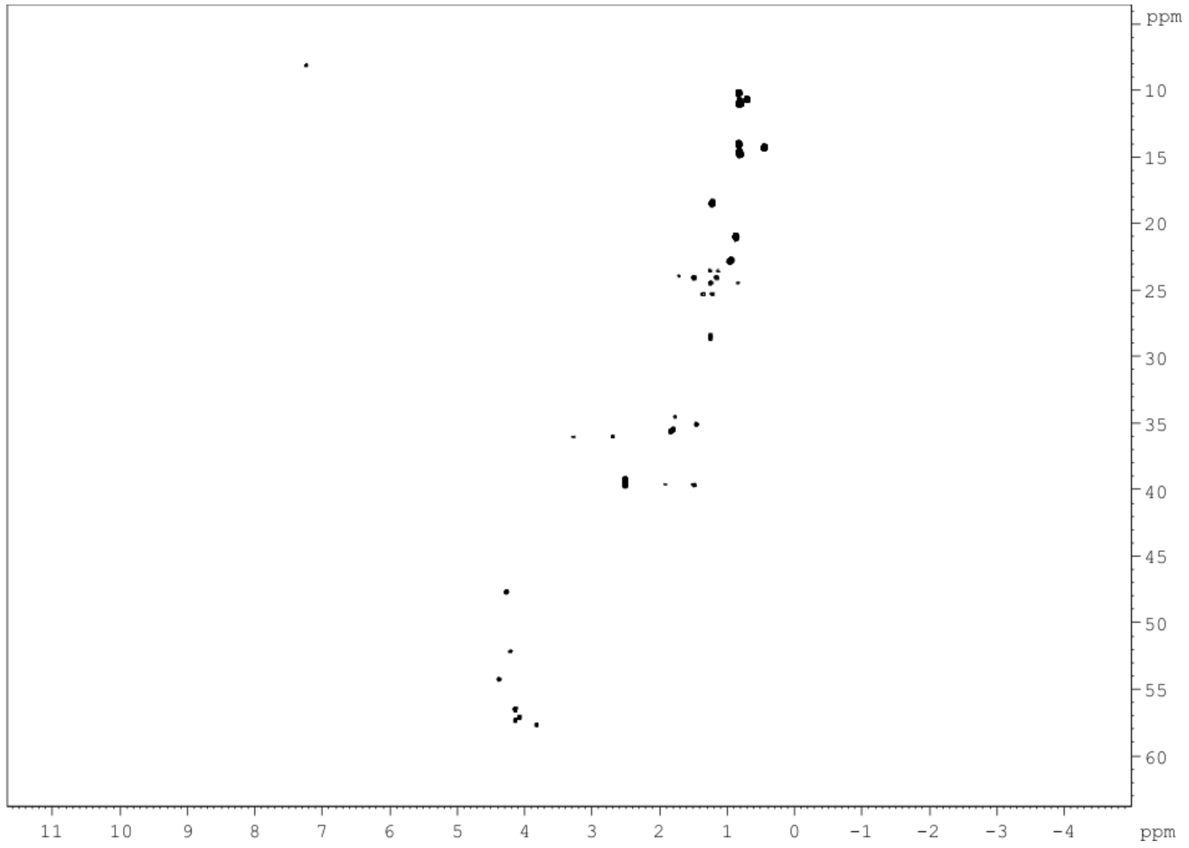

Figure S7. HMQC-TOCSY (spin lock 80 ms) of (**1a**) in d_6-DMSO at 600 MHz.

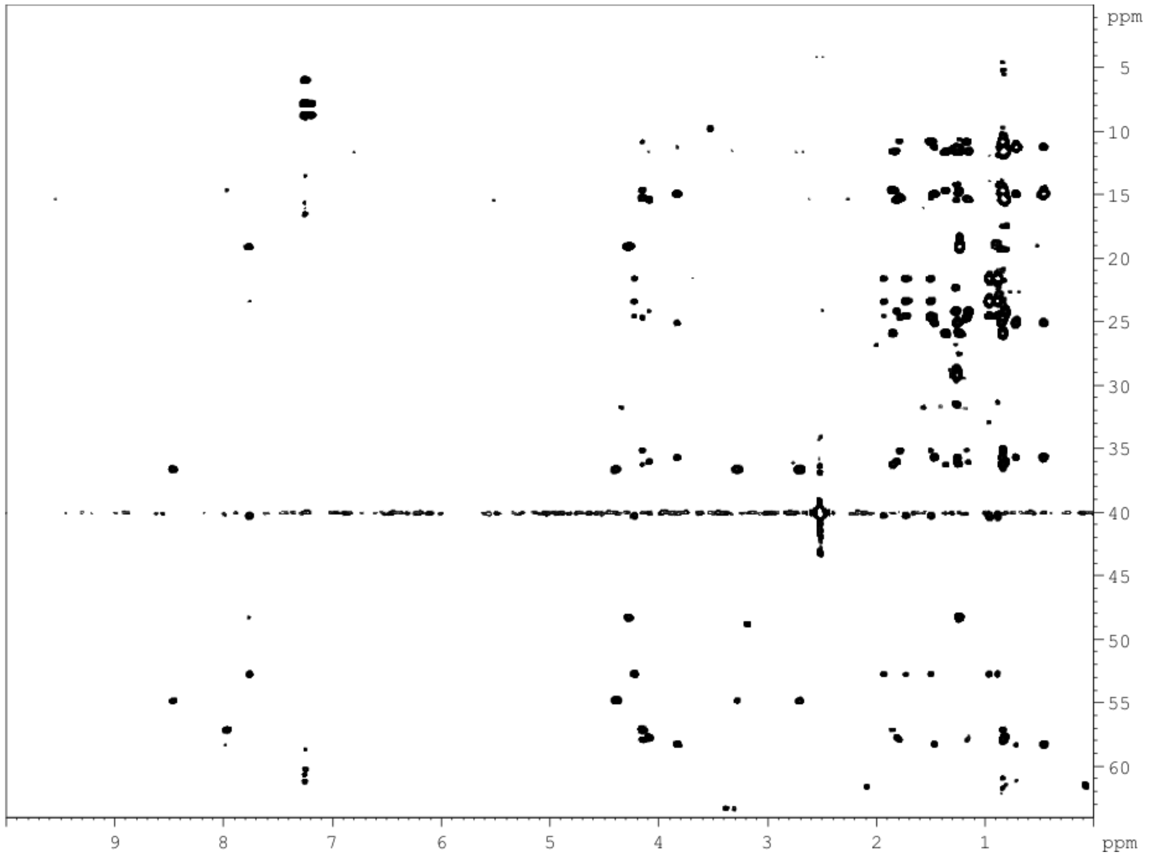

Figure S8. ^{1}H-^{13}C-HMBC of (**1a**) in d_6-DMSO at 600 MHz.

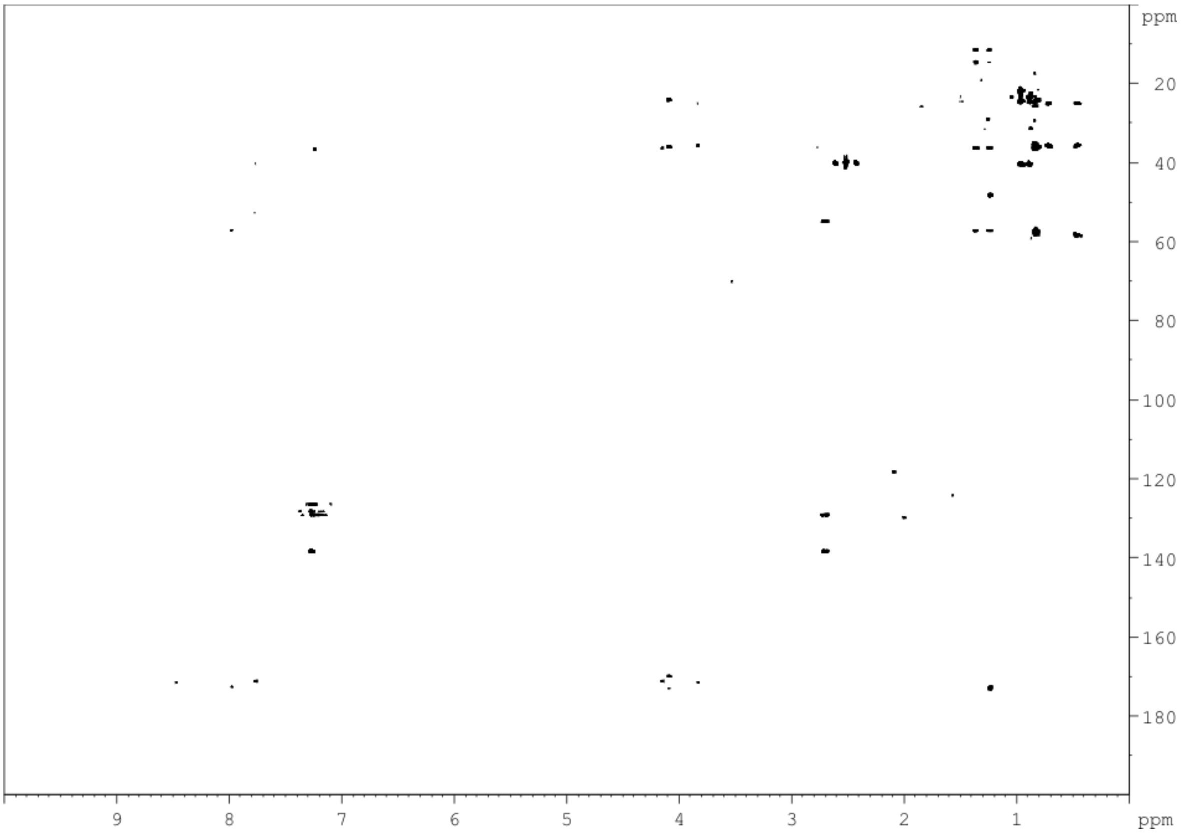

Figure S9. ^{1}H-^{1}H-COSY of (**1a**) in d_6-DMSO at 600 MHz.

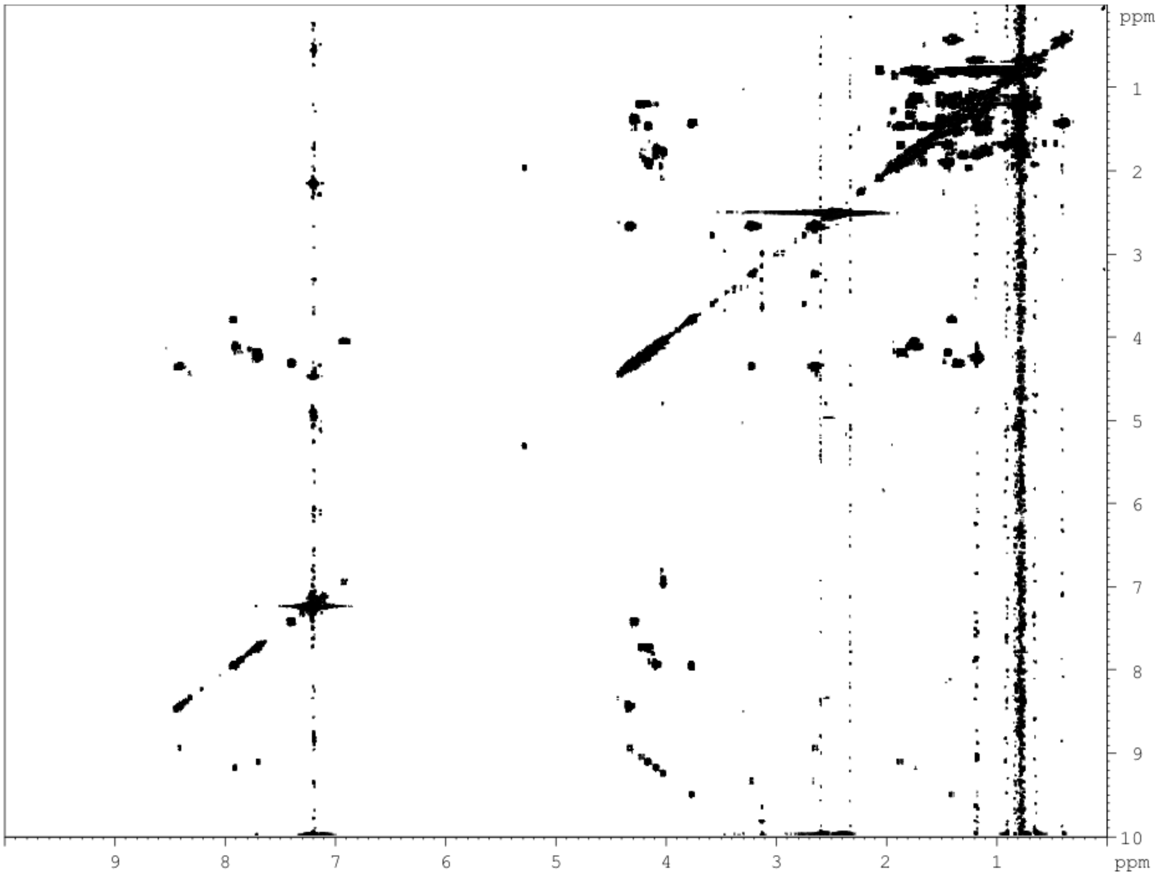

Figure S10. ^{1}H-^{1}H-NOESY (mixing time 100 ms) of (**1a**) in d_6-DMSO at 600 MHz.

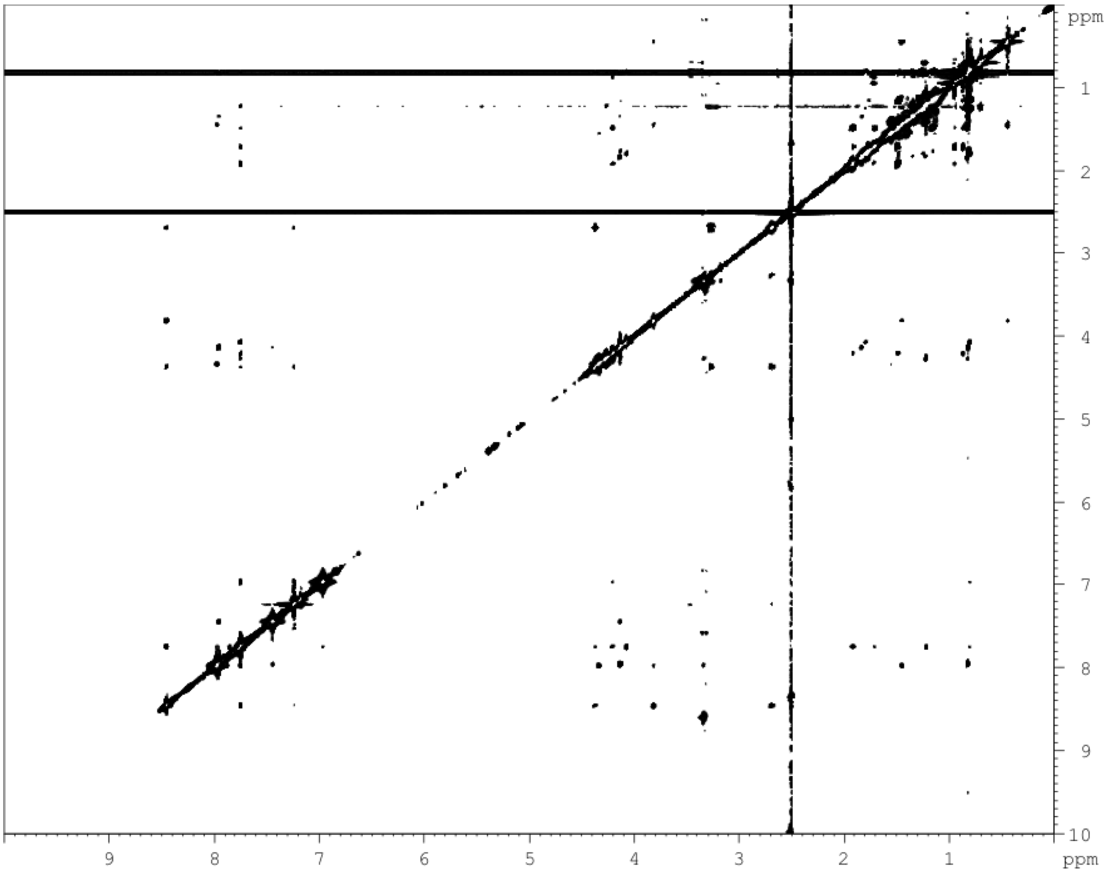

Figure S11. ^{1}H-^{1}H-TOCSY (spin lock 60 ms) data of (**1a**) in d_6-DMSO at 600 MHz.

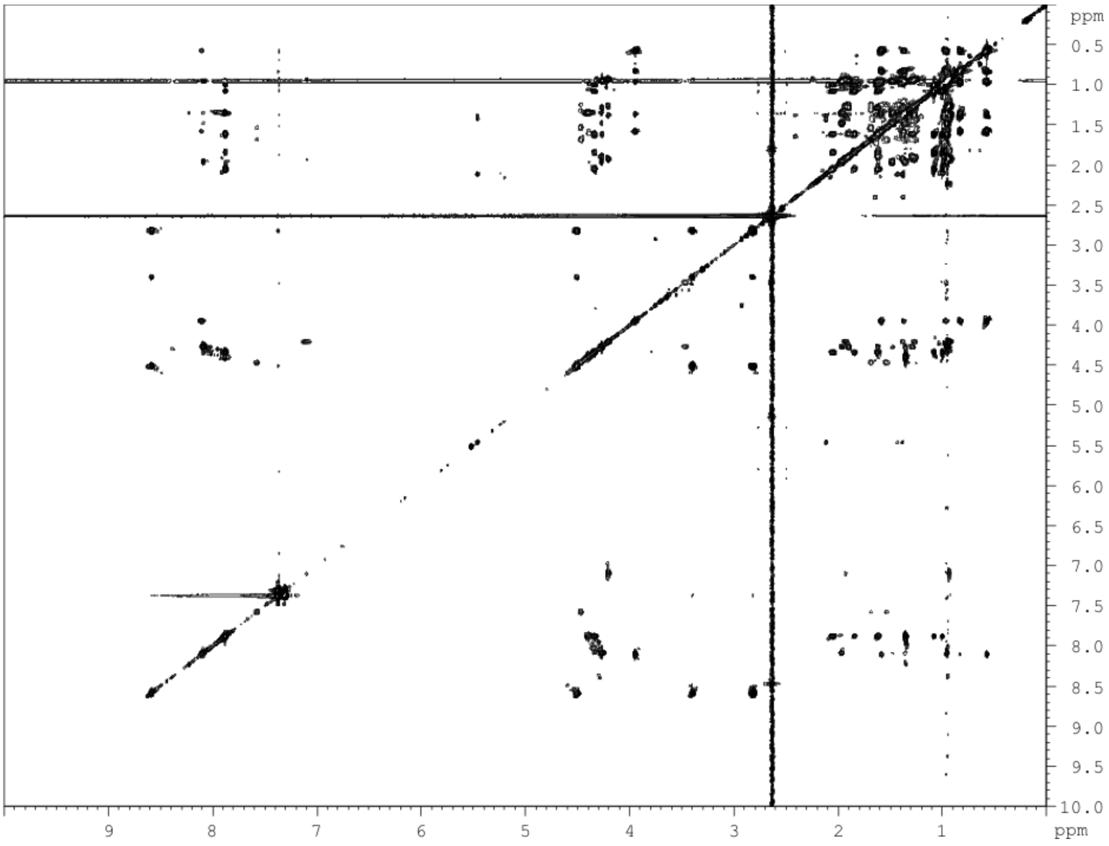

Figure S12. HPLC-ESI-(+)-MS analysis of partial hydrolysate of (**1a**) after 7 h at 110 ℃ with 6 M HCl. (**A**) EICs for dipeptides, (**B**) EICs for tripeptides (**C**) EIC for tetrapeptides. Note: Absolute stereochemistry and constitution of dipeptides was assigned by GC/MS analysis. This analysis shows the status of the hydrolysis reaction, required for subsequent derivatization and analytics. Formation of lysine-dipeptides could not be observed.

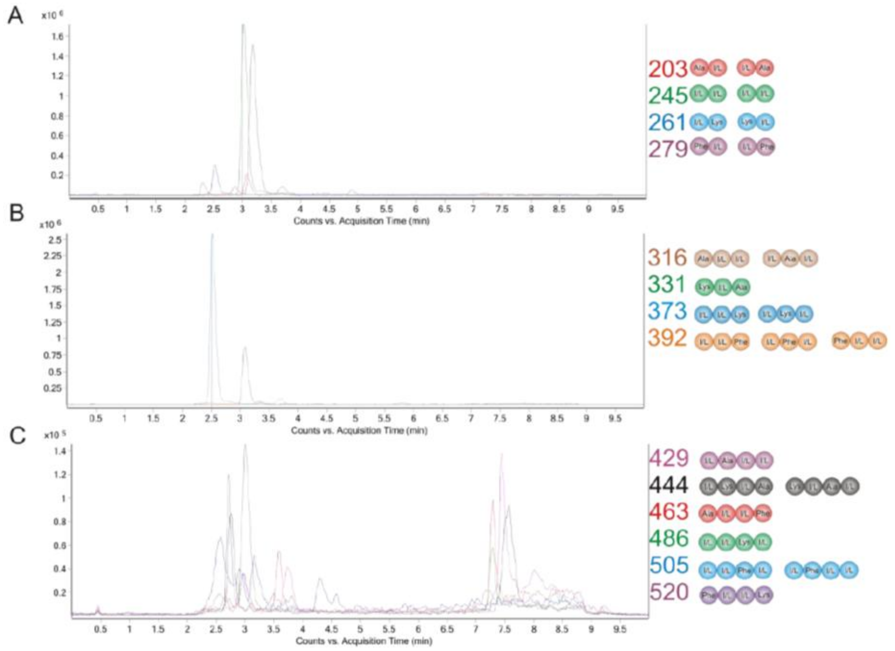

Figure S13. Chiral GC-MS analysis of total hydrolysate of (**1a**) on a Chirasil®-(L)-Val column. Amino acids were analyzed as their *N*-pentafluoropropionic 2-propyl esters and identified by their $[M + H]^+$-Ions and their fragmentation products using positive chemical ionization (PCI) with methane as reagent gas.

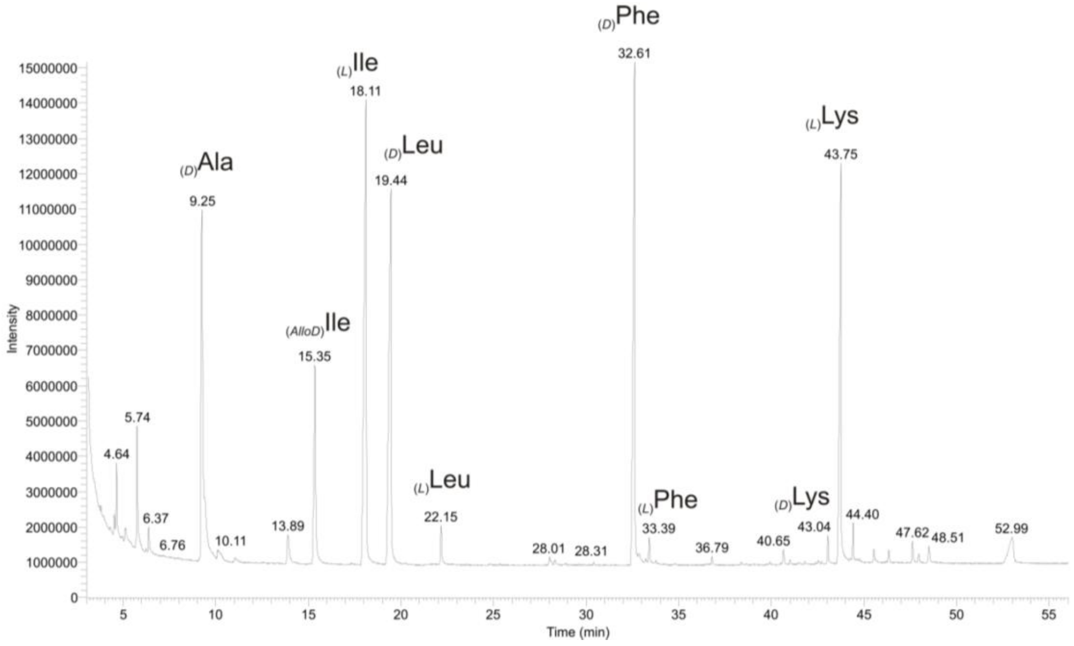

Figure S14. Chiral GC-PCI-MS analysis on a Chirasil®-(L)-Val column of Xle amino acid standards as *N*-pentafluoropropionic 2-propyl esters and identified by their $[M + H]^+$-Ions using positive chemical ionization (PCI) with methane as reagent gas.

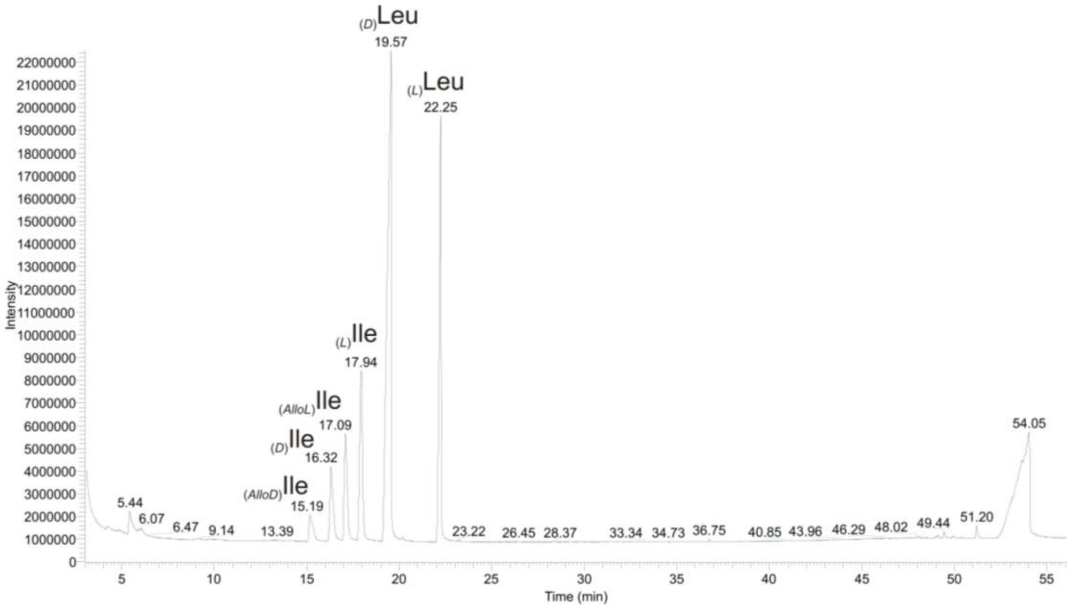

Figure S15. PCI-MS spectra of *N*-trifluoroacetyl-$_{(L)}$Ile-$_{(D)}$Ala-methyl ester found in partial hydrolysate of (**1a**) at R_t = 19.97 min on a Chirasil®-(L)-Val column.

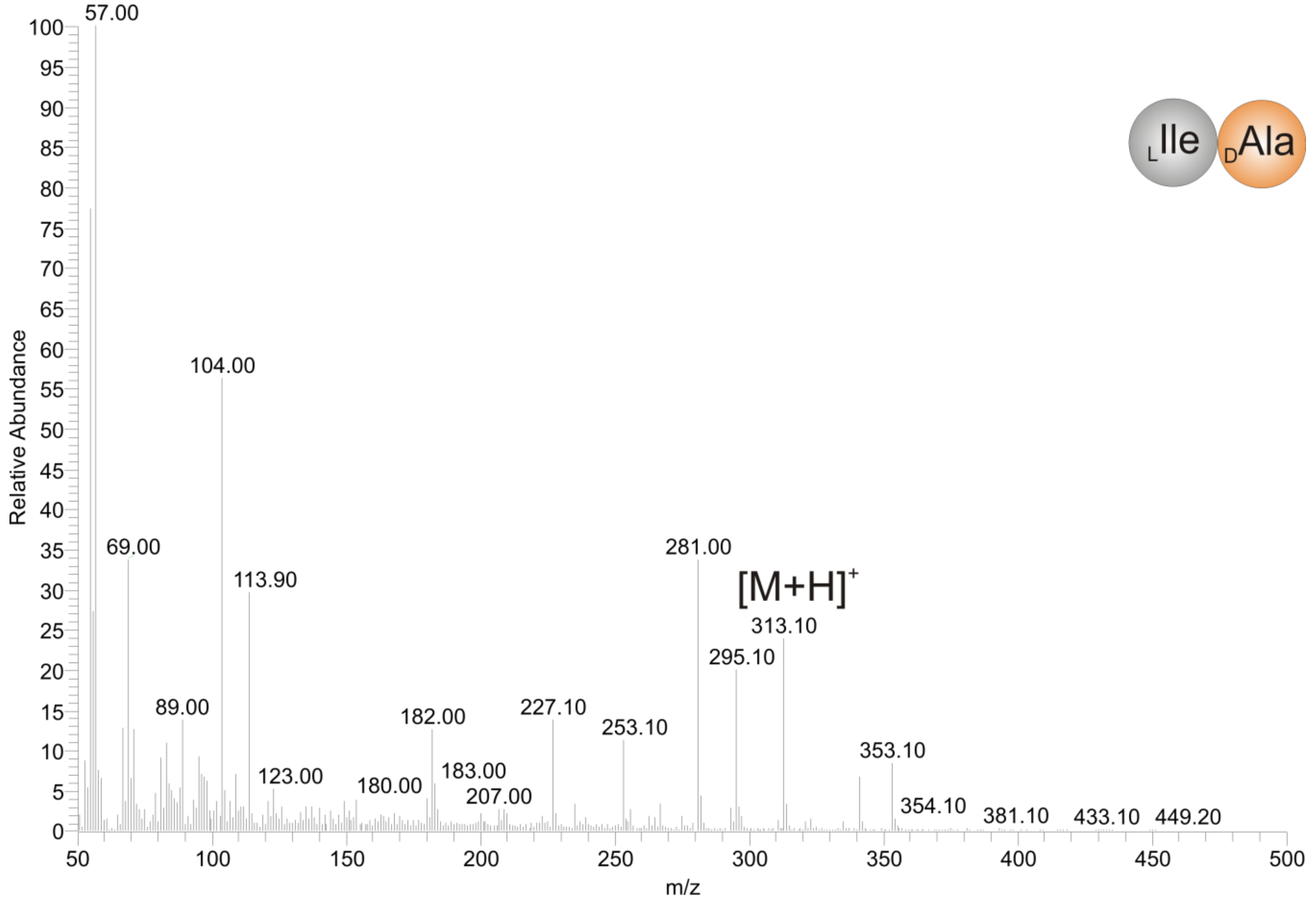

Figure S16. EI-MS spectra of *N*-trifluoroacetyl-$_{(L)}$Ile-$_{(D)}$Ala-methyl ester found in partial hydrolysate of (**1a**) at R_t = 19.97 min on a Chirasil®-(L)-Val column.

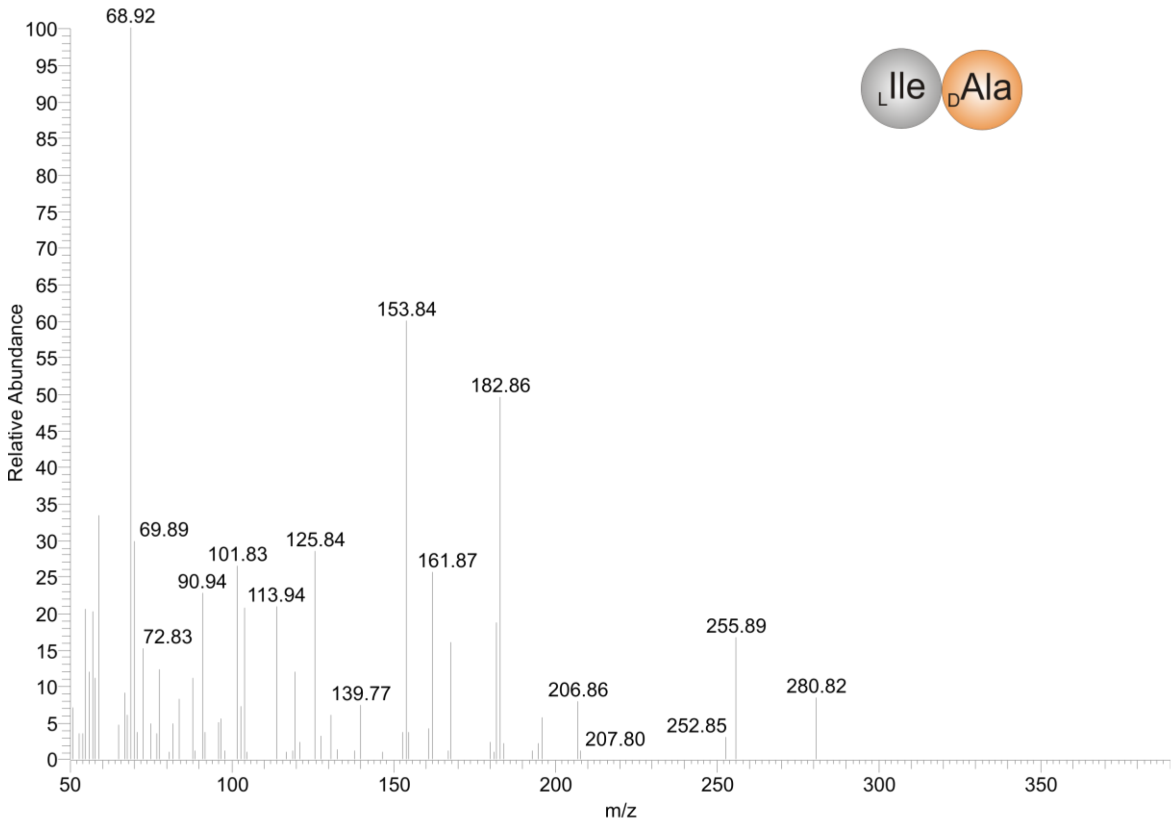

Figure S17. PCI-MS spectra of *N*-trifluoroacetyl-$_{(L)}$Ile-$_{(L)}$Ile-methyl ester found in partial hydrolysate of (**1a**) at R_t = 22.5 min on a Chirasil®-(L)-Val column.

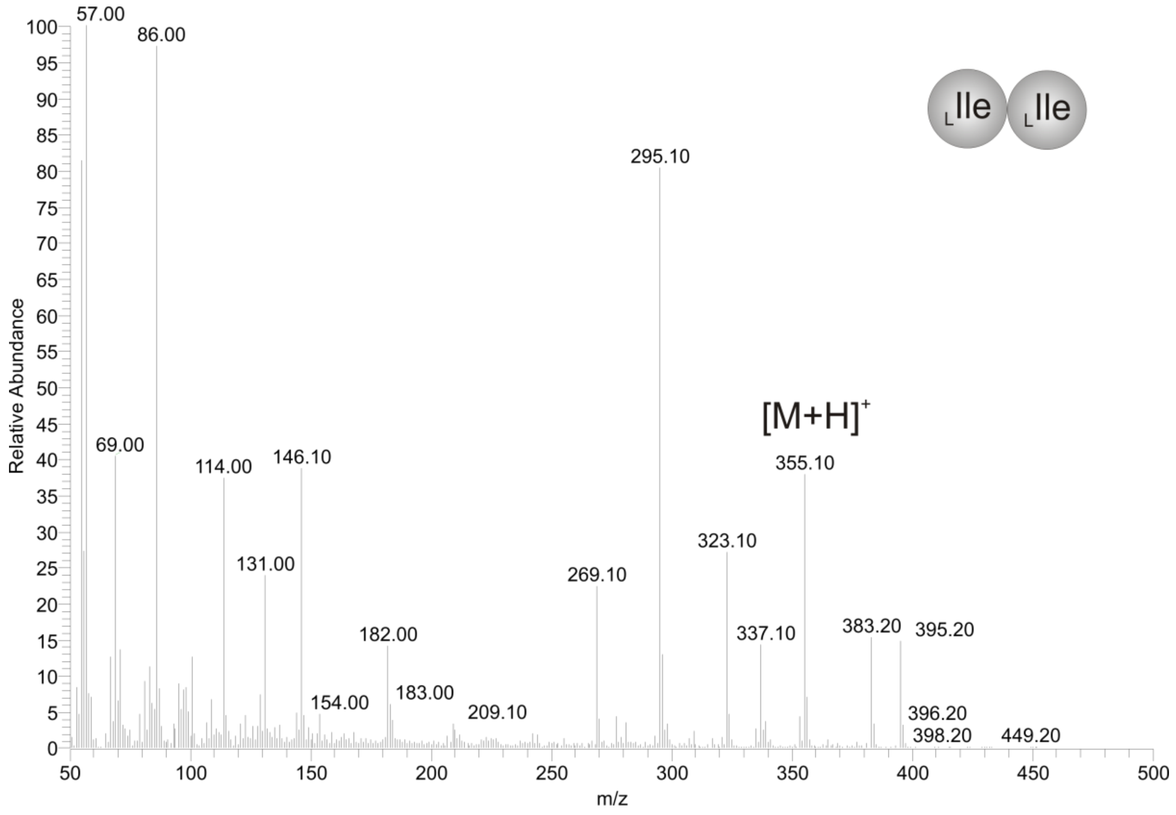

Figure S18. EI-MS spectra of *N*-trifluoroacetyl-$_{(L)}$Ile-$_{(L)}$Ile-methyl ester found in partial hydrolysate of (**1a**) at R_t = 22.5 min on a Chirasil®-(L)-Val column.

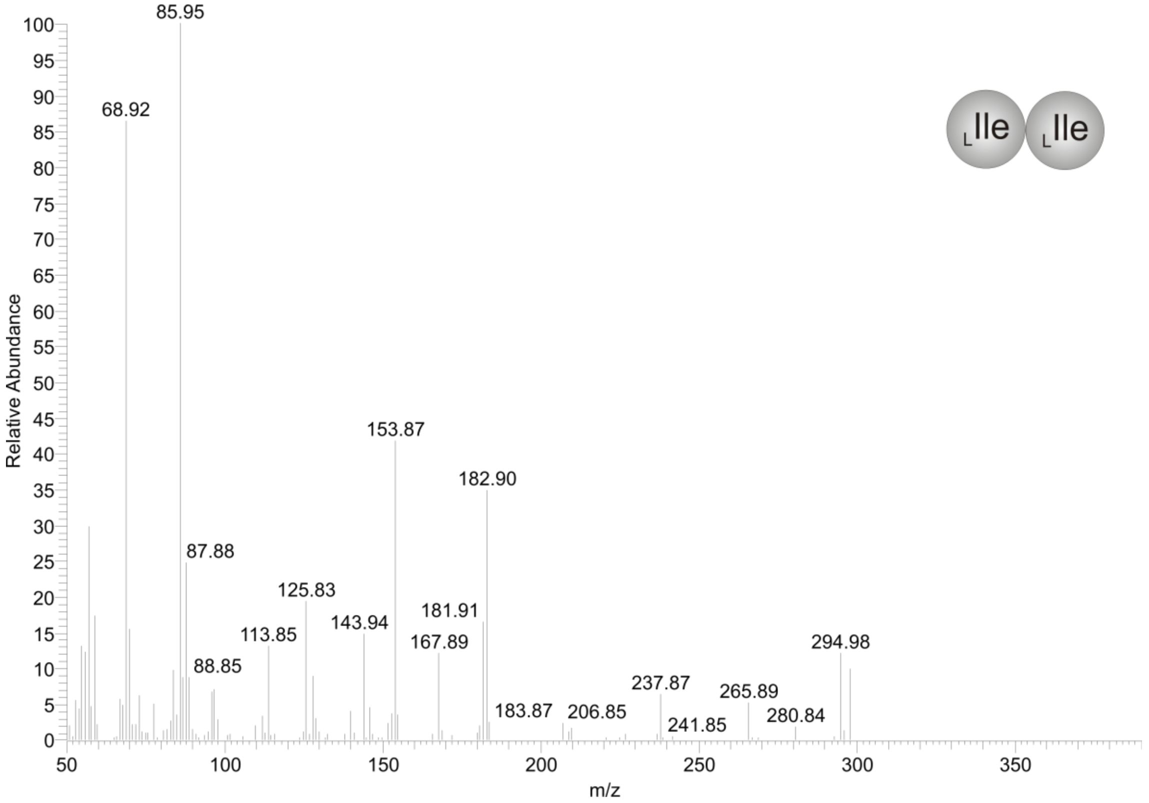

Figure S19. PCI-MS spectra of *N*-trifluoroacetyl-$_{(D)}$Leu-$_{(L)}$Ile-methyl ester found in partial hydrolysate of (**1a**) at R_t = 24.6 min on a Chirasil®-(L)-Val column.

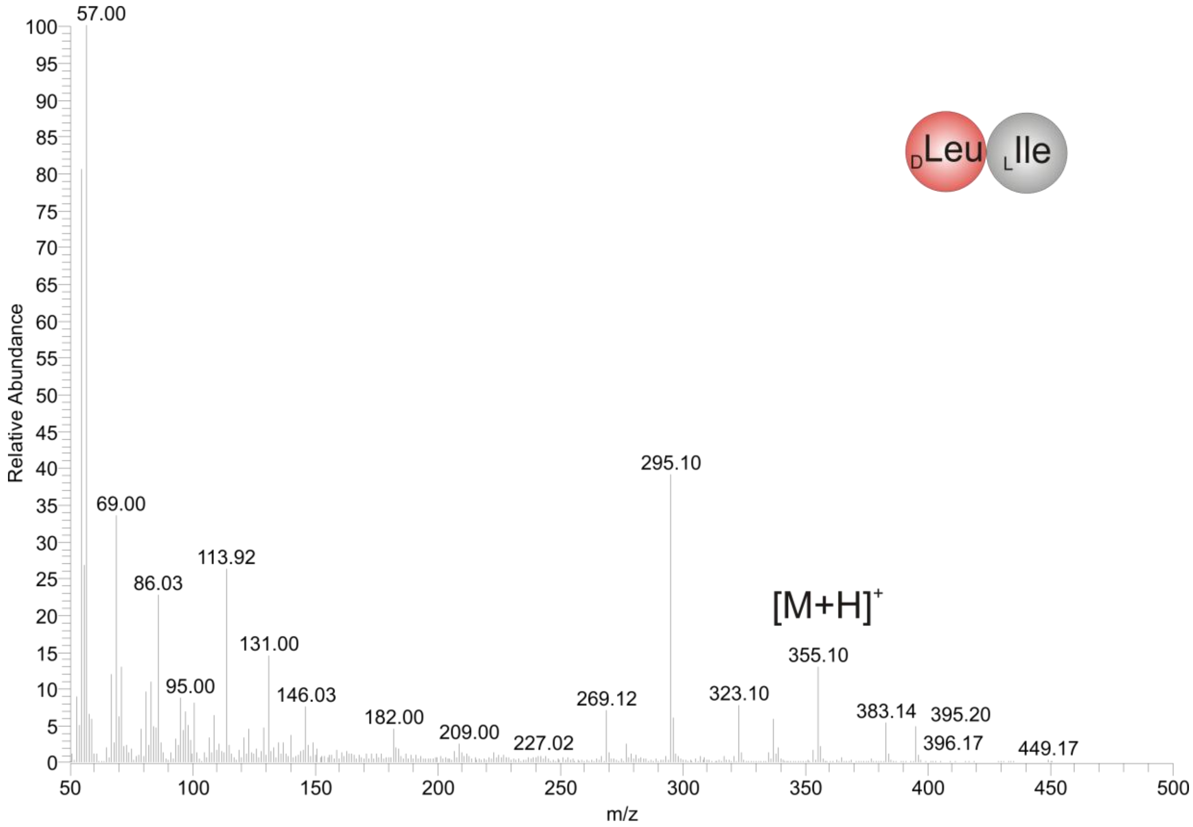

Figure S20. EI-MS spectra of *N*-trifluoroacetyl-$_{(D)}$Leu-$_{(L)}$Ile-methyl ester found in partial hydrolysate of (**1a**) at R_t = 24.6 min on a Chirasil®-(L)-Val column.

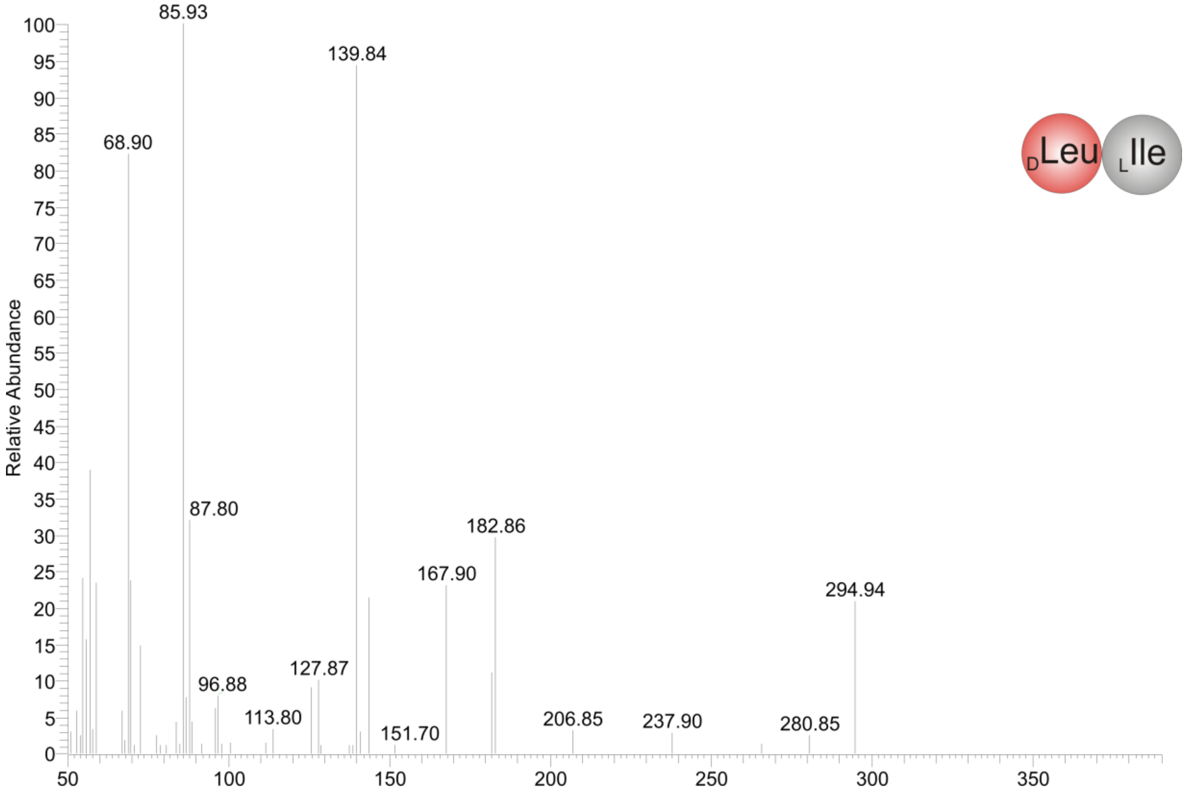

Figure S21. PCI-MS spectra of *N*-trifluoroacetyl-$_{(L)}$Ile-$_{(D)}$Phe-methyl ester found in partial hydrolysate of (**1a**) at R_t = 46.68 min on a Chirasil®-(L)-Val column.

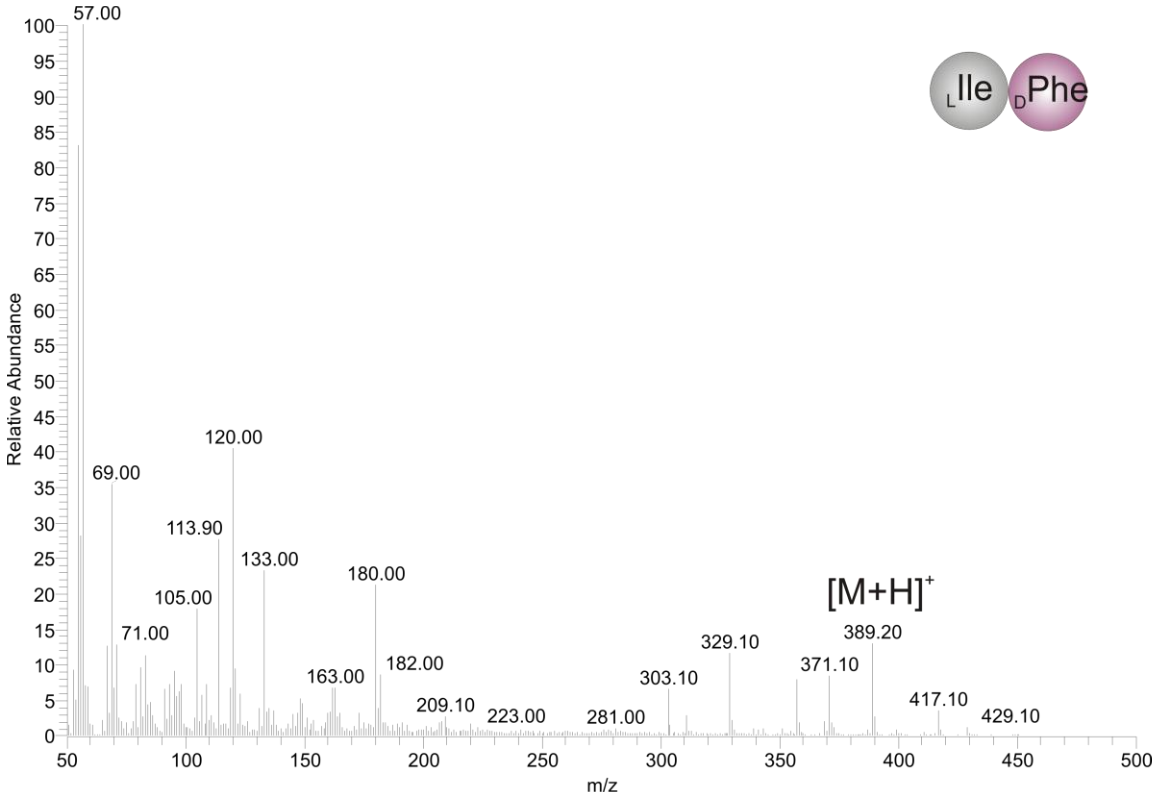

Figure S22. EI-MS spectra of *N*-trifluoroacetyl-$_{(L)}$Ile-$_{(D)}$Phe-methyl ester found in partial hydrolysate of (**1a**) at R_t = 46.68 min on a Chirasil®-(L)-Val column.

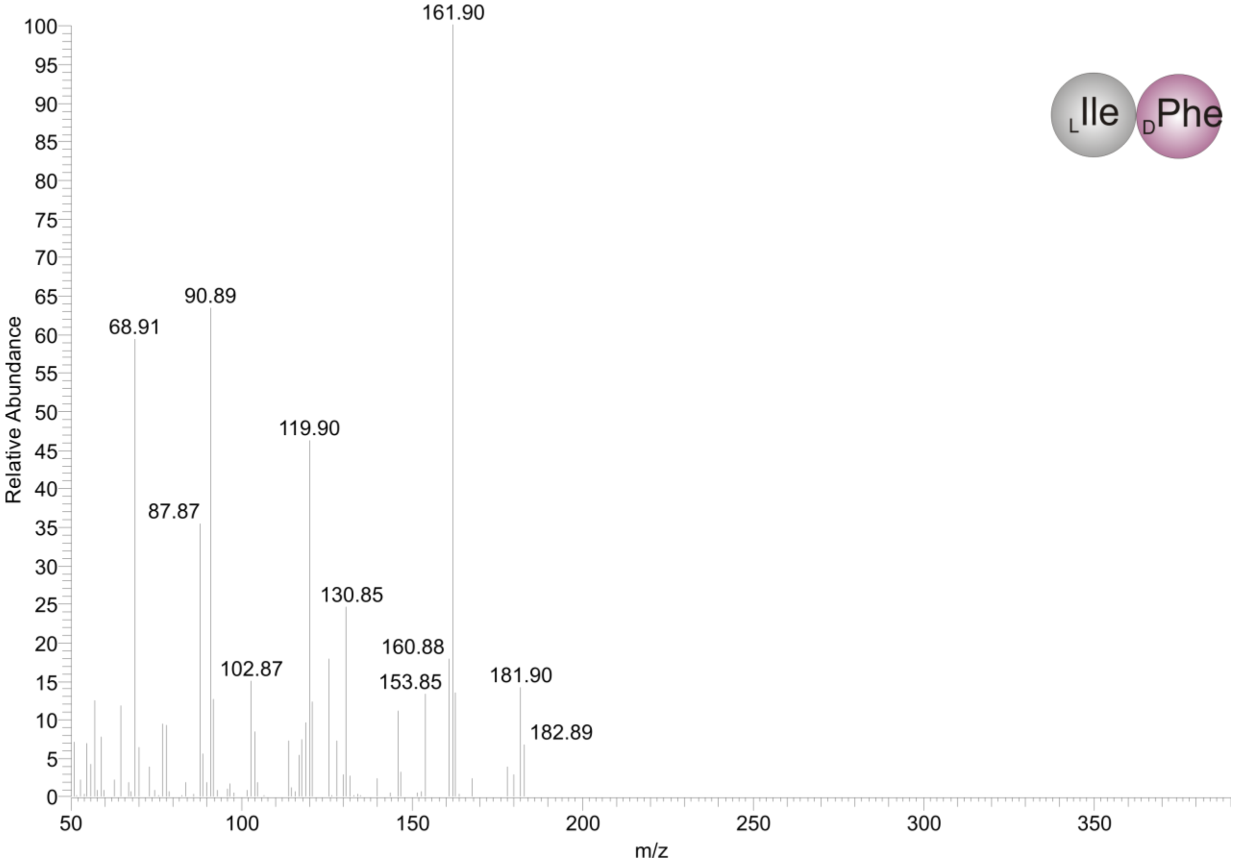

Figure S23. PCI-MS spectra of synthetic *N*-trifluoroacetyl-Leu-Leu-methyl ester.

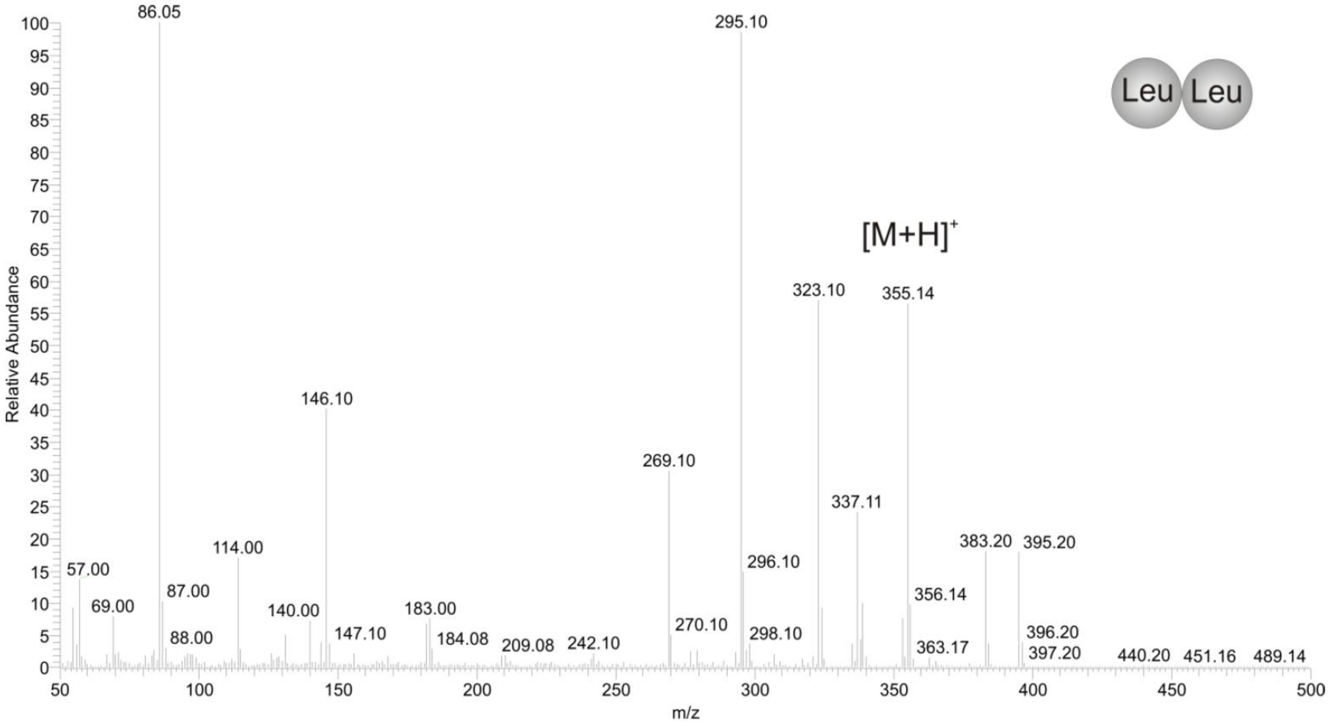

Figure S24. EI-MS spectra of synthetic *N*-trifluoroacetyl-Leu-Leu-methyl ester.

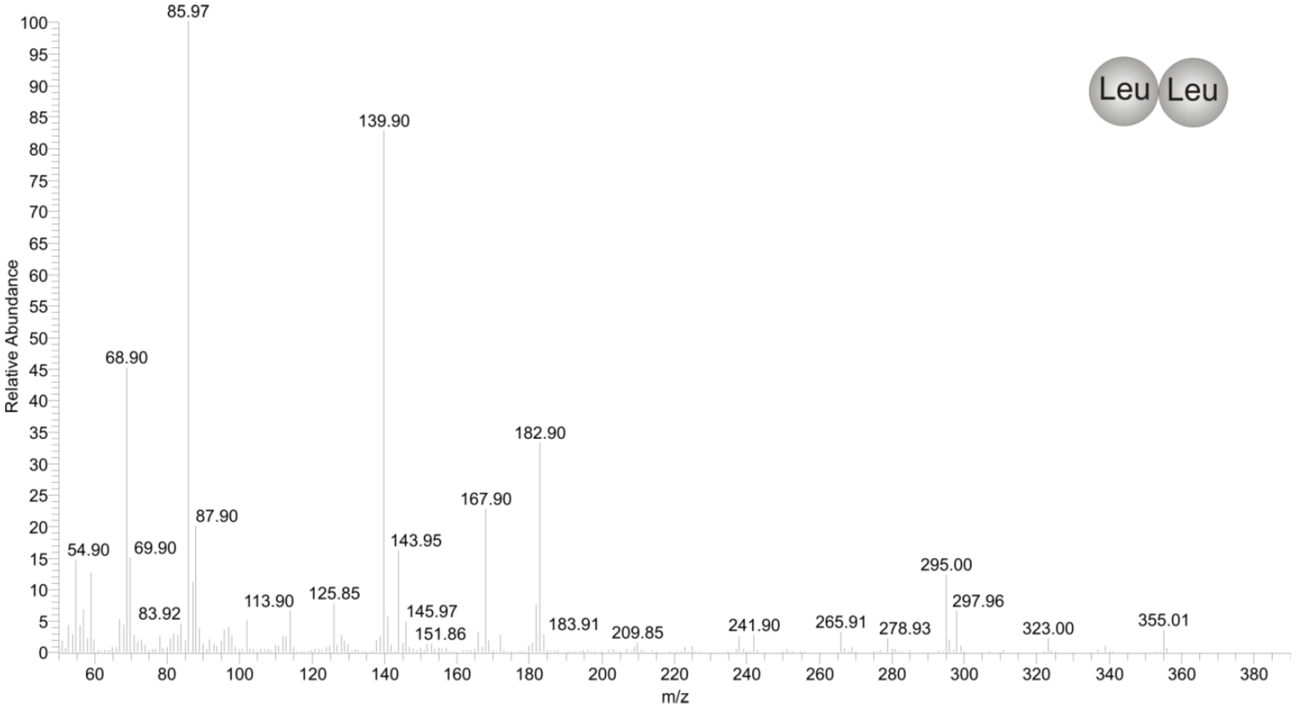

Figure S25. PCI-MS spectra of synthetic *N*-trifluoroacetyl-Ile-Ile-methyl ester.

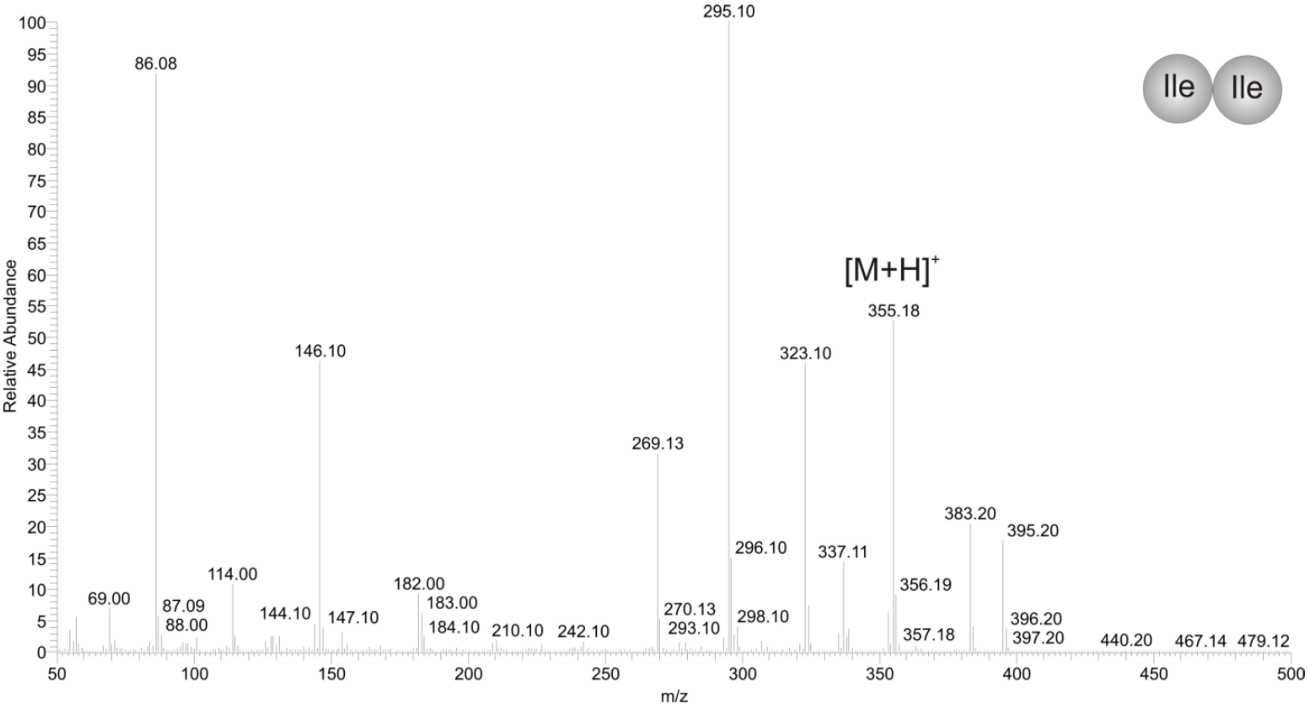

Figure S26. EI-MS spectra of synthetic *N*-trifluoroacetyl-Ile-Ile-methyl ester.

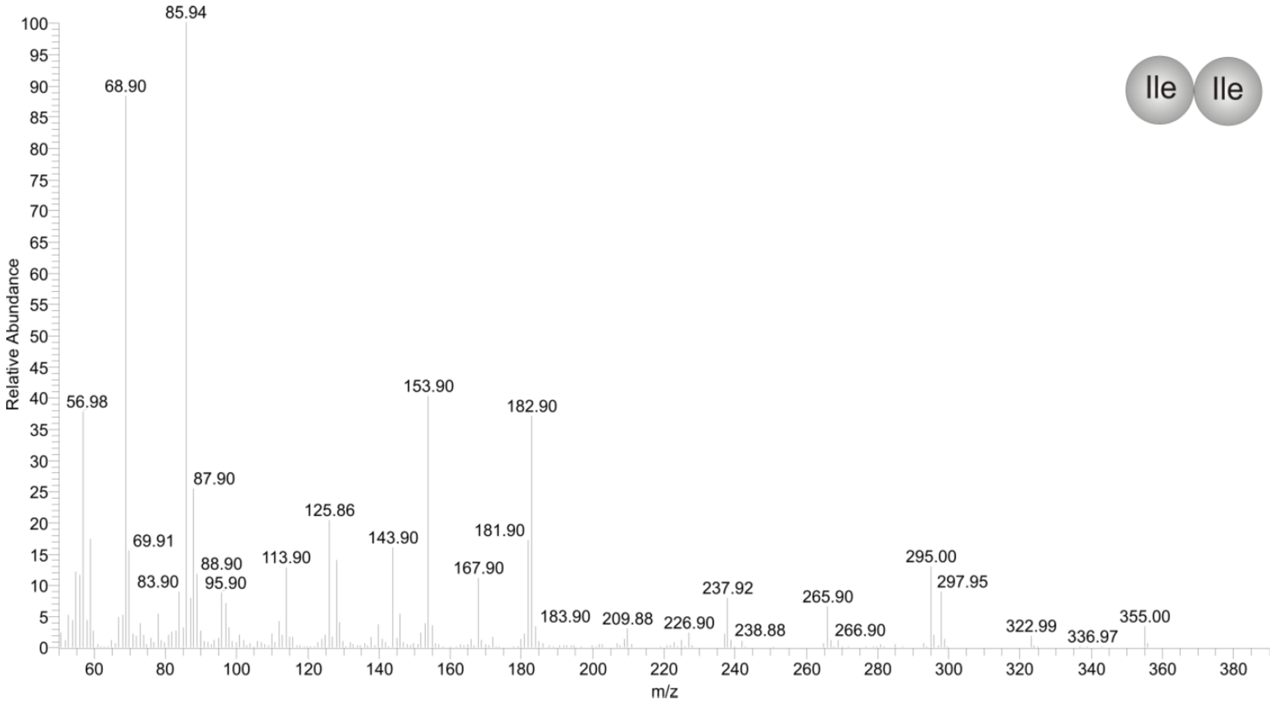

Figure S27. PCI-MS spectra of synthetic *N*-trifluoroacetyl-Leu-Ile-methyl ester.

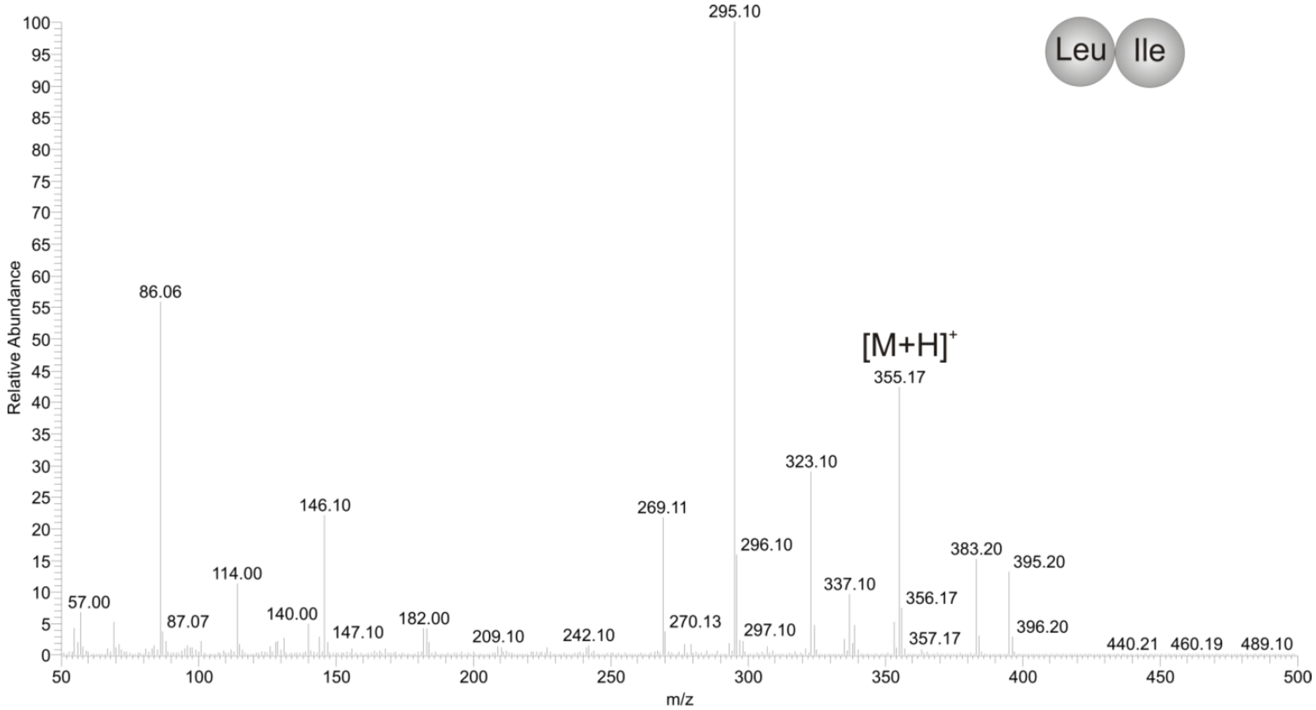

Figure S28. EI-MS spectra of synthetic *N*-trifluoroacetyl-Leu-Ile-methyl ester.

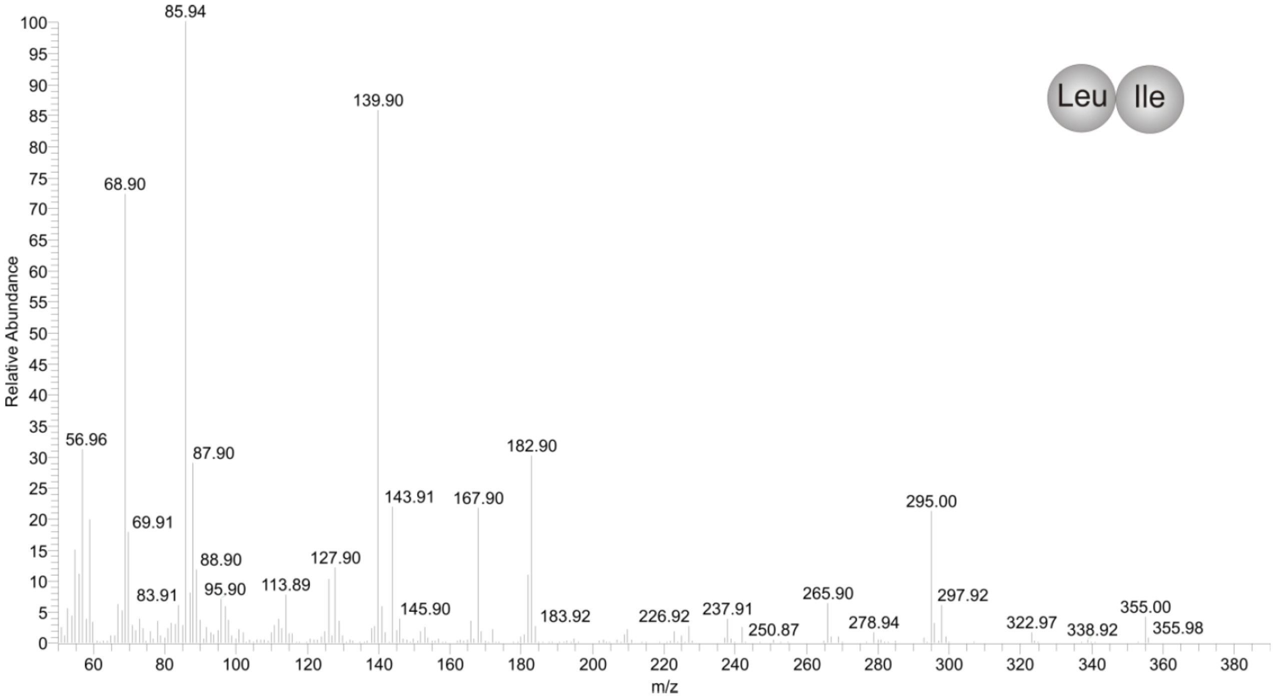

Figure S29. PCI-MS spectra of synthetic *N*-trifluoroacetyl-Ile-Leu-methyl ester.

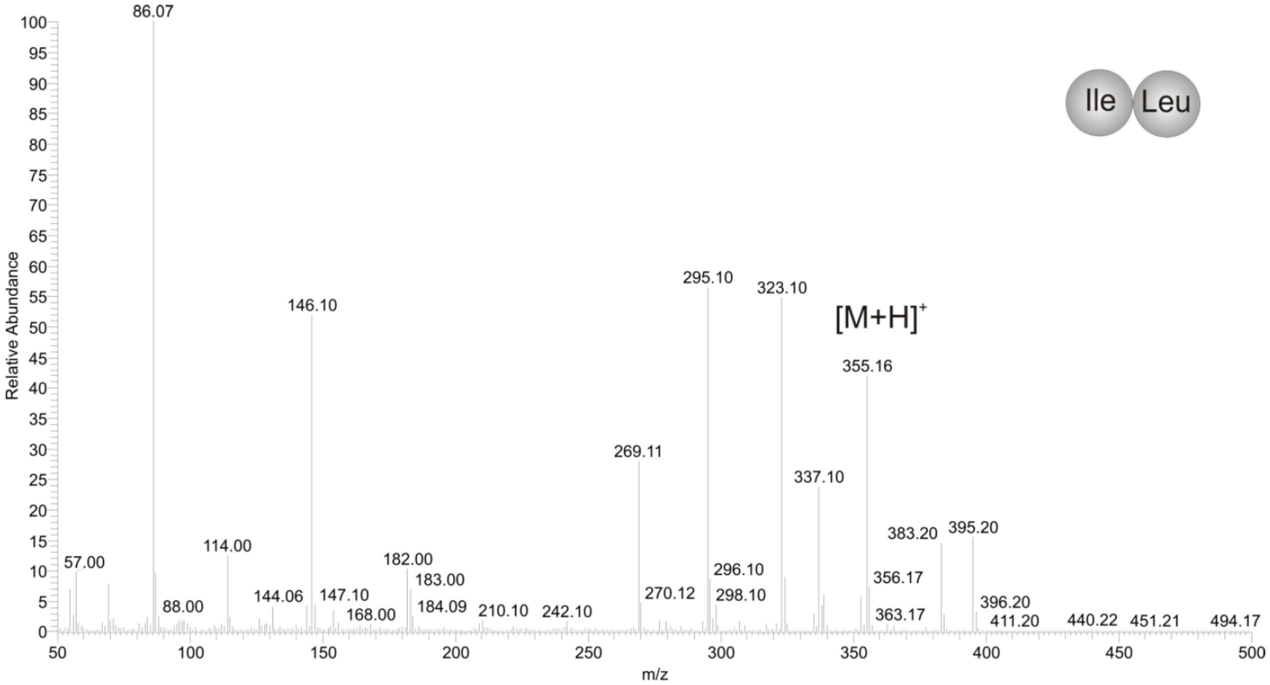

Figure S30. EI-MS spectra of synthetic *N*-trifluoroacetyl-Ile-Leu-methyl ester.

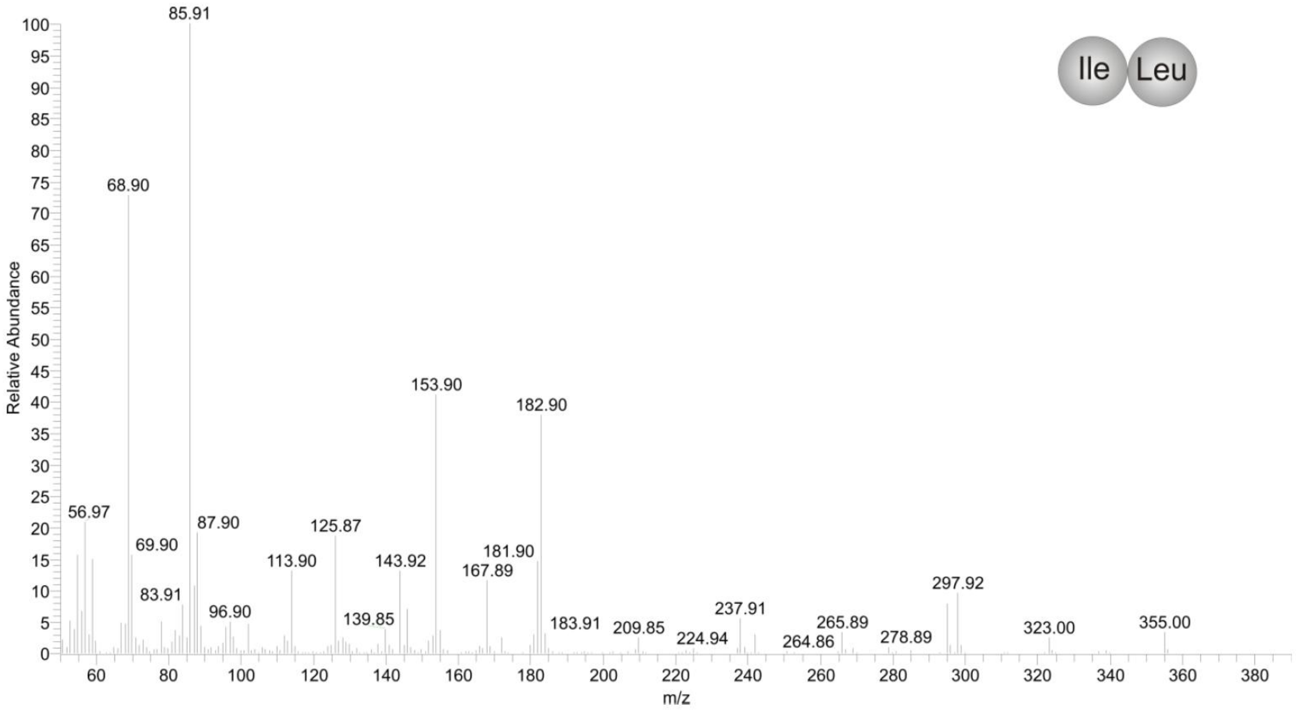

Figure S31. PCI-MS spectra of synthetic *N*-trifluoroacetyl-Ala-Leu-methyl ester.

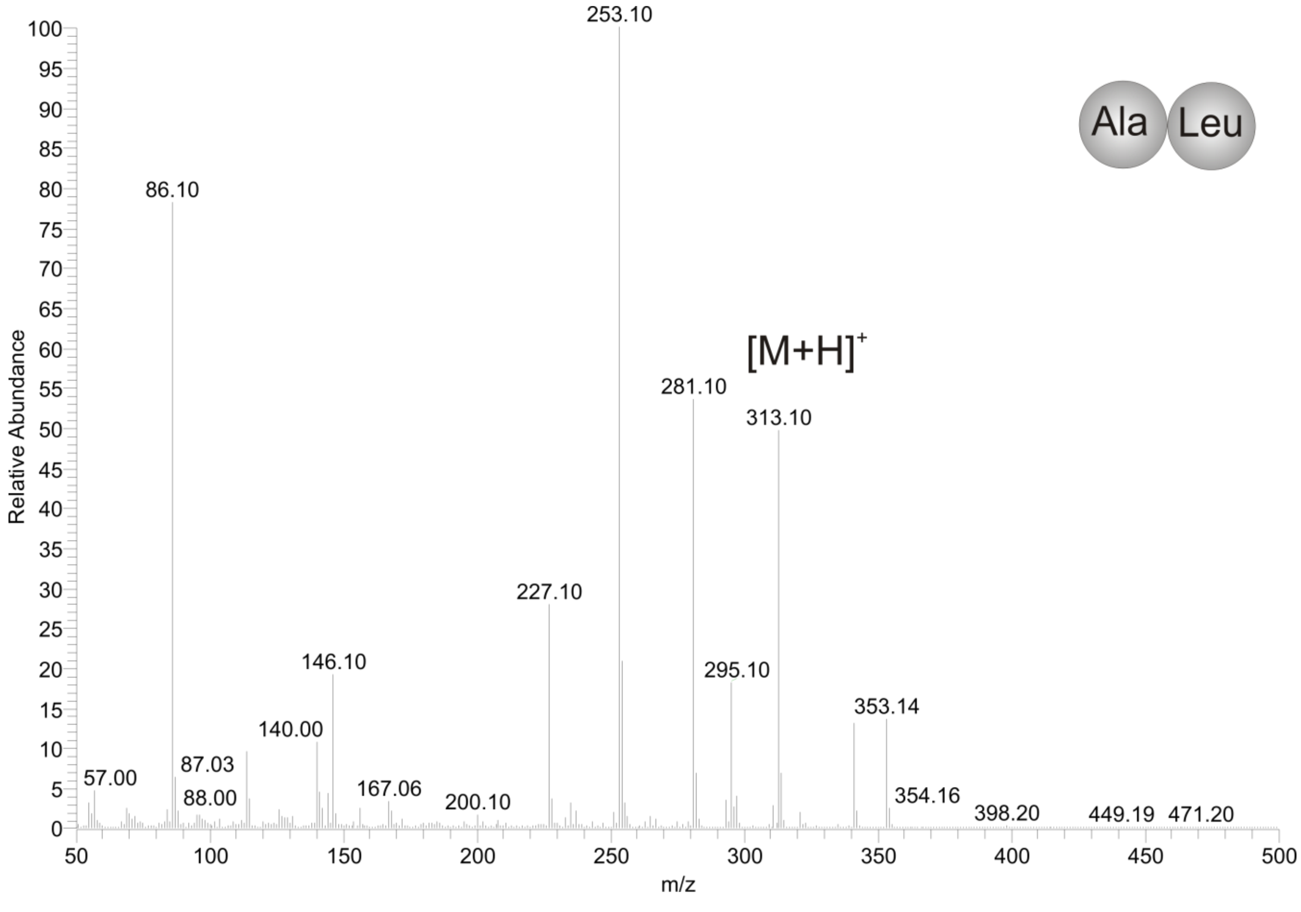

Figure S32. EI-MS spectra of synthetic *N*-trifluoroacetyl-Ala-Leu-methyl ester.

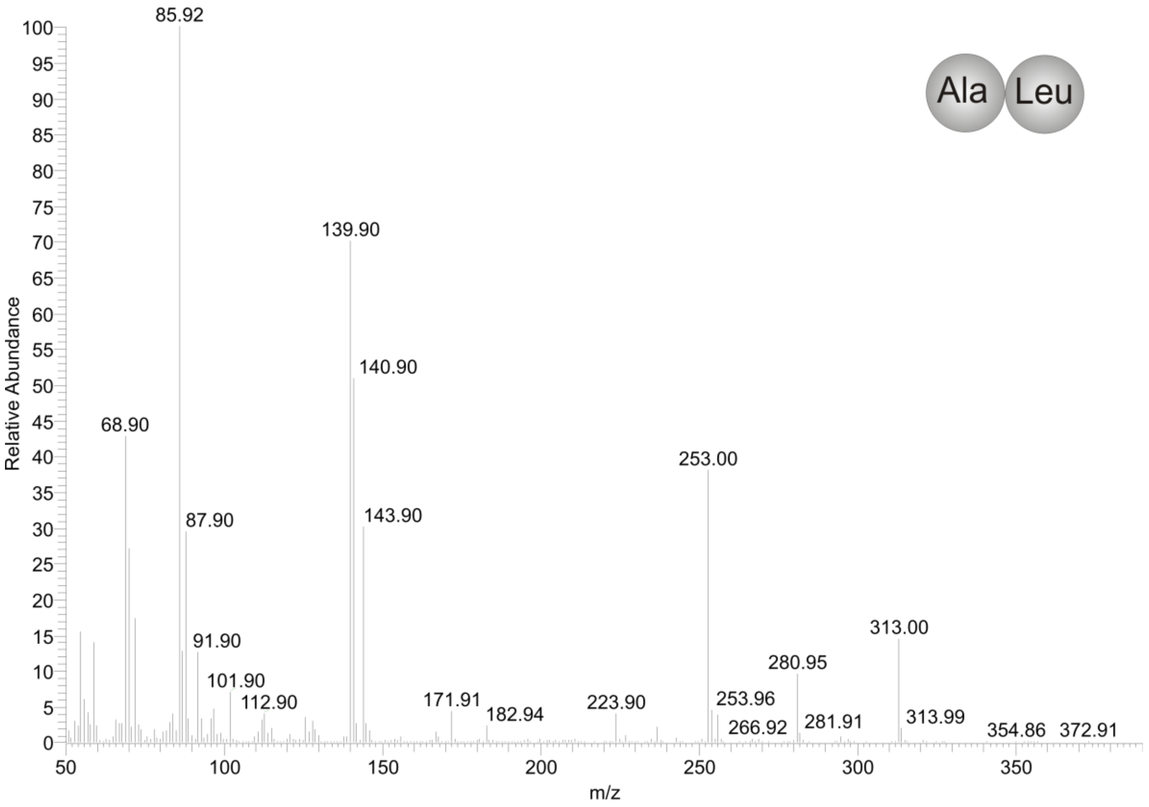

Figure S33. PCI-MS spectra of synthetic *N*-trifluoroacetyl-Leu-Ala-methyl ester.

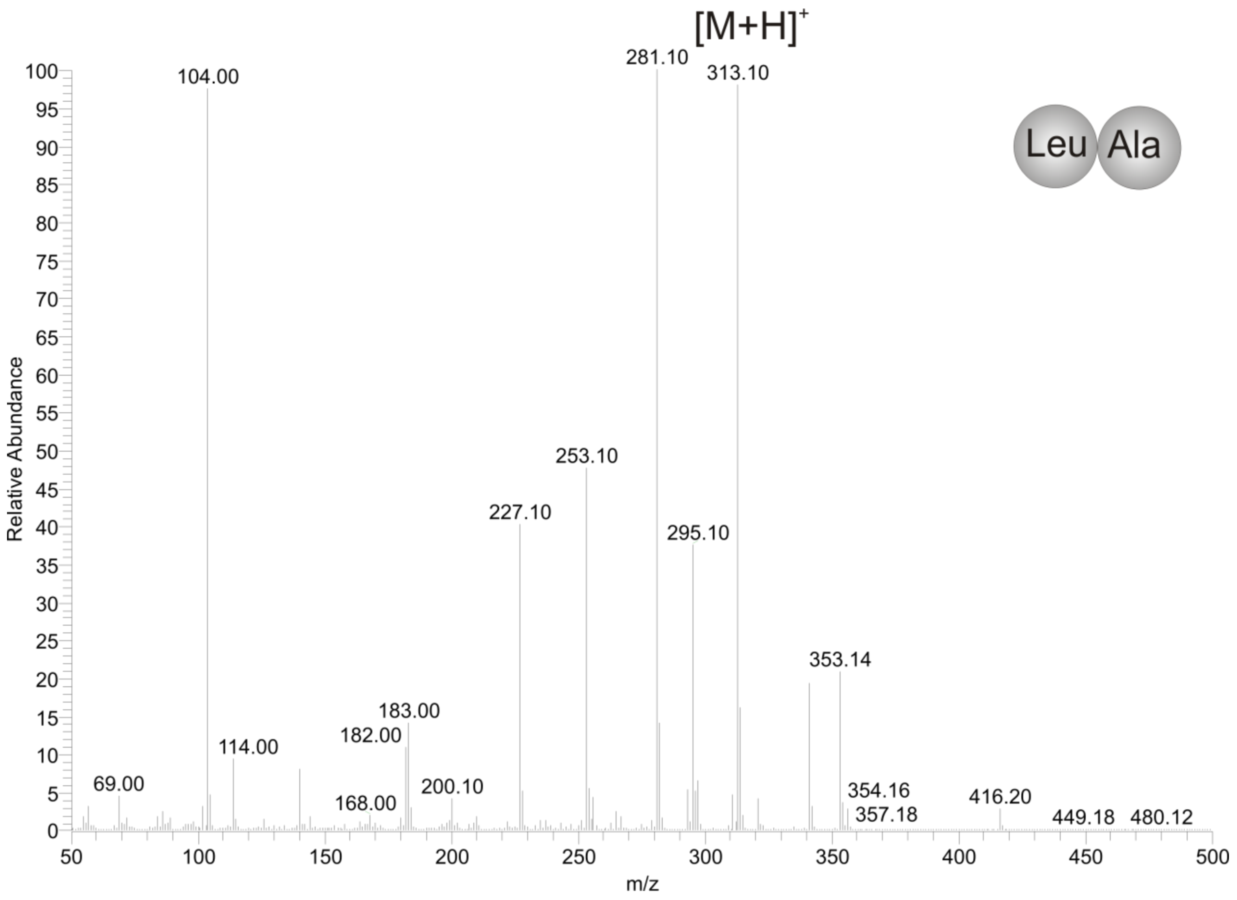

Figure S34. EI-MS spectra of synthetic *N*-trifluoroacetyl-Leu-Ala-methyl ester.

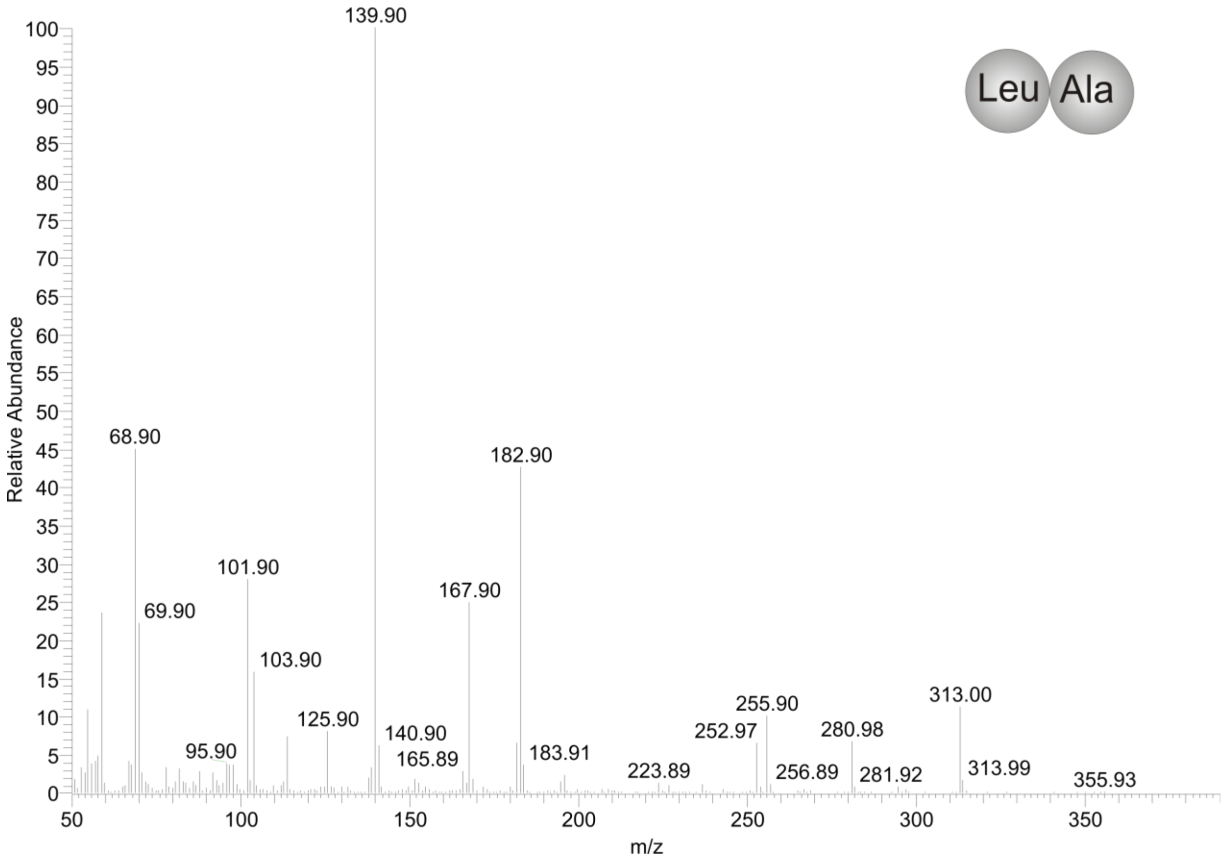

Figure S35. PCI-MS spectra of synthetic *N*-trifluoroacetyl-Ala-Ile-methyl ester.

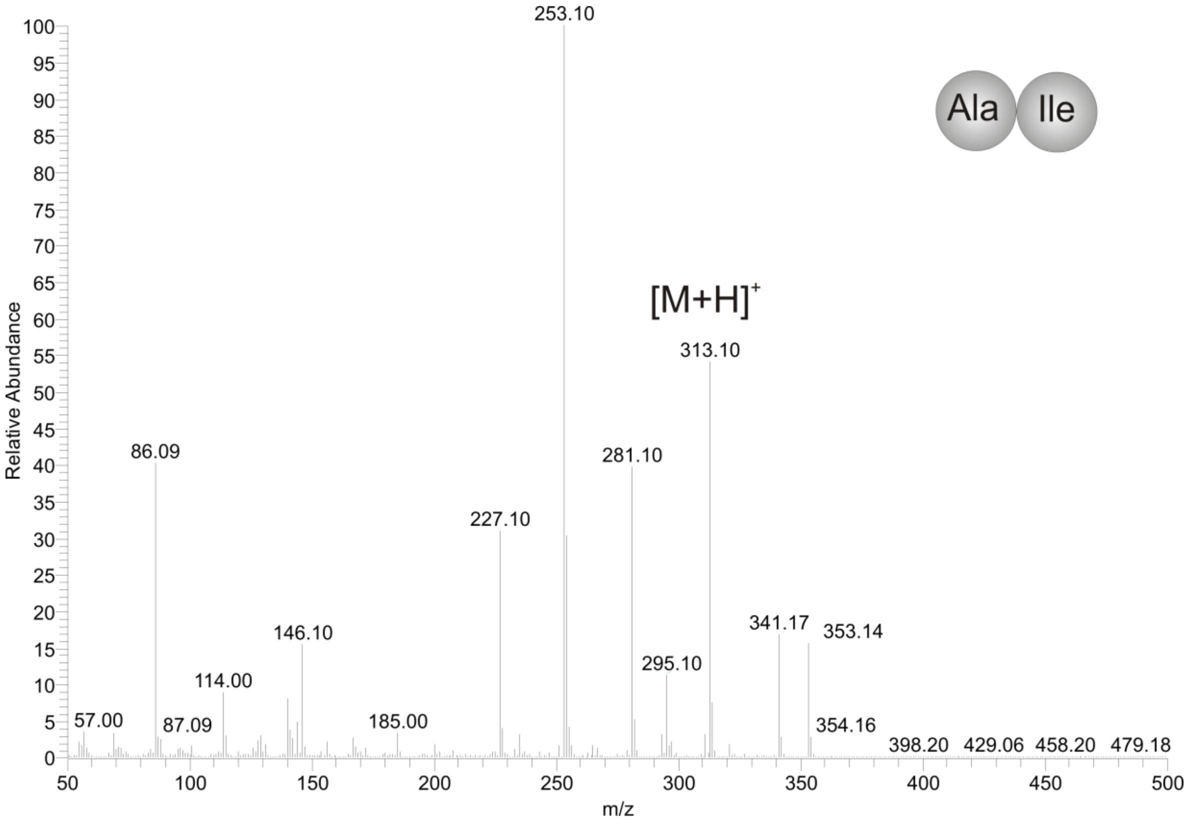

Figure S36. EI-MS spectra of synthetic *N*-trifluoroacetyl-Ala-Ile-methyl ester.

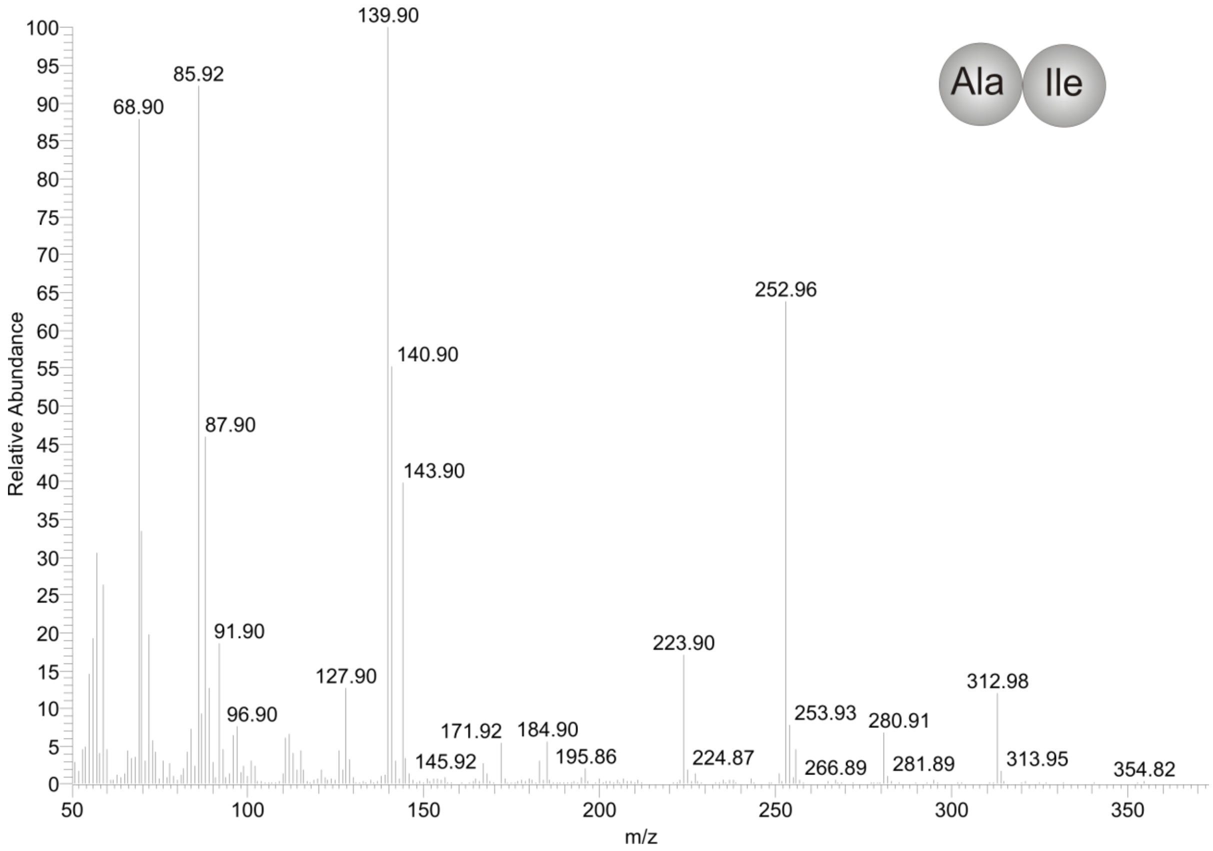

Figure S37. PCI-MS spectra of synthetic *N*-trifluoroacetyl-Ile-Ala-methyl ester.

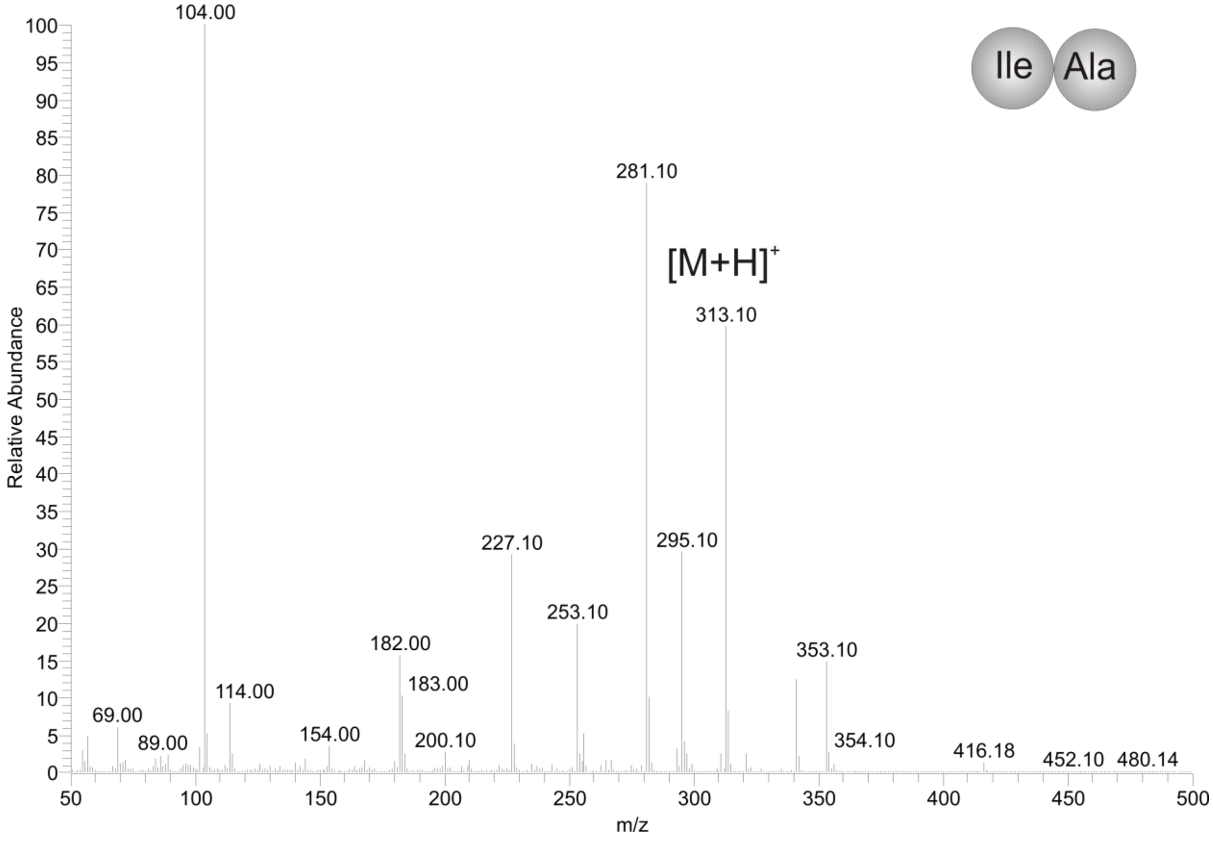

Figure S38. EI-MS spectra of synthetic *N*-trifluoroacetyl-Ile-Ala-methyl ester.

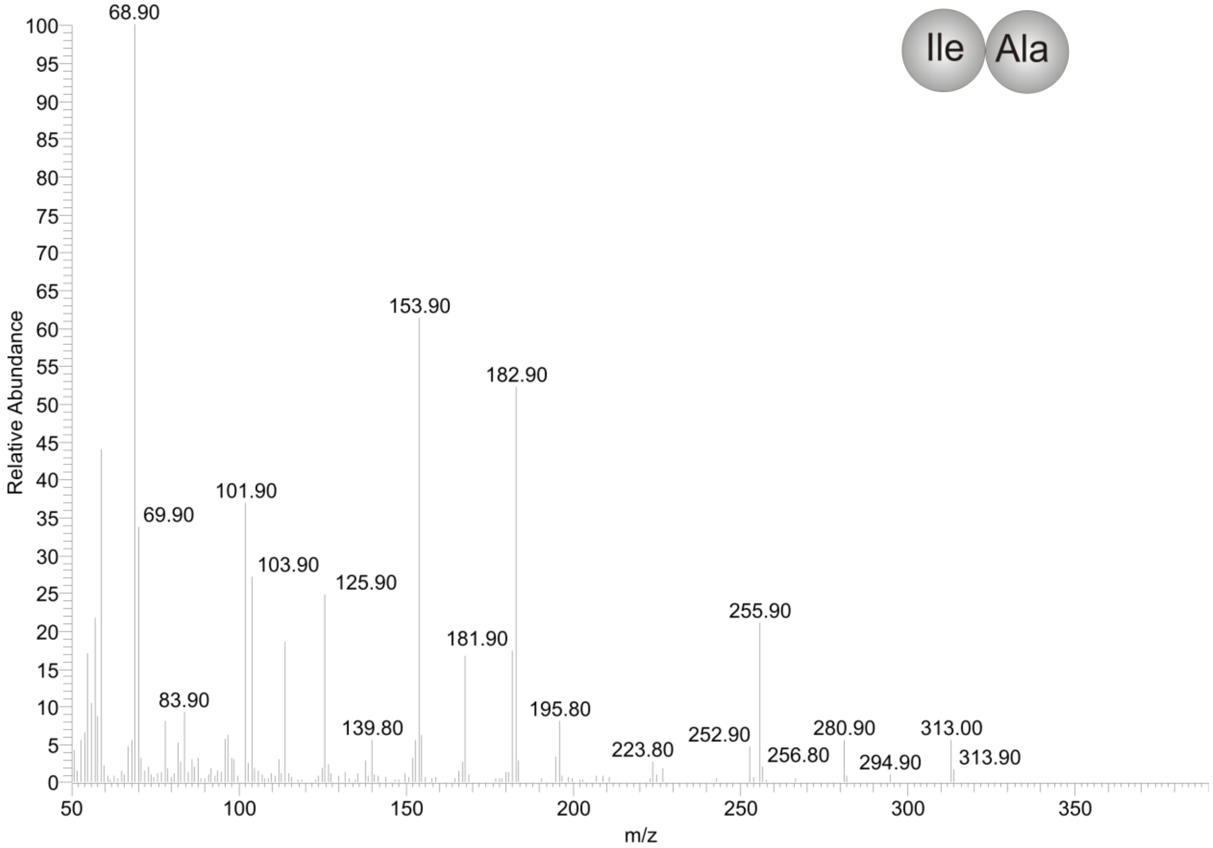

Figure S39. PCI-MS spectra of synthetic *N*-trifluoroacetyl-Leu-Phe-methyl ester.

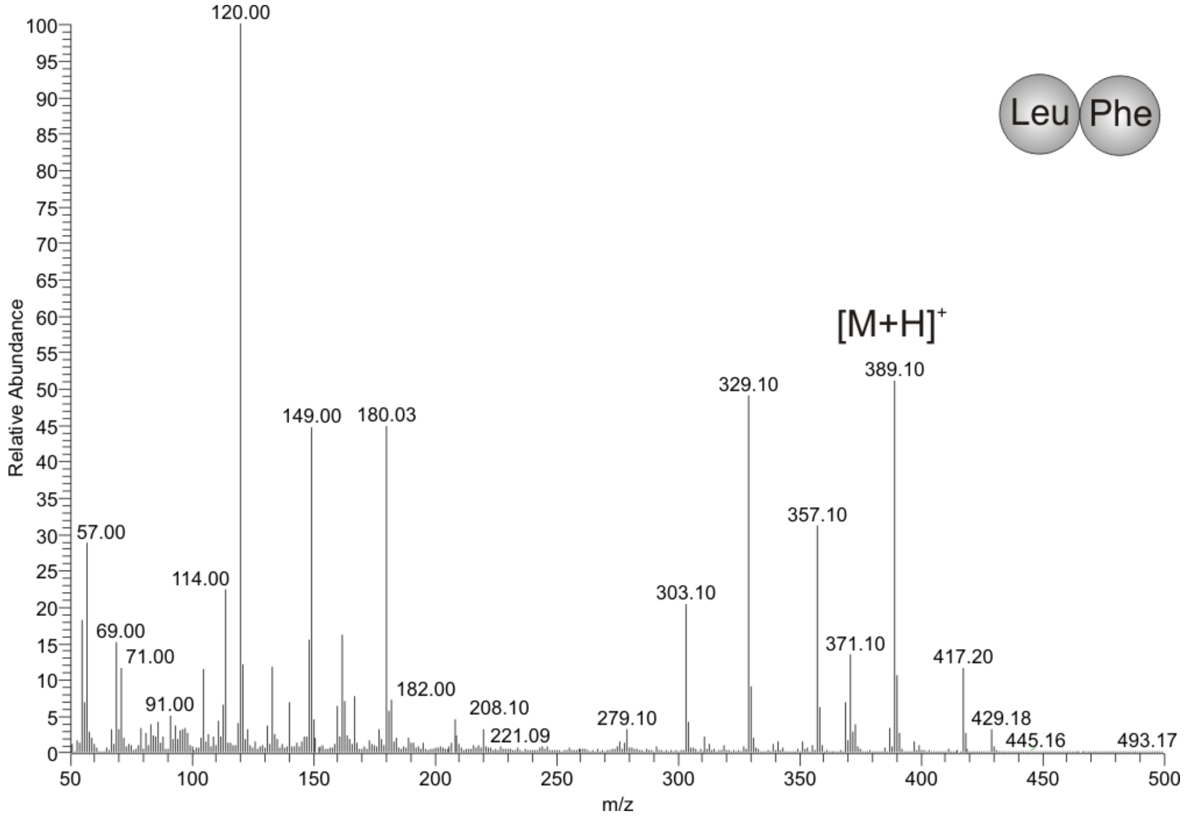

Figure S40. EI-MS spectra of synthetic *N*-trifluoroacetyl-Leu-Phe-methyl ester.

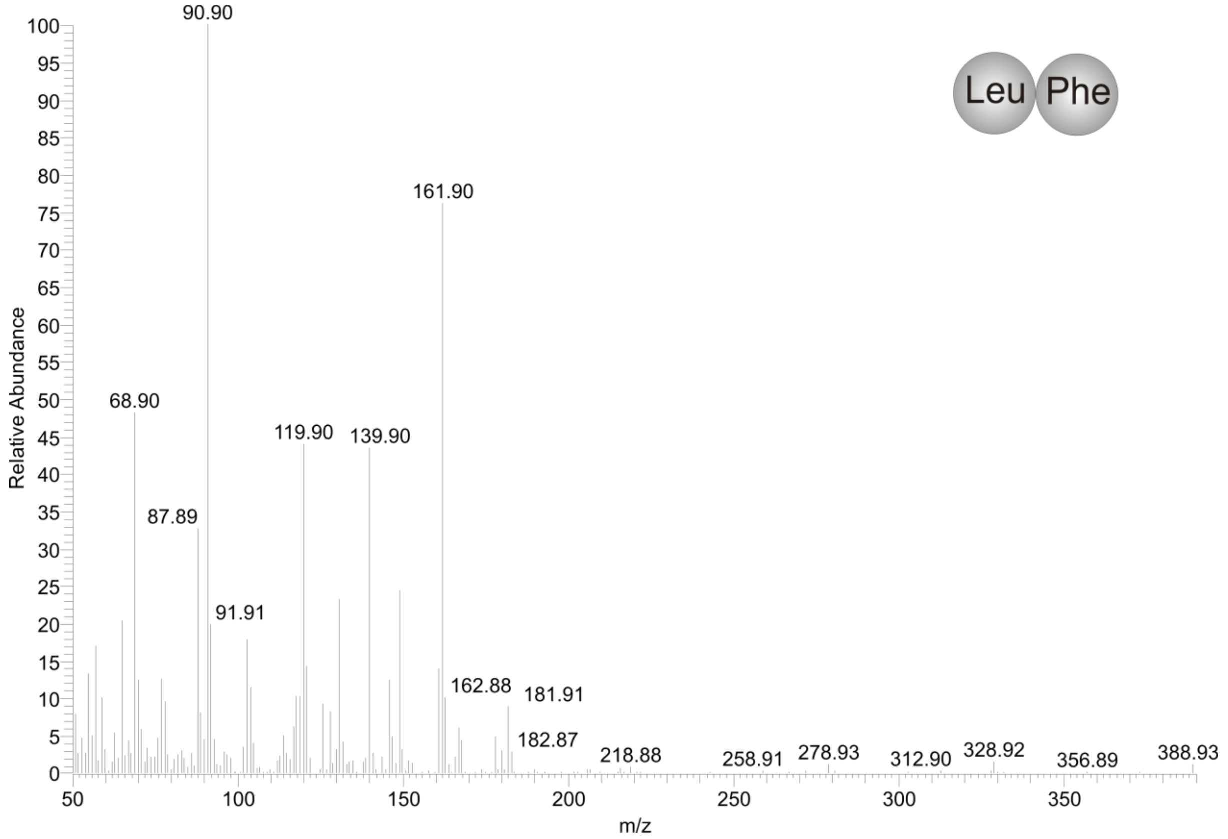

Figure S41. PCI-MS spectra of synthetic *N*-trifluoroacetyl-Phe-Leu-methyl ester.

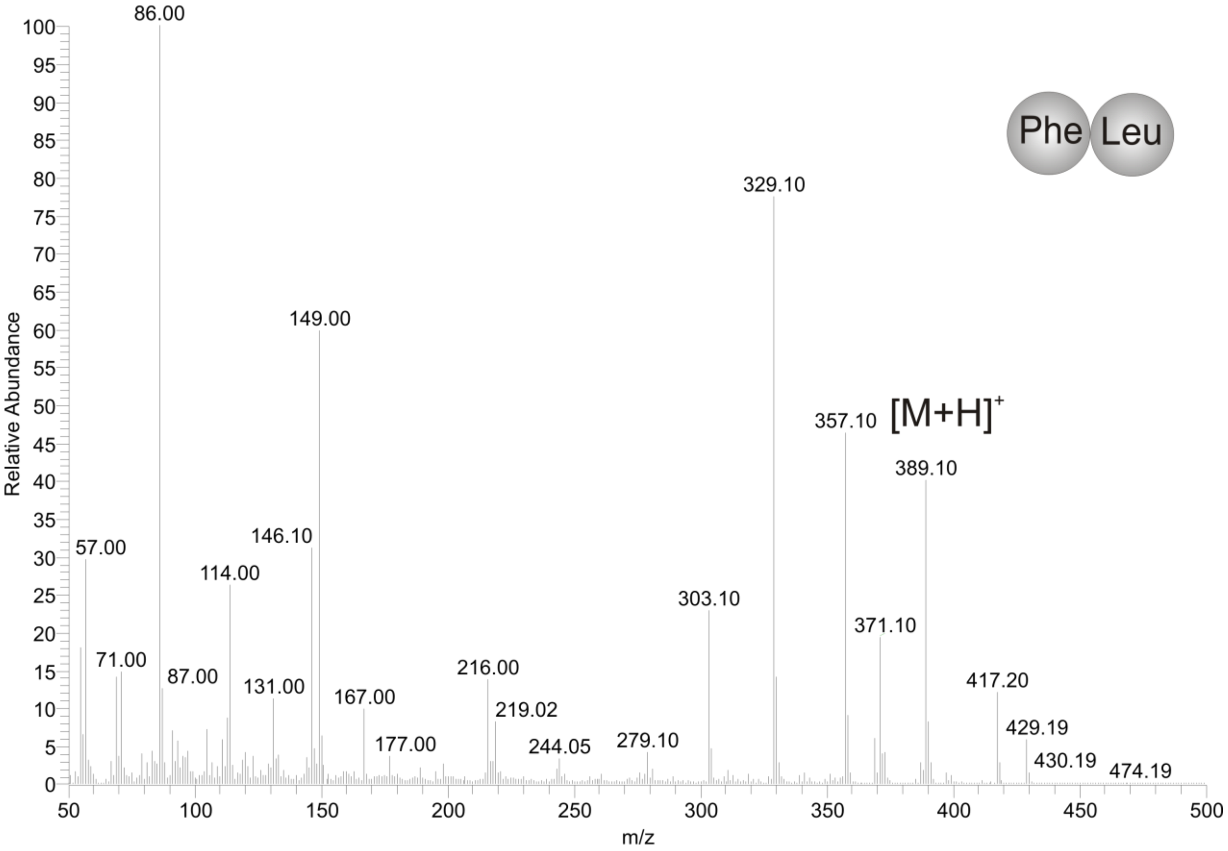

Figure S42. EI-MS spectra of synthetic *N*-trifluoroacetyl-Phe-Leu-methyl ester.

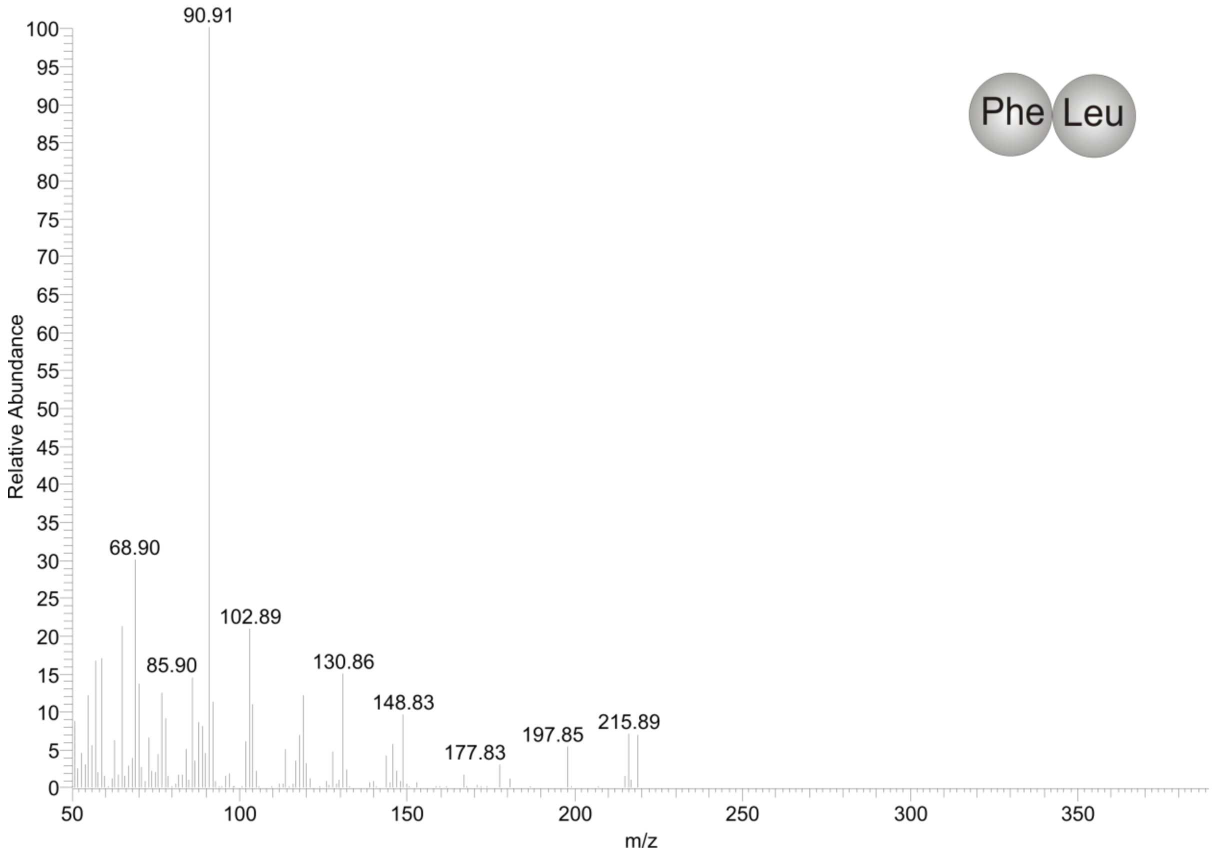

Figure S43. PCI-MS spectra of synthetic *N*-trifluoroacetyl-Ile-Phe-methyl ester.

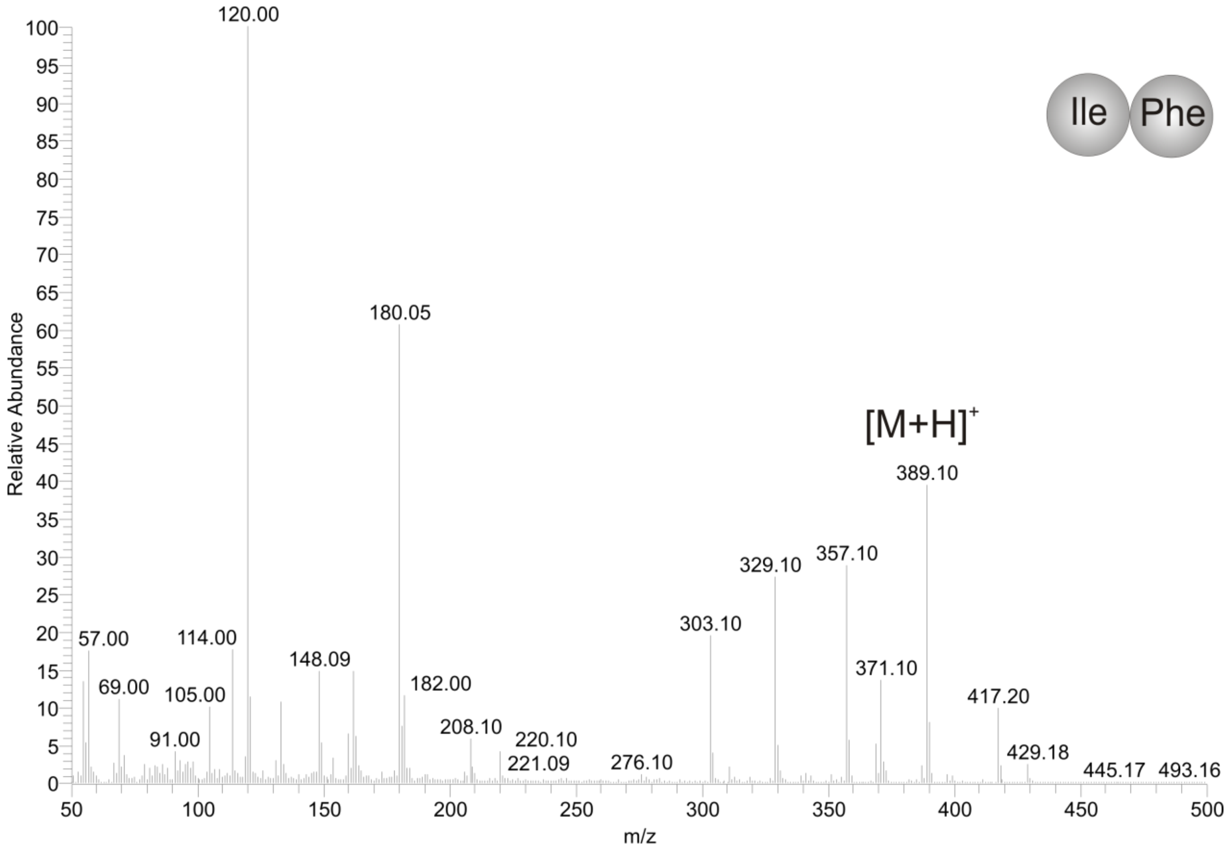

Figure S44. EI-MS spectra of synthetic *N*-trifluoroacetyl-Ile-Phe-methyl ester.

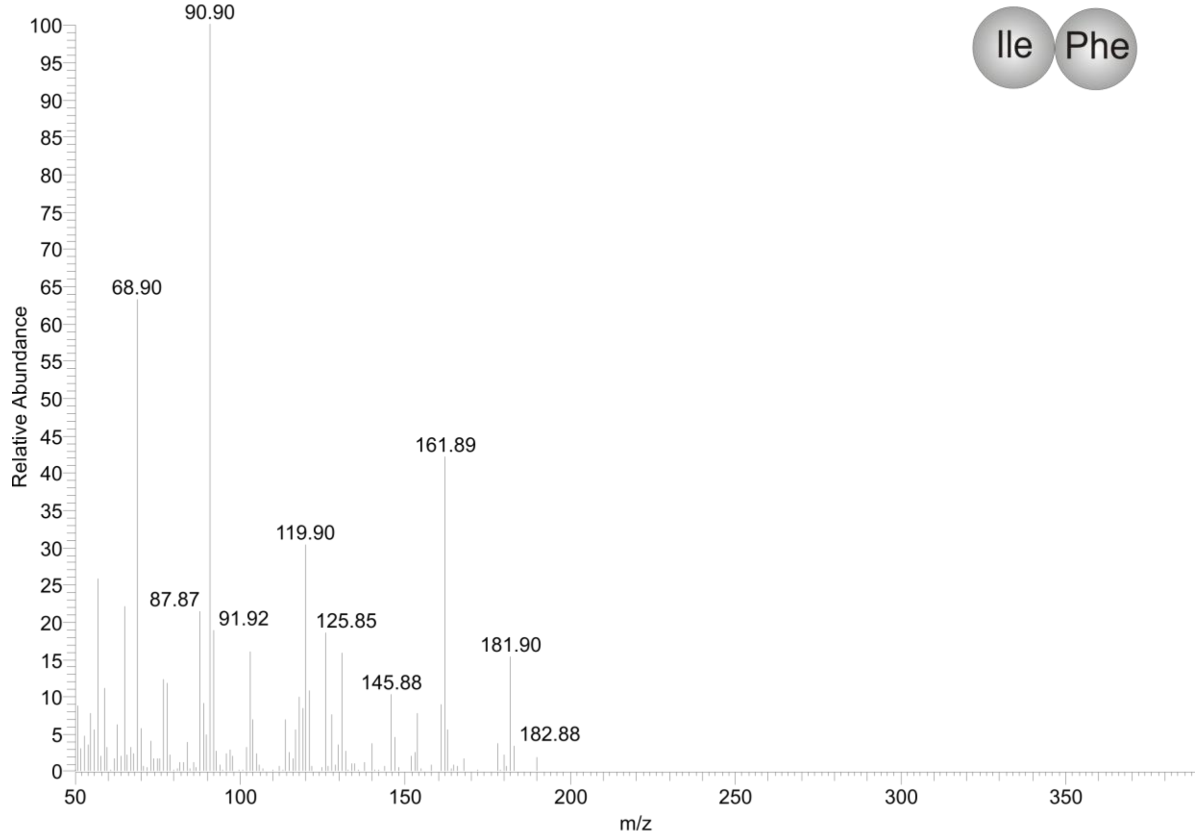

Figure S45. PCI-MS spectra of synthetic *N*-trifluoroacetyl-Phe-Ile-methyl ester.

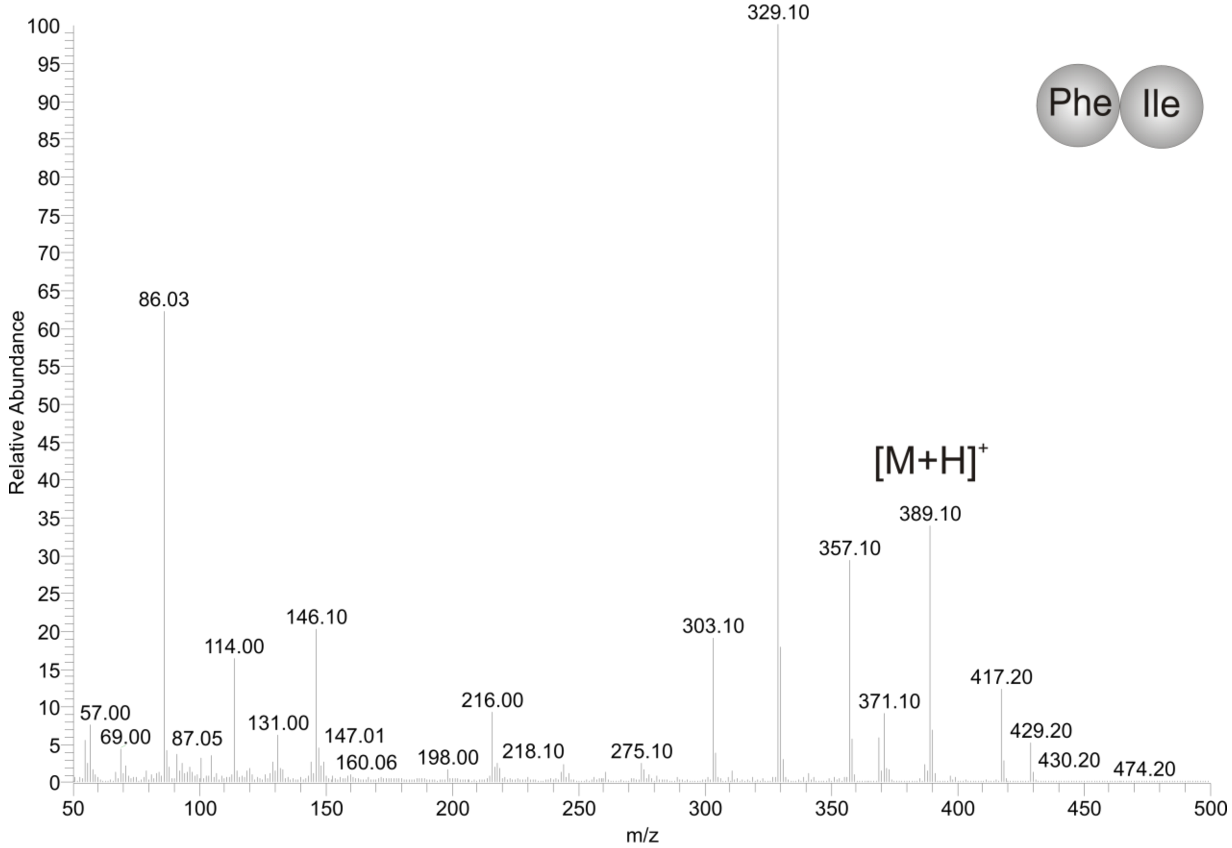

Figure S46. EI-MS spectra of synthetic *N*-trifluoroacetyl-Phe-Ile-methyl ester.

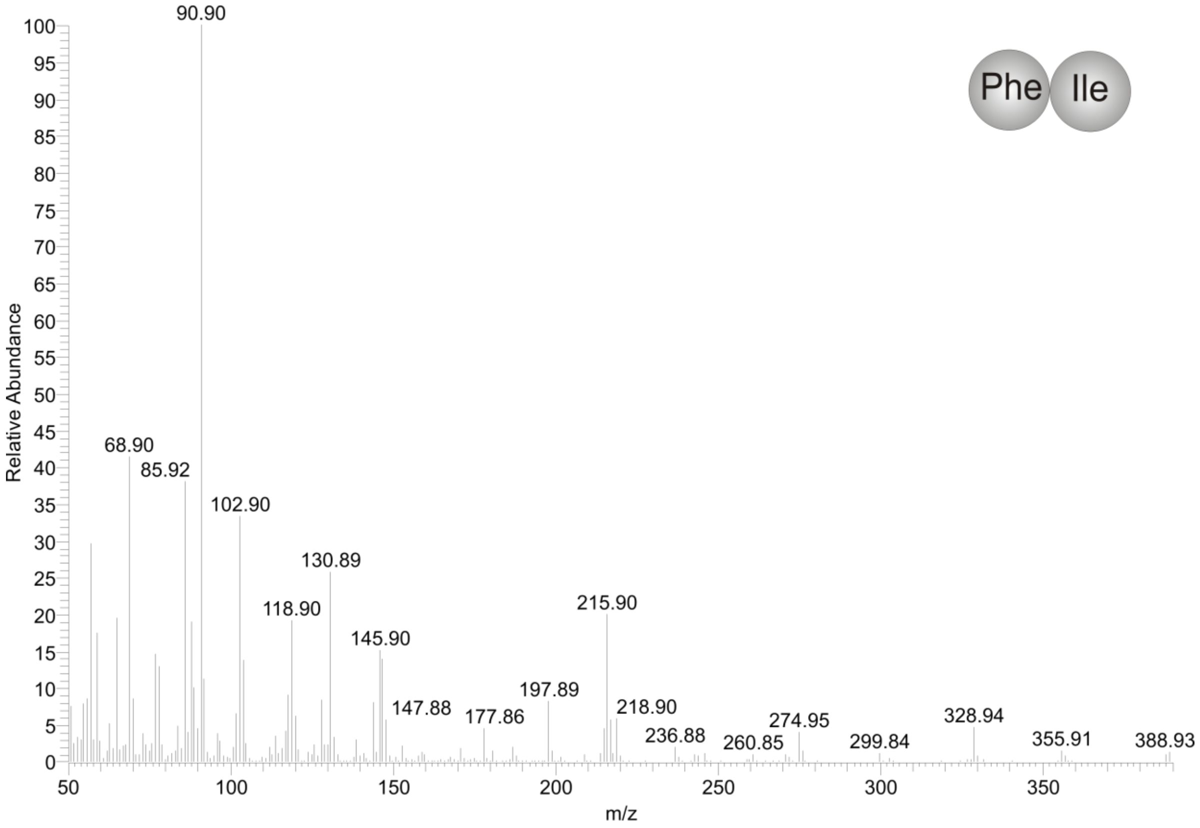

Supplement: Supplementary File 1 — Supplementary Information (PDF, 3103 KB) [file marinedrugs-11-04834-s001.pdf]
